# Clinical efficacy of joint mobilization for shoulder impingement syndrome: a systematic review and meta-analysis

**DOI:** 10.1371/journal.pone.0352260

**Published:** 2026-07-13

**Authors:** Gayeong Lee, Hyeonjun Woo, Yunhee Han, Seungkwan Choi, Jungho Jo, Junghan Lee, Seojae Jeon, Wonbae Ha

**Affiliations:** 1 Department of Korean Medicine, College of Korean Medicine, Wonkwang University, Iksan, Republic of Korea; 2 Department of Korean Medicine Rehabilitation, College of Korean Medicine, Semyung University, Jecheon, Republic of Korea; 3 Department of Korean Medicine Rehabilitation, College of Korean Medicine, Wonkwang University, Iksan, Republic of Korea; 4 Department of Korean Internal Medicine, College of Korean Medicine, Wonkwang University, Iksan, Republic of Korea; 5 Jeonbuk Advanced Bio-Convergence Academy, Wonkwang University, Iksan, Republic of Korea; 6 Korea Institute of Integrative Medicine, Jangheung, Republic of Korea; 7 Research Center of Traditional Korean Medicine, College of Korean Medicine, Wonkwang University, Iksan, Republic of Korea; Nitte (Deemed to be University), Nitte Institute of Physiotherapy (NIPT), INDIA

## Abstract

**Background:**

No systematic review has examined the clinical effectiveness of mobilization for shoulder impingement syndrome (SIS). We aimed to evaluate clinical effectiveness for the use of mobilization in SIS.

**Methods:**

PubMed, Embase and the Cochrane Library were searched for studies published up to April 30, 2023. Randomized controlled trials that evaluated the effectiveness of mobilization for SIS were included. Literature was extracted using Endnote 20, and the risk of bias was assessed using the Cochrane risk-of-bias tool 2.0. A meta-analysis was conducted using RevMan 5.4. This study was conducted in accordance with the PRISMA guidelines.

**Results:**

Nineteen randomized controlled trials (956 participants) were selected based on the inclusion criteria. Adding mobilization to traditional physical therapy reduced pain (mean difference [MD]=1.72, 95% confidence interval [CI]: 0.91 to 2.53, I^2^ = 97%, p < 0.00001), increased active range of motion (MD = 14.49, 95% CI: 8.46 to 20.53, I^2^ = 95%, p < 0.00001), and improved the disabilities of the arm, shoulder, and hand (MD = 5.95, 95% CI: 0.01 to 11.90, p = 0.05) and Constant-Murley scores (MD = 5.49, 95% CI: 0.78 to 10.19, p = 0.02). Joint mobilization was more effective in reducing pain (MD = 0.67, 95% CI: 0.15 to 1.19, p = 0.01) and increasing active range of motion (MD = 8.56, 95% CI: 2.31 to 14.82, p = 0.007) compared with sham mobilization. No statistically significant differences were found between physical therapy plus mobilization and physical therapy plus exercise.

**Conclusion:**

Mobilization may offer adjunctive, short-term benefits in improving the clinical symptoms of SIS when added to conventional treatments. Future studies should focus on evaluating the effects of mobilization as a stand-alone intervention, incorporate larger sample sizes and long-term follow-up, and conduct high-quality comparative trials to identify the most effective specific mobilization techniques.

## Introduction

Musculoskeletal disorders, which affect more than 1.7 billion people worldwide, are often associated with persistent pain, functional limitations, disability, loss of productivity, and decreased quality of life. Among these, shoulder pain is one of the most common symptoms, with prevalence rates ranging from 7% up to 26% [1]. Shoulder impingement syndrome (SIS), a concept first introduced by Neer in 1972, is a general term for injuries to the subacromial space structures, such as rotator cuff tendinitis and bursitis. It is the most common cause of shoulder pain, accounting for 36% of shoulder disorders [2,3]. SIS can be classified into primary and secondary types. Primary impingement is caused by an increase in soft tissue volume due to subacromial bursitis, calcific tendonitis, and mechanical narrowing of the subacromial space caused by bone deformation. Secondary impingement is caused by functional disorders, such as muscle imbalance, which leads to abnormal movement and soft tissue entrapment. The incidence of SIS is highest among individuals in their 60s, and its prevalence increases with age [4].

The typical sign of SIS is pain localized to the anterolateral acromion area or radiating to the lateral midhumerus, which increases during overhead activities. Nocturnal pain while lying on the affected side and an overall loss of strength may be present, and decreased function due to pain can also be problematic [4,5]. Therefore, the main objective in treating SIS is to eliminate pain and restore joint function. If no significant structural damage is identified, the initial treatment involves a combination of conservative measures lasting for 3–6 months. Conservative treatment options include immobilization, non-steroidal anti-inflammatory drugs, cortisone injections, manual therapy, physiotherapy, ultrasound, heat, and electrotherapy. These interventions aim to initially relieve acute pain and gradually increase joint mobility. More than 70% of cases improve with conservative treatment. However, if symptoms persist after more than 3 months of conservative treatment, surgery becomes necessary, mainly subacromial decompression, bursectomy, or acromioplasty [4].

Joint mobilization, a type of manual therapy, involves low-velocity, high-amplitude movements within a joint’s range of motion (ROM) [6]. In general, mobilization improves hypomobility, enhances joint function, and alleviates pain. In addition to joint mobilization, mobilization includes nerve and soft tissue mobilization. Joint mobilization improves circulation within the joint, increases joint extensibility, and breaks down adhesions in narrowed soft tissues, thereby minimizing joint inflammation, swelling, and pain, while simultaneously improving ROM [7,8]. The main neurophysiological mechanisms of joint mobilization discussed to date include mechanoreceptor stimulation, endorphin release, and decreased cytokine concentrations [9]. Neuromobilization is a treatment that aims to restore neurological function by performing specific physical movements that generate targeted mechanical events in the nervous system [10]. Similarly, soft tissue mobilization involves applying mechanical stimulation to soft tissues to break down existing adhesions and restrictions [11]. These mobilizations can be applied to patients with SIS to improve shoulder joint pain and restore joint function. In research settings, sham mobilization is utilized as a placebo control, involving manual contact by the therapist without delivering the actual therapeutic force or specific joint movement. However, to determine whether these therapeutic effects are truly attributable to the mobilization itself, the use of sham mobilization as a comparison intervention is particularly important, as it helps control for placebo effects such as therapist attention and physical contact in manual therapy studies. Introducing sham mobilization allows for a more accurate assessment of the true therapeutic effects of mobilization techniques.

To date, studies have been conducted on the treatment of SIS using exercise therapy [1], mobilization with movement (MWM) technique [12], and conventional physical therapy sets [3,5]. No systematic review has examined the effects of mobilization as a whole on SIS. Furthermore, studies have reported the effectiveness of mobilization for various conditions, including ankle sprains [13], ankle joint instability [14], knee osteoarthritis [15], carpal tunnel syndrome [16], and frozen shoulder [17,18].

A number of randomized controlled trials (RCTs) have suggested that mobilization may play an important role in normalizing shoulder capsular structure in SIS by reducing capsular swelling or pain and resolving abnormal collagen binding and adhesions [19]. Based on these RCTs, this systematic review was conducted to analyze and evaluate the clinical evidence on whether mobilization has a significant therapeutic effect on improving the symptoms of SIS. Although mobilization techniques differ in their specific biomechanical and neurophysiological mechanisms, they share a common therapeutic goal of improving pain and movement dysfunction. Therefore, evaluating mobilization as a broader clinical category may provide useful insight into its overall role as an adjunctive treatment in patients with shoulder impingement syndrome.

## Methods

This systematic review was registered with PROSPERO (CRD42023418388). This study was conducted according to the Cochrane Handbook for Systematic Reviews of Interventions [20]. This systematic review followed the PRISMA guideline [21] (see S1 File in S1 Text).

### Database selection and search

PubMed, Embase, and the Cochrane Library databases were searched for English-language clinical studies applying mobilization to SIS, including all articles published from the inception of the databases through April 2023. These English-language online databases were searched using a combination of the following search terms: “shoulder impingement syndrome,” “rotator cuff,” “impingement,” “subacromial impingement,” and “mobilization,” with search formulas modified to each database. The detailed search formulas are provided in S2 File in S1 Text.

### Inclusion and exclusion criteria

RCTs on mobilization alone or in combination with other interventions for SIS were selected from the retrieved literature. Studies with the terms “mobilization” and “impingement” in the title and abstract were included in the search results.

#### Inclusion criteria.

Articles included in this systematic review were selected based on the participants, interventions, comparisons, outcomes, and study designs framework.

(1) Participants (P): Patients with SIS (2) Interventions (I): The experimental group received the mobilization therapy alone or other interventions in addition to the mobilization. (3) Control (C): The control group received treatments other than the mobilization. If the experimental group has additional interventions beyond the mobilization, the control group has no interventions other than the same intervention. (4) Outcome (O): Diagnostic metrics in SIS, including VAS, ROM, DASH, SPADI, constant score, and more. (5) Study design (S): Only randomized controlled trials were included.

#### Exclusion criteria.

Studies were excluded if they met any of the following criteria:

(1) Participants (P): The study population did not consist of individuals diagnosed with SIS. (2) Interventions (I): The mobilization group received additional concurrent interventions that could not be isolated from mobilization. (3) Control (C): The comparison group received interventions that overlapped with mobilization that could not be separated (e.g., complex manual therapy packages where the specific effects of mobilization could not be isolated), or no appropriate control group was provided. (4) Outcome (O): The study did not report relevant clinical outcomes related to SIS, such as pain, range of motion, or functional measures. (5) Study design (S): Non-randomized controlled trials (non-RCTs), observational studies, case reports, and other study designs that did not meet the methodological requirements for RCT classification.

In the retrieved articles, no restrictions were applied regarding participants (age, sex), interventions (types or techniques of mobilization), comparisons (composition of the control group), outcomes (indices used for assessment), or study characteristics (treatment duration, follow-up period) in the retrieved articles. Additionally, we did not exclude participants based on the type of SIS (primary or secondary) or their medical history (e.g., previous surgeries or fractures).

### Literature selection and analysis

All retrieved references were exported to EndNote 20, where duplicate records were removed before screening. Literature search and selection were independently conducted by two researchers (GL and WH). In cases of disagreement, the researchers engaged in discussions to reach a consensus. Initially, the titles and abstracts of the retrieved articles were screened. Subsequently, the study design, target patients, interventions, control groups, indices, and outcomes of the selected articles were analyzed, summarized, and reviewed in detail. Studies that met the inclusion and exclusion criteria were then selected (see S3 File in S1 Text).

### Literature analysis and assessment of risk of bias

For literature analysis, two researchers (GL and WH) reviewed the full text of the selected articles, extracted information, including study design, and tabulated the applied interventions, control groups, indices, and main outcomes. In cases of disagreement between the two independent researchers during data extraction, a third researcher (JL) was consulted.

The risk of bias in the RCTs was assessed by two independent researchers (GL and WH) using the ROB 2.0 tool. This tool assesses bias across five domains that could affect study results. Specifically, it examines bias in the randomization process, deviations from intended interventions, missing outcome data, outcome indices, and the selection of reported results. Identification of bias is based on both empirical and theoretical considerations. The tool uses an algorithm to guide users in determining the overall risk of bias in a study – categorized as “low risk,” “some concern,” or “high risk” – based on responses to signal questions within each domain [22]. Any disagreements between assessors were resolved through discussion with a third researcher (JL) to reach consensus.

### Operational definitions of interventions

In this study, ‘mobilization’ was used as an umbrella term for various manual therapy techniques applied to the shoulder joint and its surrounding structures. The primary techniques identified in the included studies are defined as follows. Joint mobilization refers to low-velocity, passive movements applied by a therapist to a patient’s joint to restore mobility and reduce pain, focusing on physiological and accessory motions. Neuromobilization involves the application of mechanical forces to the nervous system through specific body movements to restore neural function. Soft tissue mobilization is a technique that applies mechanical stimulation to soft tissues like muscles and fascia to break down adhesions and relieve restrictions. More specific methods included Mobilization with Movement (MWM), a technique combining a sustained accessory joint glide by the therapist with the patient’s active physiological movement, and Dynamic Humeral Centering (DHC), a manual technique aimed at correcting the position of the humeral head within the glenoid fossa during movement to reduce subacromial impingement. These techniques, while sharing the overarching goal of restoring mobility, operate through distinct biomechanical and neurophysiological mechanisms, contributing to conceptual heterogeneity. Various modalities such as TENS, ultrasound, and heat therapy were grouped under a single ‘physical therapy’ category for analysis, as they share the common physiological goals of standard conservative care (e.g., pain relief, tissue healing) and are routinely administered together in clinical settings.

### Data extraction

The full text of the selected articles was reviewed, and the following data were extracted for each article: author, year, intervention, control group, number of subjects, indices, and outcomes. The meta-analysis was conducted using Cochrane’s Review Manager (RevMan) version 5.4 (Copenhagen, Denmark). Continuous outcomes were analyzed using the mean difference (MD) and 95% confidence interval (CI). For effect size analysis, we used SMD rather than OR or RR. Heterogeneity was assessed with Higgins’ I^2^ values, where values below 50% were considered indicative of low heterogeneity, and those above 50% indicated high heterogeneity. In addition to the I^2^ assessment for heterogeneity, we used a Random-Effects Model due to anticipated clinical and methodological heterogeneity, which allows for differences in true effect sizes between studies and is used when there is heterogeneity, and is weighted using the inverse variance method.

In one of the studies included in this review [9], missing data were observed for certain variables, which amounted to approximately 0.01% of the total data and were considered to be missing at random. Therefore, we used a complete-case analysis approach that excluded missing data Publication bias assessments (e.g., funnel plots) were not performed as no meta-analysis included 10 or more studies.

### Data consolidation and analysis

For a quantitative synthesis of the results, a meta-analysis was conducted. Inclusion in the meta-analysis was limited to studies that provide sufficient statistical data (specifically means, standard deviations, and sample sizes) for clinically comparable intervention and control groups.

## Results

### Literature selection

**Fig 1:**
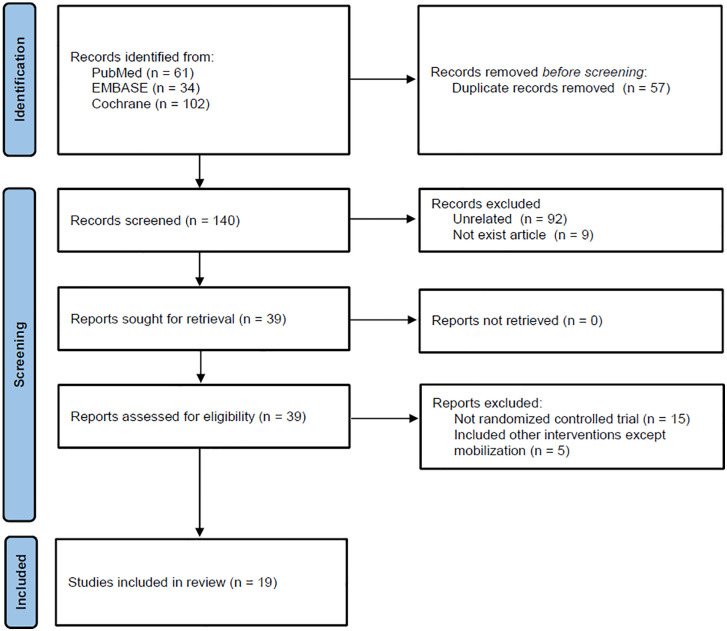
PRISMA flow diagram.

In total, 197 articles were retrieved from the three online databases through April 2023, with 57 duplicates subsequently excluded. Titles and abstracts were then screened to exclude 92 irrelevant articles and 9 with unavailable full text, resulting in 101 exclusions. We also excluded 15 articles that were not RCTs. Furthermore, we excluded five studies with interventions other than mobilization in the mobilization group after excluding other overlapping interventions in both the mobilization and comparison groups, resulting in a final selection of 19 articles (Fig 1).

### Literature analysis

#### Research overview.

The 19 finalized RCTs evaluated 956 shoulders. All 19 studies used mobilization as the primary intervention, with nine studies comparing mobilization plus specific physical therapy with specific physical therapy alone. Exercise therapy (guided exercise (GE) and home exercise (HE)) was the most common intervention in the comparison groups, followed by short-wave diathermy (SWD) electrotherapy (ET), ultrasonic therapy (US), transcutaneous electrical nerve stimulation (TENS), neuromuscular electrical stimulation (NMES), hot pack (HP), and cryotherapy. The sham mobilization group was the other main comparison group. Other inclusion criteria included studies involving the crossover of physiotherapy with mobilization and sham mobilization at 4-week intervals, studies comparing the effects of mobilization with proprioceptive neuromuscular facilitation (PNF), studies applying mobilization on both the neck and shoulder versus the shoulder alone, and studies investigating the effects of mobilization after subacromial corticosteroid (SACS) injection (Table 1). The detailed data for the synthesis is summarized in S4 File in S1 Text.

**Table 1:** Clinical studies on the use of mobilization on shoulder impingement syndrome.

Study	patients / mean Age	interventions	Dose	Outcome index	Main Findings
Akhtar [10] (2020)	Total 80 A: 36.83 ± 8.93 (n = 40) B: 34.40 ± 9.32 (n = 40)	A: Neuromobilization + SWD + US + TENS + GE b: SWD + US + TENS + GE	3 per week × 5 weeks	(1) VAS (10) (2) UCLA rating score	Significant improvement in all outcomes for group A compared to group B. at 11th week.
Aytar [23] (2015)	Total 66 A: 52 ± 3 (n = 22) B: 52 ± 4 (n = 22) C: 51 ± 4 (n = 22)	A: Scapular mobilization + TENS + HP + HE B: Sham scapular mobilization + TENS + HP + HE c: GE + TENS + HP + HE	3 per week × 3 weeks	(1) Quick DASH (2) VAS (10) (3) shoulder AROM (4) Participant Satisfaction	No significant difference in any outcome for Group A compared to group B, C. No significant difference in any outcome for Group A compared to group B, C.
Beaudreuil [24] (2011)	Total 70 A: 59.4 ± 10.0 (n = 35) B: 57.9 ± 10.7 (n = 34)	A: Dynamic humeral centering + HE B: Non-specific (sham) mobilization + HE	3 per week × 1^st^ 3 weeks 2 per week × 2^nd^ 3 weeks	(1) Constant score (2) Medication	Significant improvement in pain score and medication use for group A compared to group B. Significant improvement in pain score and medication use for group A compared to group B.
Conroy [19] (1998)	Total 14 A: 50.7 ± 16.5 (n = 7) B: 55.0 ± 10.2 (n = 7)	A: Glenohumeral mobilization + HP + HE + STM b: HP + HE + STM	3 per week × 3 weeks	(1) VAS (100) (2) AROM	Significant improvement in pain over a 24 hour period and with subacromial compression testing for group A compared to group B. Significant improvement in pain over a 24 hour period and with subacromial compression testing for group A compared to group B.
Cook [25] (2014)	Total 68 A: 54.1 (n = 36) B: 51.0 (n = 32)	A: Mobilization (neck and shoulder) + GE B: Mobilization (shoulder only) + GE	A: 9.6 sessions × 59.7 days B: 8.9 sessions × 52 days	(1) Quick DASH (2) NPRS (10) (3) PASS	No significant difference in any outcome for Group A compared to group B. No significant difference in any outcome for Group A compared to group B.
Delgado-Gil [26] (2015)	Total 42 A: 55.4 ± 7.8 (n = 21) B: 54.3 ± 10 (n = 21)	A: Mobilization (MWM) B: Sham manual contact	2 per week × 2 weeks	(1) NPRS (10) (2) AROM	Significant improvement in pain during shoulder flexion, pain-free range of shoulder flexion motion, maximal shoulder flexion, and maximal external rotation for group A compared to group B. Significant improvement in pain during shoulder flexion, pain-free range of shoulder flexion motion, maximal shoulder flexion, and maximal external rotation for group A compared to group B.
Eliason [9] (2021)	Total 120 A: 43.2 (n = 29) B: 45.5 (n = 52) C: 46.0 (n = 39)	A: Joint mobilization + GE + HE B: GE + HE C: No treatment	Mobilization : 1–2 per week × 8 sessions × 1st 6 weeks Exercise : 2 per week × 20 sessions × 12 weeks	(1) Constant-Murley score (2) VAS(100) in AROM	Significant improvement in subscore pain and total score in Contstant-Murley score for group A, B compared to group C. Significant improvement in subscore pain and total score in Contstant-Murley score for group A, B compared to group C. Significant improvement in pain in pain-free range of shoulder flexion in short-term for group A compared to group B, C. Significant improvement in pain in pain-free range of shoulder flexion in short-term for group A compared to group B, C.
Guimarães [27] (2016)	Total 27 A: 30.3 ± 6.9 (n = 14) B: 31.9 ± 9.2 (n = 13)	A: 4 sessions of mobilization (MWM) → 4 sessions of sham technique B: 4 sessions of sham technique → 4 sessions of mobilization(MWM)	1 per week × 8 weeks	(1) AROM (2) Isometric Peak Force (3) DASH (4) SPADI	Significant improvement in SPADI for group A compared to group B. Significant improvement in SPADI for group A compared to group B. Significant improvement in DASH for group B compared to group A. Significant improvement in DASH for group B compared to group A.
Gutiérrez-Espinoza [28](2023)	Total 72 A: 45.2 (n = 36) B: 44.5 (n = 36)	A: Scapular mobilization + GE B: GE	2 per week × 6 weeks	(1) DASH (2) Constant-Murley score (3) VAS (10) (4) scapular UR	No significant difference in any outcome for Group A compared to group B. No significant difference in any outcome for Group A compared to group B.
İğrek [29] (2022)	Total 44 A: 44.4 ± 11 (n = 15) B: 47.6 ± 12.4 (n = 15) C: 45.9 ± 9.7 (n = 14)	A: Scapular mobilization + ET + GE b: PNF + ET + GE B: ET + GE	5 per week × 4 weeks	(1) VAS (10) (2) DASH (3) Constant-Murley score (4) AROM (5) Muscle strength	Significant improvement in all outcome except pain at rest and range of external rotation for Group A, B to group C. Significant improvement in all outcome except pain at rest and range of external rotation for Group A, B to group C.
Kachingwe [30] (2008)	Total 33 A: 43.4 ± 14.7 (n = 9) B: 38.9 ± 13.7 (n = 9) C: 47.3 ± 20.1 (n = 8) D: 45.6 ± 13.0 (n = 7)	A: Mobilization of Glenohumeral joint + GE B: MWM + GE C: GE D: Unsupervised Exercise, advice	1 per week × 6 weeks	(1) VAS (2) AROM (3) SPADI	No significant difference in any outcome for Group A compared to group B. No significant difference in any outcome for Group A compared to group B.
Kulakli [31] (2020)	Total 84 A: 51.20 ± 8.01 (n = 42) B: 49.35 ± 9.75 (n = 42)	A: Joint mobilization after SACS injection + HE B: SACS injection + HE	1 session	(1) AROM (2) VAS (10) (3) DASH	Significant improvement in range of shoulder flexion and abduction motion, and pain with activity in short-term for group A compared to group B. Significant improvement in range of shoulder flexion and abduction motion, and pain with activity in short-term for group A compared to group B.
Menek [32] (2019)	Total 30 A: 51.73 ± 6.64 (n = 15) B: 50.26 ± 4.28 (n = 15)	A: Mulligan mobilization (MWM) + GE + Cold Pack + US + TENS B: GE + Cold Pack + US + TENS	5 per week × 6 weeks	(1) VAS (100) (2) shoulder AROM (3) DASH (4) SF-36	Singnificnat improvement in pain, AROM, DASH, and some parameters of SF-36 for group A compared to group B. Singnificnat improvement in pain, AROM, DASH, and some parameters of SF-36 for group A compared to group B.
Neelapala [33] (2016)	Total 31 A: 40.23 ± 10.55 (n = 15) B: 42.41 ± 10.38 (n = 16)	A: Shoulder mobilization (MWM) + GE B: GE	3 sessions	(1) VAS (10) (2) Scapular UR (3) Muscle strength	Significant improvement in pain and external rotator strength for group A compared to group B. Significant improvement in pain and external rotator strength for group A compared to group B.
Park [34] (2020)	Total 30 A: 49.20 ± 9.48 (n = 10) B: 50.90 ± 9.10 (n = 10) C: 50.20 ± 8.99 (n = 10)	A: Thoracic mobilization (15 min) B: GE (15 min) C: Combination (M 7m 30s, E 7m 30s)	3 per week × 4 weeks	(1) Thoracic kyphosis angle (2) Muscle tone (3) muscle stiffness (4) PROM (5) SPADI	Significant improvement in all outcome except muscle stiffness for group C compared to group A, B. Significant improvement in all outcome except muscle stiffness for group C compared to group A, B.
Pekgöz [35] (2019)	Total 40 A: 45.2 ± 6.5 (n = 20) B: 41.9 ± 9.0 (n = 20)	A: Shoulder joint mobilization + NMES B: GE + NMES	3 per week × 4 weeks	(1) PROM (2) VAS (10) (3) DASH (4) ASES (5) SF-36	No significant difference in any outcome for Group A compared to group B. No significant difference in any outcome for Group A compared to group B.
Satpute [36] (2014)	Total 44 A: 53.41 ± 7.08 (n = 22) B: 52.41 ± 7.06 (n = 22)	A: Mobilization (MWM) + HP + HE b: GE + HP + HE	3 per week × 3 weeks	(1) pain free HBB (2) VAS (10) in HBB (3) PROM (4) SPADI(%)	Significant improvement in all outcome for group A compared to group B. Significant improvement in all outcome for group A compared to group B.
Srivastava [37] (2018)	Total 22 A: 41.91 (n = 11) B: 50.09 (n = 11)	A: mobilization (MWM) + GE B: Cold Pack + GE	1 per day × 6 days	(1) VAS (10) (2) AROM (3) SPADI(%)	No significant difference in any outcome for Group A compared to group B. No significant difference in any outcome for Group A compared to group B.
Surenkok [38] (2009)	Total 39 A: 55.07 ± 13.36 (n = 13) B: 54.30 ± 12.70 (n = 13) C: 55.53 ± 17.15 (n = 13)	A: Scapular mobilization B: Sham mobilization C: The control (nothing)	1 session	(1) AROM (2) Scapular UR (3) VAS (100) (4) Constant score	Significant improvement in active range of flexion and abduction, scapular upward rotation, and constant shoulder score for group A compared to group B, C. Significant improvement in active range of flexion and abduction, scapular upward rotation, and constant shoulder score for group A compared to group B, C.

SWD, short wave diathermy; US, ultrasonic therapy; TENS, transcutaneous electrical nerve stimulation; GE. guided exercise; VAS, visual analog scale; UCLA, university of California, Los Angeles; HP, hot pack; HE, home exercise; DASH, disability of the arm, shoulder and hand questionnaire; AROM, active range of motion; STM, soft tissue mobilization; NPRS, numeric pain rating scale; PASS, patient acceptable symptom state; MWM, mobilization with movement; SPADI, shoulder pain and disability index; UR, upward rotation; ET, electrotherapy; PNF, proprioceptive neuromuscular facilitation; SACS, subacromial corticosteroid; SF-36, short form 36 health survey; PROM, passive range of motion; NES, neuromuscular electrical stimulation; ASES, american shoulder and elbow surgeons; HBB, hand behind back.

#### Outcome indices.

Nineteen RCTs used 21 indices. Among the outcome indices, shoulder joint active range of motion (AROM) was utilized in 10 studies, shoulder joint passive range of motion (PROM) in three studies, scapular upward rotation (UR) in three studies, and hand behind the back (HBB) range in one study. The disabilities of the arm, shoulder, and hand questionnaire (DASH) was used in six studies, QuickDASH in two studies, shoulder pain and disability index (SPADI) and Constant-Murley score in five studies, 36-item short form health survey (SF-36), UCLA shoulder rating score (for shoulder function), and seven-point Likert scale for patient satisfaction, medication, and patient acceptable symptom state (PASS) in two studies, and American shoulder and elbow surgeons (ASES) score in one study. For symptom assessment, 14 studies used the visual analog scale (VAS) as the outcome index, followed by the numeric pain rating scale (NPRS) and muscle strength in two studies, and isometric peak force, thoracic kyphosis angle, muscle tone, and muscle stiffness in one study each.

### Treatment effects

#### Comparisons of mobilization plus physical therapy and physical therapy alone.

A between-group analysis was performed in eight studies comparing mobilization plus specific physical therapy with physical therapy alone [9,10,19,28–30,32,33]. For simplicity, we refer to the group receiving mobilization in addition to physical therapy as the add-on group and the group receiving physical therapy alone as the PT group. Across these trials, several studies reported greater improvements in pain, function, and, in some cases, muscle strength in the add-on group, whereas others found no statistically significant differences between groups. Overall, these findings suggest a trend favoring the add-on approach, although results were not entirely consistent.

**Fig 2:**
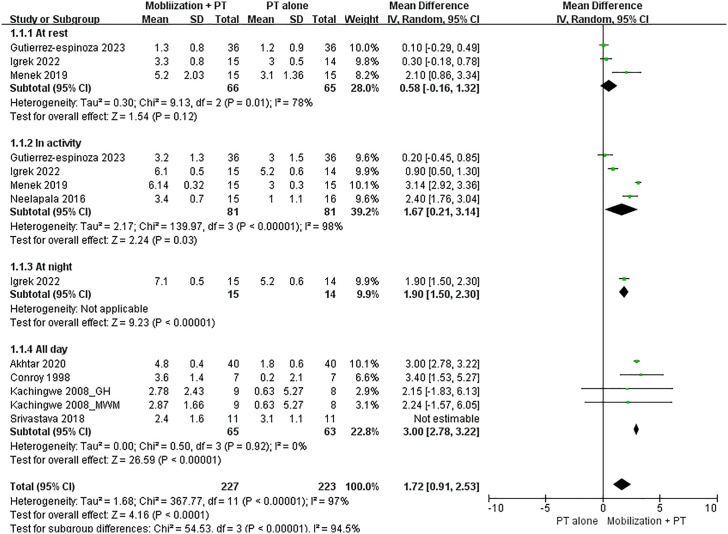
Meta-analysis of visual analog scale (VAS) outcomes between the mobilization plus physical therapy and physical therapy alone groups. SD: standard deviation; CI: confidence interval.

A meta-analysis of eight studies using VAS immediately after treatment showed a statistically significant reduction in pain in the add-on group compared with the PT group (MD = 1.72 [95% CI 0.91, 2.53], p < 0.00001, I^2^ = 97%). Given the high heterogeneity, these results should be interpreted with caution. Subgroup analyses of VAS at different time points showed no statistically significant difference at rest (MD = 0.58 [95% CI −0.16, 1.32], p = 0.12, I^2^ = 78%), whereas significant improvements were observed during activity (MD = 1.67 [95% CI 0.21, 3.14], p = 0.03, I^2^ = 98%) and across all days (MD = 3.00 [95% CI 2.78, 3.22], p < 0.00001, I^2^ = 0%) (Fig 2).

**Fig 3:**
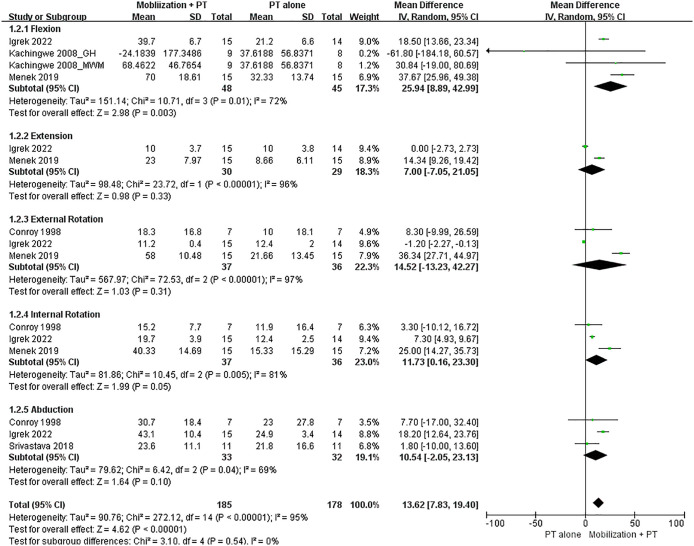
Meta-analysis of active range of motion (AROM) outcomes between the mobilization plus physical therapy and physical therapy alone groups. SD: standard deviation; CI: confidence interval.

A meta-analysis of four studies using AROM immediately after treatment demonstrated a statistically significant improvement in the add-on group (MD = 14.49 [95% CI 8.46, 20.53], p < 0.00001, I^2^ = 95%). Subgroup analyses by movement direction showed significant improvements in flexion (MD = 25.94 [95% CI 8.89, 42.99], p = 0.003, I^2^ = 72%), internal rotation (MD = 11.73 [95% CI 0.16, 23.30], p = 0.05, I^2^ = 81%), and abduction (MD = 17.69 [95% CI 12.27, 23.11], p < 0.00001, I^2^ = 0%), whereas no statistically significant differences were observed in extension (MD = 7.00 [95% CI −7.05, 21.05], p = 0.33, I^2^ = 96%) and external rotation (MD = 14.52 [95% CI −13.23, 42.27], p = 0.31, I^2^ = 97%). Most subgroup analyses showed substantial heterogeneity, warranting cautious interpretation (Fig 3).

**Fig 4:**
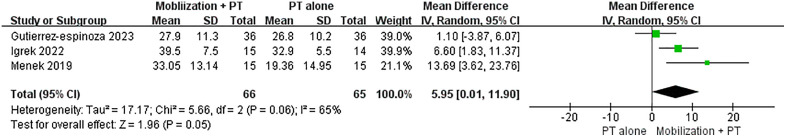
Meta-analysis of disabilities of the arm, shoulder and hand questionnaire (DASH) outcomes between the mobilization plus physical therapy and physical therapy alone groups. SD: standard deviation; CI: confidence interval.

**Fig 5:**
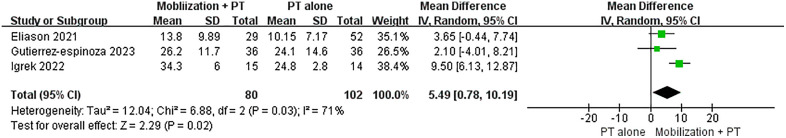
Meta-analysis of Constant-Murley score (CM score) outcomes between the mobilization plus physical therapy and physical therapy alone groups. SD: standard deviation; CI: confidence interval.

A meta-analysis of three studies using the DASH immediately after treatment showed a statistically significant improvement in the add-on group (MD = 5.95 [95% CI 0.01, 11.90], p = 0.05, I^2^ = 65%) (Fig 4). Similarly, a meta-analysis of three studies using the Constant-Murley score demonstrated a significant improvement (MD = 5.49 [95% CI 0.78, 10.19], p = 0.02, I^2^ = 71%) (Fig 5).

#### Comparisons of mobilization plus physical therapy and exercise plus physical therapy.

A between-group analysis was performed in three studies [23,35,36] comparing mobilization plus specific physical therapy with exercise therapy plus the same physical therapy. For simplicity, we refer to the group receiving mobilization in addition to physical therapy as the mobilization group and the group receiving exercise therapy plus physical therapy as the exercise group. Among these studies, Satpute et al. [36] reported greater improvements in the mobilization group across multiple outcomes, including pain-free HBB, VAS, PROM, and SPADI, whereas Aytar et al. [23] and Pekgöz et al. [35] found no statistically significant differences between groups across various functional and patient-reported outcomes.

**Fig 6:**
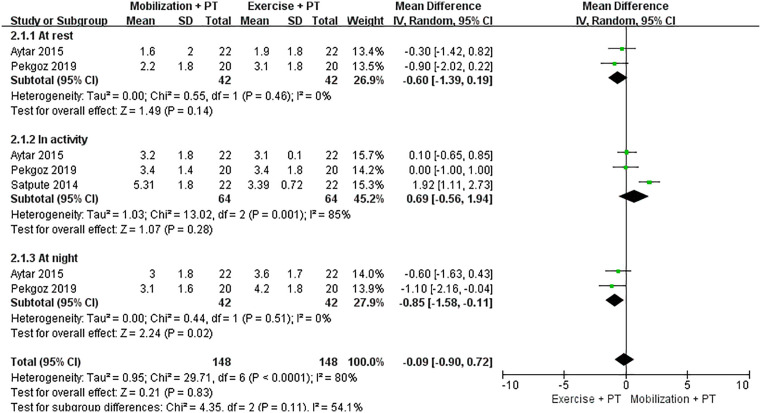
Meta-analysis of visual analog scale (VAS) outcomes between the mobilization plus physical therapy and exercise plus physical therapy groups. SD: standard deviation; CI: confidence interval.

A meta-analysis of three studies using VAS immediately after treatment showed no statistically significant difference between the mobilization and exercise groups (MD = −0.09 [95% CI −0.90, 0.72], p = 0.83, I^2^ = 80%), with substantial heterogeneity. Subgroup analyses by time point showed no significant differences at rest (I^2^ = 0%) or during activity (I^2^ = 85%), while a small but statistically significant effect favoring exercise therapy was observed at night (MD = −0.85 [95% CI −1.58, −0.11], p = 0.02, I^2^ = 0%) (Fig 6).

**Fig 7:**
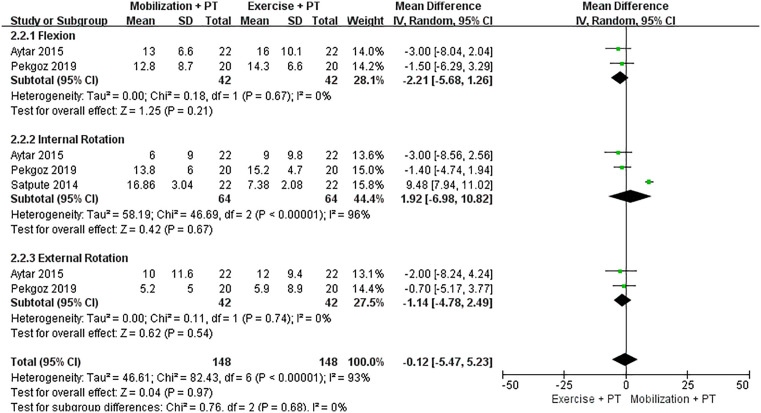
Meta-analysis of range of motion (ROM) outcomes between the mobilization plus physical therapy and exercise plus physical therapy groups. SD: standard deviation; CI: confidence interval.

A meta-analysis of three studies using ROM immediately after treatment also showed no statistically significant difference between groups (MD = 0.29 [95% CI −4.34, 4.91], p = 0.90, I^2^ = 92%). Subgroup analyses by movement direction similarly revealed no significant differences in flexion (I^2^ = 0%) or external rotation (I^2^ = 0%), whereas internal rotation showed high heterogeneity (I^2^ = 96%) without a significant treatment effect. These findings should be interpreted with caution due to the observed heterogeneity (Fig 7).

#### Comparisons of mobilization and sham mobilization.

A between-group analysis was performed in three studies comparing mobilization with sham mobilization [24,26,38]. Beaudreuil et al. [24] and Delgado-Gil et al. [26] reported that the mobilization group was significantly more effective than the sham mobilization group across all assessed outcomes, whereas Surenkok et al. [38] found no significant difference in VAS. However, in that study, mobilization showed greater improvements in AROM, scapular UR, and the Constant shoulder score.

A meta-analysis of two studies using the VAS immediately after treatment demonstrated a statistically significant reduction in pain in the mobilization group (MD = 0.67 [95% CI 0.15, 1.19], p = 0.01, I^2^ = 0%). Subgroup analyses by time point showed no statistically significant differences at rest (I^2^ = 4%) or during activity (I^2^ = 35%), with generally low heterogeneity. One study [38] was labeled as “not estimable” in part of the analysis due to insufficient variance information.

**Fig 8:**
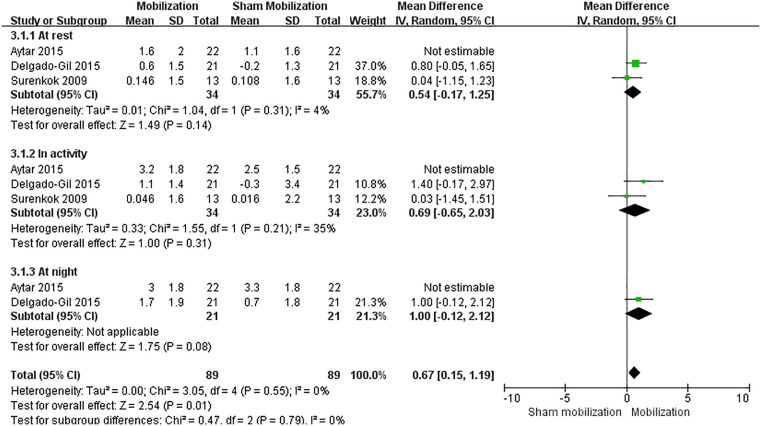
Meta-analysis of visual analog scale (VAS) outcomes between the mobilization and sham mobilization groups. SD: standard deviation; CI: confidence interval.

**Fig 9:**
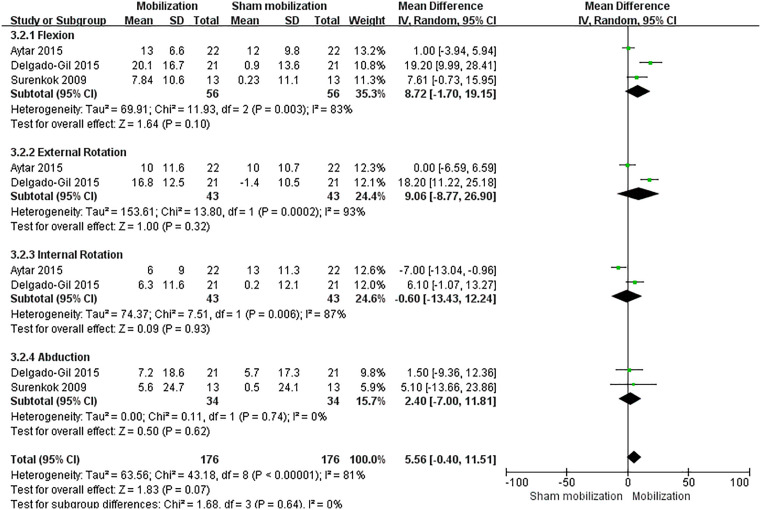
Meta-analysis of active range of motion (AROM) outcomes between the mobilization and sham mobilization groups. SD: standard deviation; CI: confidence interval.

A meta-analysis of two studies using AROM immediately after treatment showed a statistically significant improvement in the mobilization group (MD = 8.56 [95% CI 2.31, 14.82], p = 0.007, I^2^ = 77%). Subgroup analyses by movement demonstrated a significant effect in flexion (I^2^ = 70%), whereas no statistically significant difference was observed in abduction (I^2^ = 0%) (Figs 8, 9).

#### Comparison between mobilization groups.

In a review of two studies that performed between-group analyses of mobilization, Cook et al. [25] compared a group that received mobilization and exercise treatment for both the neck and shoulder with a group that received the same treatment for the shoulder only. They found no significant difference between the two groups in the QuickDASH, NPRS, or PASS. Kachingwe et al. [30] compared the effects of mobilization and the MWM technique on the glenohumeral joint and found no significant differences between the two groups in the VAS, AROM, and SPADI.

#### Comparison of mobilization and other treatments.

Aytar et al. [23] analyzed the two groups that added mobilization and sham mobilization to physical therapy in the QuickDASH, VAS, AROM, and participant satisfaction indicators. They found no statistically significant differences between the two groups. İğrek and Çolak [29] compared the exercise therapy plus mobilization group with the PNF plus mobilization group at 2, 4, and 16 weeks in the VAS, DASH, Constant-Murley score, AROM, and muscle strength. No significant difference was noted between the two groups, except for a statistically significant effect in the mobilization group when compared with that in the PNF group in the flexion of the AROM at 4 weeks.

Park et al. [34] compared the mobilization alone group with the exercise therapy alone group. They reported that the mobilization group was more effective in the thoracic kyphosis angle, muscle tone, PROM, and SPADI; however, no significant difference was found between the two groups in muscle stiffness. Srivastava et al. [37] analyzed the VAS, AROM, and SPADI in the mobilization and exercise therapy and cooling and exercise intervention groups. They found no statistically significant differences between the groups.

#### Comparison by time of treatment intervention.

Guimarães et al. [27] conducted a crossover study comparing a group that underwent MWM for the first four weeks with sham mobilization for the next four weeks, with the reverse group, for a total of eight weeks. The between-group analysis of the two groups in the AROM, isometric peak force, DASH, and SPADI showed that the former group was more effective in the SPADI, and the latter group was more effective in the DASH. No significant difference was found between the two groups in other indicators.

#### Comparison of effects with and those without mobilization after SACS injection.

Kulakli et al. [31] analyzed joint mobilization versus no joint mobilization after SACS injection using the AROM, VAS, and DASH immediately after injection and four weeks later. They reported that the joint mobilization group was statistically more effective than the no joint mobilization group in the range of flexion and abduction and VAS immediately after injection.

#### Adverse reactions.

No adverse events were reported in all 19 studies

#### Duration and number of treatments.

**Fig 10:**
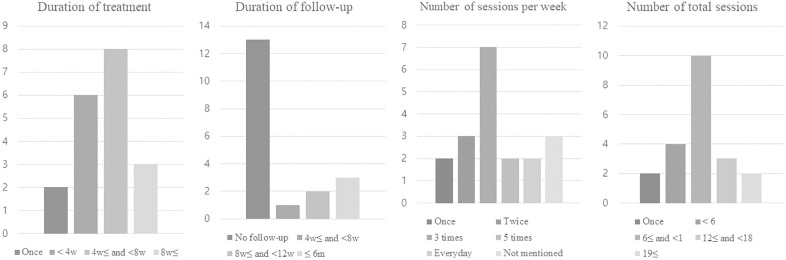
Comparisons of the duration of treatment, duration of follow-up, number of sessions per week, and number of total sessions.

Out of the 19 studies, two received a single treatment. Six studies underwent treatment for less than 4 weeks, eight studies for more than 4 weeks but less than 8 weeks, and three studies for more than 8 weeks. Thirteen studies lacked follow-up, one study with more than 4 weeks but less than 8 weeks after the end of treatment, two studies with more than 8 weeks but less than 12 weeks, and three studies with more than 6 months. Two studies reported one session per week, three studies reported two sessions per week, seven studies reported three sessions per week, two studies reported five sessions per week, and two studies reported daily sessions. Three studies did not specify the frequency of sessions per week. In terms of the total number of sessions, two studies had a single session and four studies had fewer than six sessions, including two one-off studies. Ten studies had between 6 and 12 sessions, three had between 12 and 18 sessions, and two had more than 19 sessions, with the highest number of sessions being 30 (Fig 10).

#### Assessment of risk of bias.

The results of applying the ROB 2.0 tool to assess the risk of bias in the 19 selected articles were as follows. In domain 1 (bias in the randomization process), the majority of the 19 studies were assessed as having a “low risk” of bias. However, due to a lack of randomization description and subsequent concealment, differences in the number of individuals assigned to each group, and practitioner participation in the randomization process, three studies [9,19,37] were assessed as having “some concern” and three studies [30,32,38] were assessed as having a “high risk” of bias. In domain 2 (bias by deviations from intended interventions), four studies [9,30,32,33] were assessed as having “some concern” because some individuals dropped out during the evaluation process, but no clear evidence was found that this occurred in a clinical trial context. In domain 3 (bias by missing outcome data), only one study was assessed as having a “high risk” of bias [9] because appropriate methods were not used to impute missing values.

The characteristic of mobilization as an intervention poses challenges in blinding practitioners and patients, resulting in the majority of studies being classified as having “some concern” and “high risk” of bias in domain 4 (bias by index of outcomes). Five [24,26,30,34,36] of the 19 studies were assessed as having a “low risk” of bias because they clearly stated that blinding was performed properly using a sham treatment, did not disclose the superiority of each treatment, concealed the true purpose of the study, or the patients were blinded to treatments other than their own. The 10 studies [9,10,25,28,29,31–33,37,38] that did not explicitly state that the participants were blinded were assessed as having a “high risk” of bias. Four studies [19,23,27,35] that stated that participants were blinded were assessed as having “some concern,” given the possibility that knowledge of the intervention may have influenced the assessment of participants using measures such as the VAS and SPADI.

**Fig 11:**
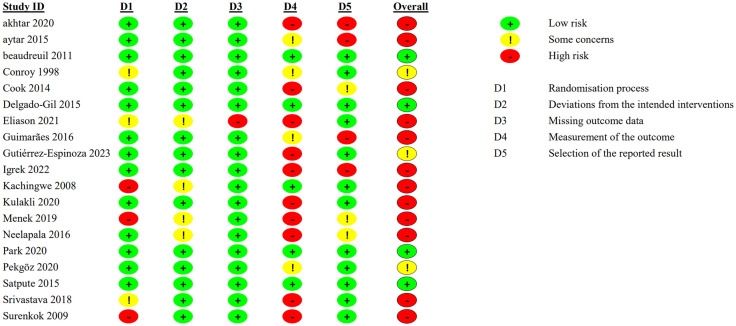
Risk of bias summary. (+) = low risk of bias; (?) = unclear risk of bias; (-) = high risk of bias.

In domain 5 (bias in the selection of reported results), three studies [25,32,33] that did not clearly state that assessments were analyzed before blinding to intervention outcomes were assessed as having “some concern.” Four studies [10,23,27,29] that used multiple appropriate methods were utilized to measure and analyze outcomes, but only some of them were selectively reported and assessed as having a “high risk” of bias. Taken together, we ultimately determined that 4, 3, and 12 studies were assessed as having a “low risk,” “some concern,” and “high risk” of bias, respectively (Fig 11).

## Discussion

### Characteristics of included studies

All 19 studies included in the final analysis incorporated mobilization as an intervention; however, each study used a slightly different method of mobilization. The concept of mobilization in this study included various techniques, including neuromobilization, soft tissue mobilization, joint mobilization, MWM, and dynamic humeral centering (DHC), to mobilize the shoulder joint and its surrounding structures. Among the studies, one study included neuromobilization, 18 involved joint mobilization, 17 applied mobilization to the shoulder joint, while one study focused on mobilization in the thoracic spine, and another study applied mobilization to the cervical spine. Of the studies focusing on shoulder joint mobilization, eight specified a specific technique; one study used DHC and seven used MWM.

Twelve of the nineteen articles compared physical therapy with mobilization. Physical therapy included GE, HE, SWD, US, TENS, STM, NMES, HP, and cooling. Exercise therapy (GE and HE) was included in all 12 studies. Four studies used sham mobilization as a comparison group. One of them added the same physical therapy to both the mobilization and sham groups, and three compared mobilization with no physical therapy. In addition, one study compared mobilization with exercise therapy and two studies used no treatment as a control group.

Most exercise treatments presented in these studies consisted of two phases: stretching and strengthening. Seven studies utilized tools such as elastic bands and wands, including TheraBand. Both stretching and strengthening exercises were based on joint movements such as abduction, adduction, flexion, extension, external rotation, and internal rotation of the shoulder joint. Other studies added retraction, protraction, supination, pronation, raising, lowering, and posterior capsular stretches. The muscles targeted by the strengthening exercises included the rotator cuff (infraspinatus, teres minor, and subscapularis), serratus anterior, pectoralis minor, latissimus dorsi, biceps brachii, deltoid, trapezius, thoracic spinal extensors, and trunk extensors. Additionally, the Codman exercise (pendulum exercise) technique is used to facilitate movement in the scapulothoracic joint without causing damage to recently injured or repaired tissues [39].

Of the four studies that used sham mobilization, three adopted a method identical to conventional mobilization but changed the hand’s position and applied no or minimal pressure. Another study performed mobilization with mild resistance within the pain-free range, describing it as “ineffective for shoulder impingement” rather than using the direct term “sham.” [24] The three aforementioned studies employed techniques such as grasping the pectoralis major instead of the humeral head, targeting the top of the shoulder instead of the scapula, and focusing on the scapular surface and skin instead of the edges of the scapula, and applying minimal force to the movement. Despite these efforts, the aforementioned methods encounter challenges related to force control and blinding. Patient can feel the applied pressure, and varying hand positions can result in unwanted effects when greater pressure is applied.

With regards to outcome indices, the DASH questionnaire is used to measure upper-extremity function, covering a wide range of physical impairments and symptoms, to evaluate the effect of upper-extremity disability on an individual’s daily activities. It consists of 30 items that measure physical symptoms and functional impairment on a five-point Likert scale. Scores range from 0 to 100, with higher scores indicating greater upper-extremity disability and impairment. The DASH has been previously used to assess patients with SIS and has been determined to be reliable and responsive [27]. The QuickDASH uses 11 items instead of 30, enhancing the test’s convenience and usability [23]. The SPADI is an assessment index for shoulder pain and disability, consisting of two subscales. Each subscale uses VASs ranging from 0 to 10. A score of 0 in both pain and disability categories indicates the absence of pain or disability, and a score of 10 indicates severe pain or disability. Higher scores reflect greater severity of pain or disability [27,34]. The Constant–Murley score is a functional assessment with a 100-point scoring system. It consists of four subscores, namely, pain, activities of daily living, AROM, and muscle strength.

With regards to the duration and number of treatments, 19 studies were analyzed using the following criteria: total treatment duration, follow-up duration, number of treatment sessions per week, and the total number of sessions, resulting in a wide range of distributions. To summarize the results, mobilization for SIS tended to be performed for more than four but less than eight weeks, with no follow-up, at a frequency of three sessions per week, totaling six to 12 sessions.

In the risk of bias assessment, four studies were finally determined as “low risk,” three as “some concern” and 12 as “high risk.” This suggests that the results of many studies may have been interfered with by bias, highlighting the need for further clarification of randomization sequences and methods, handling of dropouts and missing data, and reporting of multiple data in future studies. It also suggests that new methods of blinding, such as not informing participants of the ultimate purpose of the study, should be introduced in situations where it is difficult to blind participants in practice.

### Summary of findings

In this study, a meta-analysis was conducted to determine whether mobilization improved the clinical symptoms of SIS. All 19 studies underwent between-group analyses, and 17 of them conducted statistical comparisons between the mobilization and no-mobilization groups. The type of intervention varied across studies, necessitating a preliminary categorization for comparison. Categorization was carried out using intervention methods, control groups, and indices. The clinical heterogeneity observed across the included studies—such as variations in manual therapy techniques (e.g., neural mobilization, soft tissue mobilization, MWM), types of concomitant interventions such as exercise therapy, characteristics of control groups, and treatment dosage like frequency and duration may have contributed to the statistical heterogeneity. Therefore, the pooled results should be interpreted with caution. A meta-analysis was performed on 11 out of the 19 studies, and comparisons were made based on the aforementioned categorization.

The results showed that the addition of mobilization to physical therapy significantly reduced the VAS scores and improved the AROM, DASH, and Constant-Murley scores compared with conventional physical therapy alone. In the AROM subgroup, mobilization was effective in the main painful movements of flexion, internal rotation, and external rotation. However, more research is warranted to prove the effectiveness in extension and external rotation.

We also found a significantly decreased VAS score and partial AROM improvement in the mobilization group compared with the sham mobilization group. However, the lack of significant differences in each VAS subgroup can be attributed to the fact that one [38] of the two analyzed studies included only one mobilization session, which may not have been sufficiently effective. In the AROM subgroup, we observed no significant difference between abduction and flexion, which may be attributed to the mobilization technique used in Delgado-gil et al.’s study, where it was directly combined with flexion [26]. In addition, in the between-group analysis, the mobilization group was significantly more likely to improve the Constant-Murley score [24,38] and Scapular UR [38] (p < 0.05).

VAS and AROM analyses showed no significant difference in effectiveness between the physiotherapy plus mobilization and physiotherapy plus exercise groups. This result can be attributed to the fact that the mechanism of both treatments is the same, that is, to create movement in the tissues, muscles, and joints. The difference between the two interventions is that mobilization is a passive movement applied to the shoulder joint by a therapist, whereas exercise therapy is an active movement applied to the shoulder joint by the patients themselves. Therefore, based on the patient’s main symptoms during shoulder rehabilitation, selecting mobilization may be appropriate if the main problem is soft tissue inflexibility and exercise therapy if the main problem is muscle strength [40]. In the between-group analysis, no significant difference was found in effectiveness between the two groups in most indicators, including the PROM, DASH, and SPADI (p > 0.05).

No adverse events were reported in the included studies. However, the results of this meta-analysis should be interpreted with caution due to substantial statistical heterogeneity, largely attributable to clinical diversity among studies. High I^2^ values (often exceeding 90%) indicate considerable variability in treatment effects, suggesting that the benefits of mobilization are not consistent across all clinical contexts. This variability likely arises from differences in mobilization techniques, treatment dosage, co-interventions, control conditions, and patient characteristics. Consequently, the findings are not universally generalizable to all patients with shoulder impingement syndrome, and clinical application should be individualized. The heterogeneity also limits the precision of pooled estimates, reflecting the context-dependent nature of mobilization in practice. Despite these limitations, mobilization demonstrated overall beneficial effects across most outcome measures [14].

Beyond statistical significance, the clinical meaningfulness of the observed effects should be considered. For example, the magnitude of pain reduction observed in this study may approach commonly cited minimal clinically important difference (MCID) thresholds for shoulder pain. However, interpretation should remain cautious, as outcome measures (e.g., VAS 0–10 vs. 0–100) and study contexts varied across trials. Furthermore, the high heterogeneity limits the ability to draw definitive conclusions regarding the clinical relevance of these pooled estimates.

#### Clinical implications.

From a clinical perspective, joint mobilization can be considered an adjunctive intervention for patients with shoulder impingement syndrome (SIS), particularly for those presenting with limited range of motion, pain-related movement restriction, or difficulty engaging in active exercise-based rehabilitation. It may facilitate early pain reduction and improve joint mobility, thereby promoting participation in subsequent active therapy. However, given the heterogeneity in intervention protocols and patient characteristics across studies, its application should be individualized according to the patient’s clinical presentation and treatment context.

This systematic review provides several implications for clinical practice. First, joint mobilization appears to be a safe and effective adjunct to standard care and may be integrated with exercise therapy to enhance pain relief and functional recovery. Second, treatment selection should be guided by the patient’s primary impairment. Mobilization may be more appropriate as an initial intervention for patients with acute pain or substantial limitations in active movement, whereas structured exercise therapy may be equally effective for those with primary muscle weakness or the capacity for active participation. Third, current evidence does not support the superiority of any specific mobilization technique. Therefore, the choice of technique should be based on clinical expertise and individual patient characteristics until more robust comparative evidence becomes available.

### Limitations

This systematic review was conducted to determine whether the use of mobilization to improve SIS symptoms can achieve significant clinical benefits. However, this study had several limitations. First, this review was limited by the high heterogeneity of intervention methods in the studies included in this review. Since the literature search found only three studies [26,34,38] that included mobilization alone as an intervention, which is a small number. Therefore, we widened the scope of the mobilization group to include a broad range of mobilization techniques. However, this diversity—which also included variations in treatment frequency, duration, and the type of physical therapy used as co-interventions—made a subgroup meta-analysis based on specific technique types unfeasible. The number of studies for any single technique was too small for a meaningful statistical comparison (e.g., only one study for DHC and seven for MWM, which were often combined with other therapies). This limitation ultimately prevents us from drawing conclusions about the comparative effectiveness of different mobilization approaches and makes it difficult to determine the most clinically beneficial technique. Second, the duration of follow-up in each study was inconsistent. While a few studies [9,23,29] with longer follow-up periods were available, the number was too small to allow for statistical analysis between groups. Consequently, we were unable to determine the long-term clinical effects. As this may affect the interpretation of the results, we conducted sub-analyses using the same criteria to minimize heterogeneity. We also considered that different outcome index between studies may make it difficult to synthesise results, so we used methods to standardise different outcome index or to use integrated analysis of the results to address this.

Third, nine of the 19 studies had 20 or fewer patients in each group. This absolute lack of sufficient sample size may result in insufficient statistical power. Small studies can increase random error and affect the reliability of meta-analysis results, thereby complicating the ability to express confidence in their effectiveness in real-world clinical practice. To compensate for this, we set appropriate confidence intervals for our analyses in the meta-analyses. Fourth, the assessment indices used in each study were too diverse to allow for a detailed subgroup analysis. For example, although many studies used AROM as an index, various types of shoulder joint movements were measured, leaving a small number of studies for each movement-specific analysis. Consequently, the numerous subgroup analyses introduce the possibility of multiple testing errors, meaning these results must be interpreted cautiously. Furthermore, the risk of undetected publication bias remains a concern. Finally, the overall strength of our evidence is limited by significant methodological flaws in the included literature. A substantial proportion of the studies (12 out of 19) were assessed as having a “high risk” of bias, primarily due to failures in randomization, reliable blinding, and accurate outcome reporting. Because of this high proportion of flawed studies, we were unable to conduct our planned sensitivity analysis; excluding these 12 studies would have left too few trials in each subgroup to perform meaningful meta-analyses. This limitation strongly underscores the critical need for future RCTs with rigorous methodological designs to confirm these findings.

This fact could have led to an overestimation of the treatment effect, particularly for subjective outcomes like pain scores (VAS). Therefore, despite the positive findings presented here, the results must be interpreted with significant caution, fully considering this potential for bias. In addition, we did not perform a formal GRADE assessment; therefore, the certainty of the evidence was not formally evaluated. This further limits the strength of the conclusions drawn from this study.

### Suggestions for further research

Despite the aforementioned limitations, this study is important because it successfully identified evidence for the clinical efficacy of mobilization in improving the main symptoms of SIS based on an extensive systematic review conducted in three major databases. The scope of the study was broader than that of previous systematic reviews, which only addressed a subset of shoulder symptoms or specific mobilization techniques, and included recently published studies. The study presents the statistical pooling of multiple data through a meta-analysis, which generally provides greater precision [12].

To build a clearer and more reliable rationale for the use of mobilization beyond the limitations of this study, the authors suggest the following. First, the separation of mobilization from other interventions is necessary to obtain a clearer independent treatment effect of mobilization in RCTs. Several studies excluded from the review included various interventions, making it difficult to identify the independent effects of mobilization itself. Therefore, future studies should be designed to either evaluate mobilization as an independent intervention or directly compare specific, well-defined techniques under a standardized protocol. Next, to reduce the risk of bias in the study, future studies must employ rigorous methodological designs. This includes establishing a completely randomized setting and ensuring thorough assessment to verify that the omission or correction of results does not introduce distortion to the conclusions are important. A key challenge, highlighted by the high risk of bias in 10 of the 19 studies we reviewed, is the inherent difficulty of reliable blinding die tp the characteristics of physical therapy. To mitigate this, future research should implement specific strategies, such as concealing the ultimate purpose of the study from subjects or blinding to interventions applied to other groups. Also, to directly address the major limitations identified in this study, large-scale RCTs are required. These trials should compare specific, clearly defined mobilization techniques with a reliable sham intervention, and include long-term follow-up to evaluate the durability of the effects. Critically, ensuring the thorough blinding of outcome assessors is also essential to maintain objectivity. Third, studies with long-term follow-up in large samples should be included to increase the reliability of the evidence for mobilization [34,36]. Finally, although this study successfully synthesized and utilized the broad concept of mobilization, allowing for an assessment of its effectiveness as a category of therapeutic interventions in improving SIS, it did not facilitate detailed comparisons between individual techniques. Consequently, determining the most effective technique in real-world practice remains challenging. Future research should prioritize high-quality randomized controlled trials (RCTs) that directly compare the effectiveness of individual, well-defined mobilization techniques against each other (e.g., MWM versus glenohumeral joint mobilization) and against credible sham controls. This will help identify the most beneficial approach for patients with SIS.

## Conclusion

This systematic review and meta-analysis demonstrated that adding mobilization to physical therapy is superior to physical therapy alone in improving pain and movement limitations, the primary symptoms of SIS, based on indices such as VAS, AROM, DASH, and Constant-Murley scores. Mobilization was also found to be more effective in improving pain and ROM than sham mobilization based on indices such as VAS and AROM. However, no statistically significant difference was found in the improvement of pain and ROM limitation between physical therapy plus mobilization and physical therapy plus exercise therapy. This is probably because both interventions are essentially the same in moving structures, with the differences being passive or active. Therefore, clinically, mobilization may be more advisable when patients have weak muscle strength or limited AROM or when an acute injury prevents active movement. These benefits appear to be primarily short-term and adjunctive in nature. However, the quality of the evidence was not high, owing to the high heterogeneity among the included studies and the inclusion of a large number of studies with a high risk of bias assessment. Future studies that are methodologically sound, include a larger number of subjects, and have long-term follow-up are needed.

## Supporting information

S1 Text S1 File. PRISMA checklist. **S2 File.** Search expressions. **S3 File.**The list of excluded studies with reasons. **S4 File.** The detailed data for the synthesis.
(ZIP)
