# Supplementary material for: Clinical efficacy of joint mobilization for shoulder impingement syndrome: a systematic review and meta-analysis
Source: PLoS One. 2026 Jul 13;21(7):e0352260. doi: 10.1371/journal.pone.0352260 (PMC13362101; doi:10.1371/journal.pone.0352260)
Supplement: S1 Text — S2 File. Search expressions. S3 File.The list of excluded studies with reasons. S4 File. The detailed data for the synthesis. (ZIP) [file pone.0352260.s001.zip › S2 Search Expressions.docx]

**S2 Search Expressions.**

**1.1. PubMed**

“mobilization”[ti] AND (“Shoulder Impingement Syndrome”[Mesh] OR “imping*”[tw] OR “rotator”[tw]) AND ((“Controlled Clinical Trials as Topic”[Mesh] OR “Randomized Controlled Trials as Topic”[Mesh] OR Randomized Controlled Trial[PT] OR Controlled Clinical Trial[PT] OR Multicenter Study[PT] OR “Clinical Trials as topic”[Mesh] OR Random-Allocat*[TW] OR randomized[TW] OR randomised[TW] OR ((Double*[TW] OR single*[TW] OR treb*[TW] OR tripl*[TW]) AND (Blind*[TW] OR mask*[TW])) OR controlled-clinical-trial*[TW] OR controlled-trial*[TW] OR placebo*[TW] OR randomly*[TW]) NOT (Case Reports[PT] OR Letter[PT] OR meta-analyses*[TW] OR metaanalyses*[TW] OR "Meta-Analysis" [PT] OR case-report*[TW] OR Letter*[TI] OR “Systematic Review”[PT] OR Systematic-Review*[TI])

**1.2. Embase**

(“joint mobilization”/exp OR “mobilization”:ti) AND (“shoulder impingement syndrome”/exp OR 'imping*':ab,kw,ti) AND ((“randomized controlled trial”/exp OR [randomized controlled trial]/lim OR “controlled clinical trial”/de OR “multicenter study”/exp OR “randomization”/exp OR (Random-Allocat* OR randomized OR randomized OR controlled-clinical-trial* OR controlled-trial* OR placebo* OR randomly*):ab,ti,kw OR ((Double* OR single* OR treb* OR tripl*) NEAR/3 (Blind* OR mask*)):ab,ti,kw) NOT ([conference abstract]/lim OR [conference paper]/lim OR [conference review]/lim OR [data papers]/lim OR [editorial]/lim OR [erratum]/lim OR [letter]/lim OR [note]/lim OR [review]/lim))

**1.3. The Cochrane Library**

((“mobilization”):ti) AND (mh“Shoulder Impingement Syndrome” OR (“imping*” OR “rotator”):ti,ab,kw) AND (“random*”):ti
