# Supplementary material for: Clinical efficacy of joint mobilization for shoulder impingement syndrome: a systematic review and meta-analysis
Source: PLoS One. 2026 Jul 13;21(7):e0352260. doi: 10.1371/journal.pone.0352260 (PMC13362101; doi:10.1371/journal.pone.0352260)
Supplement: S1 Text — S2 File. Search expressions. S3 File.The list of excluded studies with reasons. S4 File. The detailed data for the synthesis. (ZIP) [file pone.0352260.s001.zip › S3 The list of excluded studies with reasons.docx]

**S2 Table. The list of excluded studies with reasons**

|  | Articles Title | Author | Year | Exclusion reasons | Data extractor | Date of Extraction |
| --- | --- | --- | --- | --- | --- | --- |
| 1 | Effect of Low Level Laser with and without Shoulder Mobilization on Shoulder Function in Patients with Shoulder Impingement Syndrome | Alsharief | 2022 | Unrelated article | GL and WH | July 2023 |
| 2 | Spinal Manipulation and Electrical Dry Needling in Patients With Subacromial Pain Syndrome: A Multicenter Randomized Clinical Trial | Dunning | 2021 | Unrelated article | GL and WH | July 2023 |
| 3 | Does the outcome of diagnostic ultrasound influence the treatment modalities and recovery in patients with shoulder pain in physiotherapy practice? Results from a prospective cohort study | Karel | 2019 | Unrelated article | GL and WH | July 2023 |
| 4 | Subscapularis tenotomy versus lesser tuberosity osteotomy during total shoulder arthroplasty for primary osteoarthritis: a prospective, randomized controlled trial | Levine | 2019 | Unrelated article | GL and WH | July 2023 |
| 5 | The effects of thoracic spine manipulation in subjects with signs of rotator cuff tendinopathy | Muth | 2012 | Unrelated article | GL and WH | July 2023 |
| 6 | What is the Rate of Response to Nonoperative Treatment for Hip-Related Pain? A Systematic Review With Meta-analysis | Probst | 2023 | Unrelated article | GL and WH | July 2023 |
| 7 | Comparison of specific and non-specific treatment approaches for individuals with posterior capsule tightness and shoulder impingement symptoms: A randomized controlled trial | Rosa | 2021 | Unrelated article | GL and WH | July 2023 |
| 8 | Re: the addition of cervical unilateral posterior-anterior mobilisation in the treatment of patients with shoulder impingement syndrome: a randomized clinical trial | A Garg | 2015 | Unrelated article | GL and WH | July 2023 |
| 9 | No difference in long-term outcome between open and arthroscopic rotator cuff repair: a prospective, randomized study | A Hasler | 2020 | Unrelated article | GL and WH | July 2023 |
| 10 | Combined Hip Abductor and External Rotator Strengthening and Hip Internal Rotator Stretching Improves Pain and Function in Patients With Patellofemoral Pain Syndrome: a Randomized Controlled Trial With Crossover Design | A Jellad | 2021 | Unrelated article | GL and WH | July 2023 |
| 11 | Mobilization after superior rotator cuff repair: sling versus no-sling. A randomized prospective study | A Ladermann | 2019 | Unrelated article | GL and WH | July 2023 |
| 12 | Postoperative mobilization after rotator cuff repair: sling versus nothing: a randomized prospective study | A Laedermann | 2019 | Unrelated article | GL and WH | July 2023 |
| 13 | Management of adults with primary frozen shoulder in secondary care (UK FROST): a multicentre, pragmatic, three-arm, superiority randomised clinical trial | A Rangan | 2020 | Unrelated article | GL and WH | July 2023 |
| 14 | Response to Letter to the Editor: the addition of cervical Unilateral Posterior Anterior Mobilization (UPA) in the treatment of patients with shoulder impingement syndrome: a randomized clinical trial | C Cook | 2015 | Unrelated article | GL and WH | July 2023 |
| 15 | Rehabilitation following rotator cuff repair: multi-centre pilot and feasibility randomised controlled trial (RaCeR) | C Littlewood | 2021 | Unrelated article | GL and WH | July 2023 |
| 16 | Dynamic cerebral autoregulation during early orthostatic exercise in patients with severe traumatic brain injury: further exploratory analyses from a randomized clinical feasibility trial | Cg Riberholt | 2021 | Unrelated article | GL and WH | July 2023 |
| 17 | Mobilising patients with severe acquired brain injury in intensive care (MAWERIC) – Protocol for a randomised cross-over trial | Cg Riberholt | 2022 | Unrelated article | GL and WH | July 2023 |
| 18 | Effects of robot (SUBAR)-assisted gait training in patients with chronic stroke Randomized controlled trial | Cj Kang | 2021 | Unrelated article | GL and WH | July 2023 |
| 19 | Early mobilisation following mini-open rotator cuff repair: a randomised control trial | Dm Sheps | 2015 | Unrelated article | GL and WH | July 2023 |
| 20 | Manual hyperinflation partly prevents reductions of functional residual capacity in cardiac surgical patients: a randomized controlled trial | F Paulus | 2011 | Unrelated article | GL and WH | July 2023 |
| 21 | Effects of Bobath treatment and specific mobilizations on gait in stroke patients: a randomized clinical trial | G Grozdek Čovčić | 2022 | Unrelated article | GL and WH | July 2023 |
| 22 | Early mobilisation versus plaster immobilisation of simple elbow dislocations: results of the FuncSiE multicentre randomised clinical trial | Gi Iordens | 2017 | Unrelated article | GL and WH | July 2023 |
| 23 | Effect of weight-bearing wrist movement with carpal-stabilizing taping on pain and range of motion in subjects with dorsal wrist pain: a randomized controlled trial | Gs Kim | 2020 | Unrelated article | GL and WH | July 2023 |
| 24 | Effectiveness of the end-range/scapular mobilization approach in a subgroup of subjects with frozen shoulder syndrome: a randomized control trial | H Huang | 2011 | Unrelated article | GL and WH | July 2023 |
| 25 | A randomized phase 3 study on the effect of thalidomide combined with adriamycin, dexamethasone, and high-dose melphalan, followed by thalidomide maintenance in patients with multiple myeloma | Hm Lokhorst | 2020 | Unrelated article | GL and WH | July 2023 |
| 26 | Immobilisation versus immediate mobilisation after intrauterine insemination: randomised controlled trial | Im Custers | 2009 | Unrelated article | GL and WH | July 2023 |
| 27 | A multicenter randomised controlled trial assessing the effectiveness of immobilisation versus immediate mobilisation after intrauterine insemination | Im Custers | 2008 | Unrelated article | GL and WH | July 2023 |
| 28 | Effects of robot-(Morning Walk®) assisted gait training for patients after stroke: a randomized controlled trial | J Kim | 2019 | Unrelated article | GL and WH | July 2023 |
| 29 | Postoperative Mobilization After Superior Rotator Cuff Repair: sling Versus No Sling: a Randomized Prospective Study | J Tirefort | 2019 | Unrelated article | GL and WH | July 2023 |
| 30 | Effects of trunk stabilization training robot on postural control and gait in patients with chronic stroke: a randomized controlled trial | Jh Min | 2020 | Unrelated article | GL and WH | July 2023 |
| 31 | Physical behavior and function early after hip fracture surgery in patients receiving comprehensive geriatric care or orthopedic care--a randomized controlled trial | K Taraldsen | 2014 | Unrelated article | GL and WH | July 2023 |
| 32 | Mobilization After Superior Rotator Cuff Repair: sling Versus No-Sling. A Randomized Prospective Study | A. Ladermann | 2019 | Unrelated article | GL and WH | July 2023 |
| 33 | Sustained outcomes following movement pattern training or strengthening/flexibility among patients with prearthritic hip disorders: results of a pilot multicenter randomized clinical trial | M Harris-Hayes | 2020 | Unrelated article | GL and WH | July 2023 |
| 34 | A randomized phase 2 trial of a preparative regimen of bortezomib, high-dose melphalan, arsenic trioxide, and ascorbic acid | M Sharma | 2012 | Unrelated article | GL and WH | July 2023 |
| 35 | Effects of early manual therapy on functional outcomes after volar plating of distal radius fractures: a randomized controlled trial | M Tomruk | 2020 | Unrelated article | GL and WH | July 2023 |
| 36 | Autonomic response to early head-up tilt in patients with severe traumatic brain injury: analysis from a randomized feasibility trial | Md Schultz | 2023 | Unrelated article | GL and WH | July 2023 |
| 37 | Short term effect of spinal mobilization with movement (MWM) on pulmonary functions in nonsmokers with thoracic hyperkyphosis: a randomized single-blinded controlled trial | Mh El Gendy | 2023 | Unrelated article | GL and WH | July 2023 |
| 38 | Study Protocol: randomised controlled trial to investigate the functional significance of marginal riboflavin status in young women in the UK (RIBOFEM) | Mh Hill | 2009 | Unrelated article | GL and WH | July 2023 |
| 39 | Immediate combined effect of gastrocnemius stretching and sustained talocrural joint mobilization in individuals with limited ankle dorsiflexion: a randomized controlled trial | Mh Kang | 2015 | Unrelated article | GL and WH | July 2023 |
| 40 | End-to-side versus side-to-side anastomosis after laparoscopic right hemicolectomy for colon cancer: short-term outcomes of a randomized controlled trial | Mh Kim | 2020 | Unrelated article | GL and WH | July 2023 |
| 41 | Objective recovery time with end-to-side versus side-to-side anastomosis after laparoscopic right hemicolectomy for colon cancer: a randomized controlled trial | Mh Kim | 2022 | Unrelated article | GL and WH | July 2023 |
| 42 | Racial Differences in the Effect of Granulocyte Macrophage Colony-Stimulating Factor on Improved Walking Distance in Peripheral Artery Disease: the PROPEL Randomized Clinical Trial | Mm McDermott | 2019 | Unrelated article | GL and WH | July 2023 |
| 43 | Effects of spinal manipulation or mobilization as an adjunct to neurodynamic mobilization for lumbar disc herniation with radiculopathy: a randomized clinical trial | Ms Danazumi | 2021 | Unrelated article | GL and WH | July 2023 |
| 44 | Does an Early and Aggressive Combined Wrapping and Dangling Procedure Affect the Clinical Outcome of Lower Extremity Free Flaps?-A Randomized Controlled Prospective Study Using Microdialysis Monitoring | N Neubert | 2016 | Unrelated article | GL and WH | July 2023 |
| 45 | No Sling After Rotator Cuff Repair: balancing Short-Term Benefit with Long-Term Health of Shoulder: commentary on an article by Jérôme Tirefort, MD, et al.: "Postoperative Mobilization After Superior Rotator Cuff Repair: sling Versus No Sling. A Randomized Prospective Study" | A. S. Neviaser | 2019 | Unrelated article | GL and WH | July 2023 |
| 46 | Operative treatment of dislocated midshaft clavicular fractures: plate or intramedullary nail fixation? A randomized controlled trial | Oa van der Meijden | 2015 | Unrelated article | GL and WH | July 2023 |
| 47 | The Ruptured Achilles Tendon Elongates for 6 Months After Surgical Repair Regardless of Early or Late Weightbearing in Combination With Ankle Mobilization: a Randomized Clinical Trial | P Eliasson | 2018 | Unrelated article | GL and WH | July 2023 |
| 48 | Adductor canal block versus femoral nerve block and quadriceps strength: a randomized, double-blind, placebo-controlled, crossover study in healthy volunteers | P Jaeger | 2013 | Unrelated article | GL and WH | July 2023 |
| 49 | Effect of the adductor-canal-blockade and femoral nerve block on muscle strength and mobilization in healthy volunteers: a randomized study | P Jger | 2012 | Unrelated article | GL and WH | July 2023 |
| 50 | A randomized controlled trial comparing subscapularis tenotomy with peel in anatomic shoulder arthroplasty | P Lapner | 2020 | Unrelated article | GL and WH | July 2023 |
| 51 | A randomised trial comparing two rehabilitation approaches following reverse total shoulder arthroplasty | Pk Edwards | 2021 | Unrelated article | GL and WH | July 2023 |
| 52 | The inclusion of mobilisation with movement to a standard exercise programme for patients with rotator cuff related pain: a randomised, placebo-controlled protocol trial | R Baeske | 2020 | Unrelated article | GL and WH | July 2023 |
| 53 | Comments on effects of routine physiotherapy with and without neuromobilization in the management of internal shoulder impingement syndrome: a randomized controlled trial | Rr Chughtai | 2021 | Unrelated article | GL and WH | July 2023 |
| 54 | A randomized phase II study of standard-dose versus high-dose rituximab with BEAM in autologous stem cell transplantation for relapsed aggressive B-cell non-hodgkin lymphomas: long term results | Sa Srour | 2017 | Unrelated article | GL and WH | July 2023 |
| 55 | Effect of specific exercise strategy on need for surgery in patients with subacromial impingement syndrome: randomised controlled study | T Holmgren | 2014 | Unrelated article | GL and WH | July 2023 |
| 56 | Economic analysis of a randomized clinical trial to compare filgrastim-mobilized peripheral-blood progenitor-cell transplantation and autologous bone marrow transplantation in patients with Hodgkin's and non-Hodgkin's lymphoma | Tj Smith | 1997 | Unrelated article | GL and WH | July 2023 |
| 57 | Tumour extraction from vertical periumbilical wound vs transverse left iliac fossa wound in laparoscopic anterior resections: a randomized controlled trial | Ws Tan | 2013 | Unrelated article | GL and WH | July 2023 |
| 58 | Motixafortide and G-CSF to mobilize hematopoietic stem cells for autologous transplantation in multiple myeloma: a randomized phase 3 trial | Zd Crees | 2023 | Unrelated article | GL and WH | July 2023 |
| 59 | Mobilization with movement applied to the elbow affects shoulder range of movement in subjects with lateral epicondylalgia | Abbott | 2001 | Unrelated article | GL and WH | July 2023 |
| 60 | The Effectiveness of Aspirin for Venous Thromboembolism Prophylaxis for Patients Undergoing Arthroscopic Rotator Cuff Repair | Alyea | 2019 | Unrelated article | GL and WH | July 2023 |
| 61 | Ultrasound-determined healing rates with subscapularis tenotomy versus peel after anatomic shoulder arthroplasty | L. P. Baisi | 2023 | Unrelated article | GL and WH | July 2023 |
| 62 | Platelet-rich plasma supplementation in arthroscopic repair of full-thickness rotator cuff tears: a randomized clinical trial | D'Ambrosi | 2016 | Unrelated article | GL and WH | July 2023 |
| 63 | Passive mobilization after arthroscopic rotator cuff repair is not detrimental in the early postoperative period | De Roo | 2015 | Unrelated article | GL and WH | July 2023 |
| 64 | Effects of diaphragm muscle treatment in shoulder pain and mobility in subjects with rotator cuff injuries: A dataset derived from a pilot clinical trial | Fernández-López | 2021 | Unrelated article | GL and WH | July 2023 |
| 65 | Tissue characteristics in tendon-to-bone healing change after rotator cuff repair using botulinumneurotoxin A for temporary paralysis of the supraspinatus muscle in rats | Ficklscherer | 2014 | Unrelated article | GL and WH | July 2023 |
| 66 | Double-Row Repair Lowers the Retear Risk After Accelerated Rehabilitation | Franceschi | 2016 | Unrelated article | GL and WH | July 2023 |
| 67 | Effects of Different Orthoses on Neuromuscular Activity of Superficial and Deep Shoulder Muscles during Activities of Daily Living and Physiotherapeutic Exercises in Healthy Participants | Grim | 2022 | Unrelated article | GL and WH | July 2023 |
| 68 | Does patient-controlled continuous interscalene block improve early functional rehabilitation after open shoulder surgery? | Hofmann-Kiefer | 2008 | Unrelated article | GL and WH | July 2023 |
| 69 | Immobilization After Rotator Cuff Repair: What Evidence Do We Have Now? | J. E. Hsu | 2016 | Unrelated article | GL and WH | July 2023 |
| 70 | Suprascapular Nerve Block Is an Effective Pain Control Method in Patients Undergoing Arthroscopic Rotator Cuff Repair: A Randomized Controlled Trial | J. Y. Kim | 2021 | Unrelated article | GL and WH | July 2023 |
| 71 | The Lesser Tuberosity Osteotomy Exposure for Total Shoulder Arthroplasty | Knudsen | 2021 | Unrelated article | GL and WH | July 2023 |
| 72 | A randomized controlled trial comparing subscapularis tenotomy with peel in anatomic shoulder arthroplasty | Lapner | 2020 | Unrelated article | GL and WH | July 2023 |
| 73 | Spine stabilization is a risk factor for the development of pelvic iliac vein lesions | Pappas | 2020 | Unrelated article | GL and WH | July 2023 |
| 74 | The immediate effects of passive hip joint mobilization on hip abductor/external rotator muscle strength in patients with anterior knee pain and impaired hip function. A randomized, placebo-controlled crossover trial | Pfluegler | 2021 | Unrelated article | GL and WH | July 2023 |
| 75 | Effect of rotator cuff strengthening as an adjunct to standard care in subjects with adhesive capsulitis: A randomized controlled trial | Rawat | 2017 | Unrelated article | GL and WH | July 2023 |
| 76 | The effect of granulocyte-colony stimulating factor on rotator cuff healing after injury and repair | Ross | 2015 | Unrelated article | GL and WH | July 2023 |
| 77 | Early Active Motion Versus Sling Immobilization After Arthroscopic Rotator Cuff Repair: A Randomized Controlled Trial | Sheps | 2019 | Unrelated article | GL and WH | July 2023 |
| 78 | Rehabilitation of An Analgesic Bracelet Based on Wrist-Ankle Acupuncture in Patients with Rotator Cuff Injury: A Randomized Trial | W. Song | 2021 | Unrelated article | GL and WH | July 2023 |
| 79 | Imaging of osteochondral lesions of the talus | Stroud | 2000 | Unrelated article | GL and WH | July 2023 |
| 80 | Changes in glenohumeral translation, electromyographic activity, and pressure-pain thresholds following sustained or oscillatory mobilizations in stiff and healthy shoulders: Results of a randomized, controlled laboratory trial | Swanson | 2020 | Unrelated article | GL and WH | July 2023 |
| 81 | Postoperative Mobilization After Superior Rotator Cuff Repair: Sling Versus No Sling: A Randomized Prospective Study | Tirefort | 2019 | Unrelated article | GL and WH | July 2023 |
| 82 | A Preliminary Randomized Clinical Trial on the Effect of Cervicothoracic Manipulation Plus Supervised Exercises vs a Home Exercise Program for the Treatment of Shoulder Impingement | Vinuesa-Montoya | 2017 | Unrelated article | GL and WH | July 2023 |
| 83 | Effectiveness of the end-range mobilization and scapular mobilization approach in a subgroup of subjects with frozen shoulder syndrome: a randomized control trial | J. L. Yang | 2012 | Unrelated article | GL and WH | July 2023 |
| 84 | Delayed early passive motion is harmless to shoulder rotator cuff healing in a rabbit model | S. Zhang | 2013 | Unrelated article | GL and WH | July 2023 |
| 85 | Implementing a training intervention to support caregivers after stroke: a process evaluation examining the initiation and embedding of programme change | D. J. Clarke | 2013 | Unrelated article | GL and WH | July 2023 |
| 86 | Effects of self-stretching with mobilization on shoulder range of motion in individuals with glenohumeral internal rotation deficits: a randomized controlled trial | Mh Kang | 2020 | Unrelated article | GL and WH | July 2023 |
| 87 | **Effects of Stretching and Strengthening Exercises, With and Without Manual Therapy, on Scapular Kinematics, Function, and Pain in Individuals With Shoulder Impingement: A Randomized Controlled Trial** | Camargo | 2015 | Unrelated article | GL and WH | July 2023 |
| 88 | **Effect of manual physiotherapy in homogeneous individuals with subacromial shoulder impingement: A randomized controlled trial** | Land | 2019 | Unrelated article | GL and WH | July 2023 |
| 89 | **Short-term effects of high-intensity laser therapy, manual therapy, and Kinesio taping in patients with subacromial impingement syndrome** | Pekyavas | 2016 | Unrelated article | GL and WH | July 2023 |
| 90 | **Progressive Resistance Exercises plus Manual Therapy Is Effective in Improving Isometric Strength in Overhead Athletes with Shoulder Impingement Syndrome: A Randomized Controlled Trial** | Sharma | 2021 | Unrelated article | GL and WH | July 2023 |
| 91 | **Effects of ischemic compression and instrument-assisted soft tissue mobilization techniques in trigger point therapy in patients with rotator cuff pathology: randomized controlled study** | Aksan | 2022 | Unrelated article | GL and WH | July 2023 |
| 92 | **Real versus Sham Manual Therapy in Addition to Therapeutic Exercise in the Treatment of Non-Specific Shoulder Pain: A Randomized Controlled Trial** | Naranjo-Cinto | 2022 | Unrelated article | GL and WH | July 2023 |
| 93 | The aim of this study is to find out the Effect of Maitland and Mulligan Mobilization on Pain, Range of Motion and functional Disability in patients with Rotator Cuff Syndrome: randomized Clinical Trial | - | - | Not exist article | GL and WH | July 2023 |
| 94 | THE EFFECT OF SPINAL MOBILISATION WITH ARM MOVEMENT AND A MAITLAND MOBILISATION ON RANGE OF MOTION AND MUSCLE ACIVITY IN OVERHEAD ATHLETES WITH SUBACROMIAL IMPINGEMENT SYNDROME - A RANDOMISED CLINICAL TRIAL | - | 2019 | Not exist article | GL and WH | July 2023 |
| 95 | A study of the efficacy of the addition of a ropivacaine infusion to the analgesia provided by pre-emptive ropivacaine and intra-operative parecoxib for peri-operative pain management in adults undergoing elective arthroscopic rotator cuff surgery: a randomised double blind placebo controlled trial | - | 2019 | Not exist article | GL and WH | July 2023 |
| 96 | Efficacy of physiotherapy after hydrodilatation for the painful stiff shoulder: a randomised placebo-controlled trial | - | 2019 | Not exist article | GL and WH | July 2023 |
| 97 | Cost-effectiveness of biceps tenotomy with or without cuff repair in patients with stage 2-3 Goutallier fatty degenerative cuff lesions. A randomized controlled trial | - | 2019 | Not exist article | GL and WH | July 2023 |
| 98 | A pilot double-blind randomised controlled trial comparing two physiotherapy interventions to treat femoroacetabular impingement | - | 2019 | Not exist article | GL and WH | July 2023 |
| 99 | A double-blind randomised controlled trial comparing two physiotherapy interventions to treat hip impingement | - | - | Not exist article | GL and WH | July 2023 |
| 100 | Prospective, Randomized Clinical Trial Comparing Early Versus Delayed Postoperative Mobilization After Arthroscopic Rotator Cuff Tear Reapair | - | - | Not exist article | GL and WH | July 2023 |
| 101 | Effect of dynamic humeral centering on painful active elevation of the arm in subacromial impingement syndrome: a randomized trial | J. Beaudreuil | 2012 | Not exist article | GL and WH | July 2023 |
| 102 | Efficacy of dynamic humeral centering according to Neer test results: a stratified analysis of a randomized-controlled trial | J. Beaudreuil | 2015 | Not randomized controlled trial | GL and WH | July 2023 |
| 103 | The efficacy of manual therapy for rotator cuff tendinopathy: a systematic review and meta-analysis | Desjardins-Charbonneau | 2015 | Not randomized controlled trial | GL and WH | July 2023 |
| 104 | The Effectiveness of Physiotherapy Exercises in Subacromial Impingement Syndrome: A Systematic Review and Meta-Analysis | Hanratty | 2012 | Not randomized controlled trial | GL and WH | July 2023 |
| 105 | The effectiveness of slider and tensioner neural mobilization techniques in the management of upper quadrant pain: A systematic review of randomized controlled trials | Papacharalambous | 2022 | Not randomized controlled trial | GL and WH | July 2023 |
| 106 | Symptom response to mobilization and outcomes in patients with subacromial pain syndrome: A cohort study | Riley | 2021 | Not randomized controlled trial | GL and WH | July 2023 |
| 107 | Treatment of myofascial trigger points in common shoulder disorders by physical therapy: a randomized controlled trial | C Bron | 2007 | Not randomized controlled trial | GL and WH | July 2023 |
| 108 | Protocol for a multi-centre pilot and feasibility randomised controlled trial with a nested qualitative study: rehabilitation following rotator cuff repair (the RaCeR study) | C Littlewood | - | Not randomized controlled trial | GL and WH | July 2023 |
| 109 | Rehabilitation following rotator cuff repair: a nested qualitative study exploring the perceptions and experiences of participants in a randomised controlled trial | G Stephens | 2021 | Not randomized controlled trial | GL and WH | July 2023 |
| 110 | Efficacy and cost-effectiveness of a physiotherapy program for chronic rotator cuff pathology: a protocol for a randomised, double-blind, placebo-controlled trial | K Bennell | 2007 | Not randomized controlled trial | GL and WH | July 2023 |
| 111 | Efficacy of mobilization with movement (MWM) for shoulder conditions: a systematic review and meta-analysis | Satpute | 2022 | Not randomized controlled trial | GL and WH | July 2023 |
| 112 | Early versus delayed mobilization following rotator cuff repair | Bakti | 2019 | Not randomized controlled trial | GL and WH | July 2023 |
| 113 | Shoulder stiffness and rotator cuff repair | Papalia | 2012 | Not randomized controlled trial | GL and WH | July 2023 |
| 114 | The Effect of Shoulder Mobilization on Scapular and Shoulder Muscle Activity During Resisted Shoulder Abduction: A Crossover Study of Asymptomatic Individuals | Patterson | 2020 | Not randomized controlled trial | GL and WH | July 2023 |
| 115 | Effectiveness of rehabilitation for patients with subacromial impingement syndrome | Sauers | 2005 | Not randomized controlled trial | GL and WH | July 2023 |
| 116 | The analgesic effect of joint mobilization and manipulation in tendinopathy: a narrative review | Savva | 2021 | Not randomized controlled trial | GL and WH | July 2023 |
| 117 | Soft Tissue Mobilization and PNF Improve Range of Motion and Minimize Pain Level in Shoulder Impingement | Al Dajah | 2014 | Included other inventions except mobilization | GL and WH | July 2023 |
| 118 | Mobilization with movement and kinesiotaping compared with a supervised exercise program for painful shoulder: results of a clinical trial | Djordjevic | 2012 | Included other inventions except mobilization | GL and WH | July 2023 |
| 119 | Immediate Effect of Mobilization vs Myofascial Release on Pain and Range of Motion in Patients with Shoulder Impingement Syndrome: A Pilot Randomized Trial | Nigam Prasad Dash | 2020 | Included other inventions except mobilization | GL and WH | July 2023 |
| 120 | Comparison of conservative treatment with and without manual physical therapy for patients with shoulder impingement syndrome: A prospective, randomized clinical trial | Şenbursa | 2007 | Included other inventions except mobilization | GL and WH | July 2023 |
| 121 | The effectiveness of manual therapy in supraspinatus tendinopathy | Şenbursa | 2011 | Included other inventions except mobilization | GL and WH | July 2023 |
