# Supplementary material for: Clinical efficacy of joint mobilization for shoulder impingement syndrome: a systematic review and meta-analysis
Source: PLoS One. 2026 Jul 13;21(7):e0352260. doi: 10.1371/journal.pone.0352260 (PMC13362101; doi:10.1371/journal.pone.0352260)
Supplement: S1 Text — S2 File. Search expressions. S3 File.The list of excluded studies with reasons. S4 File. The detailed data for the synthesis. (ZIP) [file pone.0352260.s001.zip › S4 The detailed data for the synthesis.docx]

**S1 Table. The detailed data for the synthesis.**

| First author | Methods | Outcome | Results | Data  extractor | Date  of Extraction |
| --- | --- | --- | --- | --- | --- |
| Akhtar (2020) | A: Mobilization(neuro) + Routine physical therapy (n=40)  B: Routine physical therapy (n=40) | (1)VAS (10)  (2) UCLA rating score (functional disability) | **1. Mean difference (Post–Pre)**  (1) After 5 weeks; A: -4.8, B: -1.8  After 11 weeks; A: -4.8, B: -1.9  (2) After 5 weeks; A: 13.9, B: 4.6  After 11 weeks; A: 14.5, B: 5.6  *all: (*p* < 0.001)  **2. Comparison between groups**  After 11 weeks  (1) A > B (*p* < 0.001)  (2) A > B (*p* < 0.001) | GL and WH | July 2023 |
| Aytar (2015) | A: scapular mobilization (n=22)  B: sham scapular mobilization (n=22)  C: supervised exercise (streching, strengthening) (n=22)  (all: TENS & hot pack)  (end of 3^rd^ week~: same exercises as done in the SE group for all groups) | (1) Quick DASH  (2) VAS (10)  (3) shoulder AROM  (4) Participant Satisfaction | **1. Mean difference (Post–Pre)**  (1) 2 weeks after; A: -9.3, B: -10.7, C: -7.9  3 weeks after; A: -11.7, B: -16.1, C: -10.3  7 weeks after; A: -21.9, B: -22.9, C: -14.1  11 weeks after; A: -12.7, B: -17.4, C: -17  (2) *pain at rest*; 2 weeks after; A: -0.3, B: -0.6, C: -1.0  3 weeks after; A: -1.6, B: -1.1, C: -1.9  7 weeks after; A: -3.1, B: -1.8, C: -2.7  11 weeks after; A: -3, B: -1.4, C: -2.5  *pain at night;* 2 weeks after; A: -2.7, B: -1.3, C: -2.5  3 weeks after; A: -3.0, B: -3.3, C: -3.6  7 weeks after; A: -3.7, B: -3.5, C: -3.6  11 weeks after; A: -4.2, B: -3.6, C: -4.0  *pain with activity;* 2 weeks after; A: -2.0, B: -1.7, C: -2.0  3 weeks after; A: -3.2, B: -2.5, C: -3.1  7 weeks after; A: -4.0, B: -3.5, C: -3.5  11 weeks after; A: -4.7, B: -3.6, C: -4.2  (3) *① flexion;*  2 weeks after; A: 9, B: 7, C: 11  3 weeks after; A: 13, B: 12, C: 16  7 weeks after; A: 13, B: 12, C: 19  11 weeks after; A: 13, B: 11, C: 20  *② external rotation;*  2 weeks after; A: 6, B: 3, C: 7  3 weeks after; A: 10, B: 10, C: 12  7 weeks after; A: 13, B: 8, C: 14  11 weeks after; A: 17, B: 9, C: 13  *③ internal rotation;*  2 weeks after; A: 2, B: 5, C: 1  3 weeks after; A: 6, B: 13, C: 9  7 weeks after; A: 7, B: 9, C: 11  11 weeks after; A: 3, B: 12, C: 10  **2. Comparison between groups**  (1) (*p* = 0.83)  3 weeks after; B > A > C  7 weeks after; B > A > C  11 weeks after; B > C > A  (2) pain at rest; (*p* = 0.28)  2 weeks after; C > A > B  3 weeks after; C > A > B  7 weeks after; A > C > B  11 weeks after; A > C > B  pain at night; (*p* = 0.47)  2 weeks after; A > C > B  3 weeks after; C > B > A  7 weeks after; A > C > B  11 weeks after; A > C > B  pain with activity; (*p* = 0.77)  2 weeks after; A = C > B  3 weeks after; A > C > B  7 weeks after; A > B = C  11 weeks after; A > C > B  2 weeks after; B > A > C  (3) ① flexion; (*p* = 0.41)  2 weeks after; C > A > B  3 weeks after; C > A > B  7 weeks after; C > A > B  11 weeks after; C > A > B  ② external rotation; (*p* = 0.55)  2 weeks after; C > A > B  3 weeks after; C > A = B  7 weeks after; C > A > B  11 weeks after; A > C > B  ③ internal rotation; (*p* = 0.19)  2 weeks after; B > A > C  3 weeks after; B > C > A  7 weeks after; C > B > A  11 weeks after; B > C > A | GL and WH | July 2023 |
| Beaudreuil (2011) | A: dynamic humeral centering + HE (n=35)  B: non-specific(sham) mobilization + HE (n=34) | (1) Constant score  (2) Medication (%) | **1. Mean difference (Post–Pre)**  (1) ① Pain (0-15); After 3 months; A: 4.5, B: 3.5  After 12 months; A: 5.4, B: 4.4  ② Activity (0-20); After 3 months; A: 5.3, B: 3.9  After 12 months; A: 6.5, B: 5.4  ③ Mobility (0-40); After 3 months; A: 8.8, B: 6.7  After 12 months; A: 12.4, B: 12.3  ④ Strength (0-25); After 3 months; A: 2.2, B: 1.4  After 12 months; A: 1.6, B: 1.3  ⑤ Total (0-100); After 3 months; A: 20.7, B: 15.4  After 12 months; A: 25.8, B: 23.4  (2) After 3 months; A: -16.5, B: -20.2  After 12 months; A: -17.5, B -18.7  **2. Comparison between groups**  (1) ① Pain; After 3 months; A > B (*p* = 0.004)  After 12 months; A > B (*p* = 0.012)  ② Activity; After 3 months; A > B (*p* = 0.08)  After 12 months; A > B (*p* = 0.11)  ③ Mobility; After 3 months; A > B (*p* = 0.17)  After 12 months; A > B (*p* = 0.92)  ④ Strength; After 3 months; A > B (*p* = 0.23)  After 12 months; A > B (*p* = 0.38)  ⑤ Total; After 3 months; A > B (*p* = 0.09)  After 12 months; A > B (*p* = 0.51)  (2) After 3 months; A > B (*p* = 0.012)  After 12 months; A > B (*p* = 0.05) | GL and WH | July 2023 |
| Conroy (1998) | A: Mobilization (n=7)  B: No mobilization (n=7)  (both: (hot packs, AROM, streching and exercises, soft tissue mobilizaion, paitient education) | (1) VAS (100)  (2) AROM | **1. Mean difference (Post–Pre)**  (1) ① 24-hour pain; A: -37.21, B: -2.21  ② Subacromial compression test; A: -28.07, B: -3.14  (2) abduction; A: 30.71, B: 23.00  elevation; A: 32.72, B: 14.86  external rotation; A: 18.23, B: 10.00  internal rotation; A: 15.15, B: 11.86  **2. Comparison between groups**  (1) ① 24-hour pain; A > B (*p* = 0.008)  ② Subacromial compression test; A > B (*p* = 0.032)  (2) abduction; A > B (*p* = n.s)  elevation; A > B (*p* = n.s)  external rotation; A > B (*p* = n.s)  internal rotation; A > B (*p* = n.s) | GL and WH | July 2023 |
| Cook (2014) | A: Mobilization (neck and shoulder) + GE (n=36)  B: Mobilization (shoulder only) + GE (n=32) | (1) Quick DASH  (2) NPRS (10)  (3) PASS | **1. Mean difference (Post–Pre)**  (1) A: -19.4, B: -24.7  (2) A: -3.4, B: -3.9  **2. Comparison between groups**  (1) A < B (*p* = 0.20)  (2) A < B (*p* = 0.42) | GL and WH | July 2023 |
| Delgado-Gil (2015) | A: mobilization (MWM) (n=21)  B: sham manual contact (n=21) | (1) NPRS (10)  (2) AROM (°) | 1. **Mean difference (Post–Pre)**  (1) Shoulder pain 24H; A: -0.6, B: 0.2  Shoulder pain at night; A: -1.7, B: -0.7  Pain with shoulder flexion; A: -1.1, B: 0.3  (2) Pain-free shoulder flexion; A: 31.0, B: -3.2  Maximum shoulder flexion; A: 20.1, B: 0.9  Shoulder extension; A: 1.2, B: 0.0  Shoulder abduction; A: 7.2, B: 5.8  Shoulder external rotation; A: 6.8, B: -1.4  Shoulder medial rotation; A: 6.3, B: 0.2  **2. Comparison between groups**  (1) Shoulder pain 24H; A > B (*p* = 0.171)  Shoulder pain at night; A > B (*p* = 0.191)  Pain with shoulder flexion; A > B (*p* = 0.011)  (2) Pain-free shoulder flexion; A > B (*p* < 0.001)  Maximum shoulder flexion; A > B (*p* < 0.01)  Shoulder extension; A > B (*p* = 0.532)  Shoulder abduction; A > B (*p* = 0.821)  Shoulder external rotation; A > B (*p* <0.01)  Shoulder medial rotation; A > B (*p* = 0.121) | GL and WH | July 2023 |
| Eliason (2021) | A: mobilization with guided exercise  + home exercise (n=29)  B: Guided exercise + home exercise (n=52)  C: No treatment (n=39) | (1) Constant-Murley score  (2) VAS(100) in AROM | 1. **Mean difference (Post–Pre)**  (1) ① Total; 6 weeks after; A: 13.5, B: 11.2, C: 6.2  12 weeks after; A: 24.2, B: 20.8, C: 11.2  6 months after; A: 27.8, B: 28.3, C: 17.6  ② Pain; 6 weeks after; A: 4.2, B: 4.2, C: 1.4  12 weeks after; A: 6.2, B: 6.7, C: 3.1  6 months after; A: 8.1, B: 8.1, C: 4.9  (2) ①Flexion  6 weeks after; A: -18(3), B: -3(-2), C: -4(-4)  12 weeks after; A: -26(-18), B: -21(-8), C: -15(-5)  6 months after; A: -28(-33), B: -30(-29), C: -30(16)  ② Abduction  6 weeks after; A: -17.5(-3), B: -19(-1), C: -18(-18)  12 weeks after; A: -31(-39), B: -31(-12), C: -30(-3)  6 months after; A: -31(-29), B: -42.5(-13), C: -40.5(-25)  ③ External Rotation  6 weeks after; A: -11(5), B: -16.5(-10), C: 2(18)  12 weeks after; A: -24(-58), B: -25.5(-18), C: -12(6)  6 months after; A: -25(-28), B: -34(-5), C: -18(-16)  ④ Internal Rotation  6 weeks after; A: -13.5(-9), B: -16(-1), C: -3(3)  12 weeks after; A: -19(-10), B: -35(-2), C: -11(-22)  6 months after; A: -25(0), B: -43(-4), C: -24(1)  **2. Comparison between groups**  (1) ① 6 weeks after; A > C(*p* = 0.0047), B > C(*p* = 0.022), A > B(*p* = n.s.)  12 weeks after; A > C(*p* = 0.00003), B > C(*p* = 0.0003), A > B(*p* = n.s.)  6 months after; A > C(*p* = 0.0005), B > C(*p* = 0.00377), A > B(*p* = n.s.)  ② 6 weeks after; A > C(*p* = 0.002), B > C(*p* = 0.01), A > B(*p* = n.s.)  12 weeks after; A > C(*p* = 0.0009), B > C(*p* = 0.001), A > B(*p* = n.s.)  6 months after; A > C(*p* = 0.002), B > C(*p* = 0.005), A > B(*p* = n.s.)  (2) VAS in pain-free AROM  ①Flexion  6 weeks after; A > B(*p* = 0.008), A > C(*p* = 0.005), C > B(*p* = n.s.)  12 weeks after; A > B(*p* = n.s.), A > C(*p* = 0.000), C > B(*p* = 0.017)  6 months after; A < B(*p* = n.s.), A < C(*p* = n.s.), B = C(*p* = n.s.)  ② Abduction  6 weeks after; A < B(*p* = 0.020), A < C(*p* = 0.039), B > C(*p* = n.s.)  12 weeks after; A = B(*p* = 0.000), A > C(*p* = 0.000), B > C(*p* = n.s.)  6 months after; A < B(*p* = n.s.), A < C(*p* = n.s.), B > C(*p* = n.s.)  ③ External Rotation  6 weeks after; A < B(*p* = 0.023), A > C(*p* = 0.004), B > C(*p* = n.s.)  12 weeks after; A < B(*p* = 0.005), A > C(*p* = 0.003), B > C(*p* = n.s.)  6 months after; A < B(*p* = n.s.), A > C(*p* = n.s.), B > C(*p* = n.s.)  ④ Internal Rotation  6 weeks after; A < B(*p* = 0.017), A > C(*p* = 0.001), B > C(*p* = n.s.)  12 weeks after; A < B(*p* = n.s.), A > C(*p* = 0.004), B > C(*p* = n.s.)  6 months after; A < B(*p* = n.s.), A > C(*p* = 0.015), B > C(*p* = n.s.) | GL and WH | July 2023 |
| Guimarães (2016) | A: 4 sessions of mobilization(MWM)  -> 4 week sham technique (n=14)  B: 4 sessions of sham technique  -> 4 week mobilization(MWM) (n=13) | (1) AROM(°)  (2) Isometric Peak Force (Kp)  (3) DASH  (4) SPADI (%) | 1. **Mean difference (Post–Pre)**  (1) ① External Rotation  Interchange; A: 10.5, B: 6.1  Postintervention: A: 2.4, B: 14.31  ② Abduction  Interchange; A: 8.9, B: 15.4  Postintervention: A: 19.1, B: 23.1  ③ Scaption  Interchange; A: 2.6, B: 10.0  Postintervention: A: -2.1, B: 0.2  ④ Flexion  Interchange; A: 3.3, B: -1.6  Postintervention: A: 7.6, B: 3.7  (2) ① External Rotation  Interchange; A: 0.9, B: 0.0  Postintervention: A: 1.1, B: 0.0  ② Abduction  Interchange; A: 0.3, B: -0.2  Postintervention: A: 0.9, B: 0.3  ③ Scaption  Interchange; A: 0.3, B: 0.3  Postintervention: A: 1.1, B: 0.1  ④ Flexion  Interchange; A: 0.6, B: -0.3  Postintervention: A: 0.7, B: 0.4  (3) DASH  Interchange; A: -3.6, B: -3.1  Postintervention: A: -7.9, B: -6.9  (4) SPADI  ① Disability  Interchange; A: -2.8, B: -4.6  Postintervention: A: -7.0, B: -8.6  ② Pain  Interchange; A: -10.4, B: -11.5  Postintervention: A: -11.9, B: -17.1  ③ Total  Interchange; A: -4.3, B: -7.3  Postintervention: A: -7.5, B: -12.1  **2. Comparison between groups**  (1) ① External Rotation  Interchange; A > B (*p* > 0.05)  Postintervention: A: < B (*p* > 0.05)  ② Abduction  Interchange; A < B (*p* > 0.05)  Postintervention: A < B (*p* < 0.05)  ③ Scaption  Interchange; A > B (*p* > 0.05)  Postintervention: A > B (*p* > 0.05)  ④ Flexion  Interchange; A > B (*p* > 0.05)  Postintervention: A > B (*p* > 0.05)  (2) ① External Rotation  Interchange; A > B (*p* > 0.05)  Postintervention: A > B (*p* > 0.05)  ② Abduction  Interchange; A > B (*p* > 0.05)  Postintervention: A > B (*p* > 0.05)  ③ Scaption  Interchange; A = B (*p* > 0.05)  Postintervention: A > B (*p* > 0.05)  ④ Flexion  Interchange; A > B (*p* > 0.05)  Postintervention: A > B (*p* > 0.05)  (3) DASH  Interchange; A < B (*p* < 0.05)  Postintervention: A < B (*p* < 0.05)  (4) SPADI  ① Disability  Interchange; A > B (*p* > 0.05)  Postintervention: A > B (*p* < 0.05)  ② Pain  Interchange; A > B (*p* < 0.05)  Postintervention: A > B (*p* < 0.05)  ③ Total  Interchange; A > B (*p* < 0.05)  Postintervention: A > B (*p* < 0.05) | GL and WH | July 2023 |
| Gutiérrez-Espinoza (2023) | A: scapular mobilization + exercise program (n=36)  B: exercise program (n=36) | (1) DASH  (2) Constant-Murley score  (3) VAS (10)  (4) scapular UR | 1. **Mean difference (Post–Pre)**  (1) A: -27.9, B: -26.8  (2) A: 26.2, B: 24.1  (3) at rest; A: -1.3, B: -1.2  during movement; A: -3.2, B: -3  (4) at rest; A: 2.7, B: 3.3  at 45˚; A: 2.1, B: 2.9  at 90˚; A: 0.7, B: 0.8  at 135˚; A: 0.5, B: 0.4  **2. Comparison between groups**  (1) A > B (*p* = 0.911)  (2) A < B (*p* = 0.841)  (3) at rest; A > B (*p* = 0.684)  during movement; A > B (*p* = 0.764)  (4) at rest; A < B (*p* = 0.237)  at 45˚; A < B (*p* = 0.096)  at 90˚; A < B (*p* = 0.783)  at 135˚; A < B (*p* = 0.886) | GL and WH | July 2023 |
| İğrek (2022) | A: Scapular mobilization + conventional physiotherapy(n=15)  B: Proprioceptive neuromuscular Facilitation + conventional physiotherapy(n=15)  C: conventional physiotherapy(n=14)  *conventional: electrotherapy and therapeutic exercise | (1) VAS (10)  (2) DASH  (3) Constant-Murley score  (4) AROM  (5) Muscle strength (kg) | 1. **Mean difference**  (1) ① at rest; 2 weeks after; A: -2.0, B: -2.0, C: -2.0  4 weeks after; A: -3.3, B: -3.2, C: -3.0  16 weeks after; A: -3.3, B: -3.2, C: -3.0  ② in activity; 2 weeks after; A: -4.6, B: -4.1, C: -3.7  4 weeks after; A: -6.1, B: -5.9, C: -5.2  16 weeks after; A: -5.7, B: -4.6, C: -3.9  ③ at night; 2 weeks after; A: -2.6, B: -2.6, C: -2.5  4 weeks after; A: -4.2, B: -4.2, C: -3.8  16 weeks after; A: -5.3, B: -4.9, C: -4.0  (2) ① Disability/symptom; 2 weeks after; A: -24.9, B: -24.4, C: -16.0  4 weeks after; A: -39.5, B: -37.6, C: -32.9  16 weeks after; A: -50.1, B: -46.4, C: -37.6  ② Work; 2 weeks after; A: -26.1, B: -22.3, C: -14.4  4 weeks after; A: -43.4, B: -35.0, C: -33.5  16 weeks after; A: -52.0, B: -44.8, C: -39.5  (3) 2 weeks after; A: 20.0, B: 17.9, C: 14.0  4 weeks after; A: 34.3, B: 28.0, C: 24.8  16 weeks after; A: 39.9, B: 33.8, C: 29.1  (4) ① flexion; 2 weeks after; A: 25.6, B: 27.4, C: 12.5  4 weeks after; A: 39.7, B: 35.6, C: 21.2  16 weeks after; A: 45.0, B: 40.4, C: 21.7  ② Extension; 2 weeks after; A: 5.7, B: 6.0, C: 6.0  4 weeks after; A: 10.0, B: 8.9, C: 10.0  16 weeks after; A: 10.7, B: 8.9, C: 9.8  ③ Abduction; 2 weeks after; A: 27.9, B: 27.7, C: 15.1  4 weeks after; A: 43.1, B: 43.2, C: 24.9  16 weeks after; A: 48.9, B: 48.9, C: 26.9  ④ Adduction; 2 weeks after; A: 5.1, B: 5.8, C: 4.4  4 weeks after; A: 8.2, B: 8.5, C: 8.5  16 weeks after; A: 7.6, B: 9.5, C: 8.7  ⑤ Internal Rotation; 2 weeks after; A: 13.2, B: 9.4, C: 7.6  4 weeks after; A: 19.7, B: 15.0, C: 12.4  16 weeks after; A: 22.1, B: 17.9, C: 11.7  ⑥ External Rotation; 2 weeks after; A: 1.0, B: 12.3, C: 6.9  4 weeks after; A: 10.2, B: 20.7, C: 12.4  16 weeks after; A: 2.3, B: 23.9, C: 11.6  (5) -  **2. Comparison between groups**  (1) ① 2 weeks after; A = B = C (*p* = 0.935)  4 weeks after; A > B > C (*p* = 0.465)  16 weeks after; A > B > C (*p* = 0.515)  ② 2 weeks after; A > B > C (*p* = 0.053), A > C (*p* = 0.019)  4 weeks after; A > B > C (*p* = 0.291)  16 weeks after; A > B > C (*p* = 0.032), A > C (*p* = 0.011)  ③ 2 weeks after; A = B > C (*p* = 0.737)  4 weeks after; A = B > C (*p* = 0.536)  16 weeks after; A > B > C (*p* = 0.071), A > C (*p* = 0.027)  (2) ① 2 weeks after; A > B > C (*p* = 0.024), A > C (*p* = 0.018). B > C (*p* = 0.017)  4 weeks after; A > B > C (*p* = 0.523)  16 weeks after; A > B > C (*p* = 0.044), A > C (*p* = 0.015)  ② 2 weeks after; A > B > C (*p* = 0.033), A > C (*p* = 0.004)  4 weeks after; A > B > C (*p* = 0.124)  16 weeks after; A > B > C (*p* = 0.030), A > C (*p* = 0.011)  (3) 2 weeks after; A > B > C (*p* = 0.013), A > C (*p* = 0.013), B > C (*p* = 0.011)  4 weeks after; A > B > C (*p* = 0.092)  16 weeks after; A > B > C (*p* = 0.020), A > C (*p* = 0.007)  (4) ① 2 weeks after; B > A > C (*p* = 0.002), A > C (*p* = 0.002), B > C (*p* = 0.003)  4 weeks after; A > B > C (*p* = 0.008), A > C (*p* = 0.009), A > B (*p* = 0.009)  16 weeks after; A > B > C (*p* = 0.011), A > C (*p* = 0.005), B > C (*p* = 0.018)  ② 2 weeks after; B = C > A (*p* = 0.702)  4 weeks after; A = C > B (*p* = 0.442)  16 weeks after; A > C > B (*p* = 0.658)  ③ 2 weeks after; A > B > C (*p* = 0.005), A > C (*p* = 0.001), B > C (*p* = 0.023)  4 weeks after; B > A > C (*p* = 0.035), B > C (*p* = 0.012)  16 weeks after; A = B > C (*p* = 0.001), A > C (*p* < 0.001), B > C (*p* = 0.008)  ④ 2 weeks after; B > A > C (*p* = 0.726)  4 weeks after; B = C > A (*p* = 0.395)  16 weeks after; B > C > A (*p* = 0.608)  ⑤ 2 weeks after; A > B > C (*p* = 0.007), A > C (*p* = 0.002), B > C (*p* = 0.036)  4 weeks after; A > B > C (*p* = 0,472)  16 weeks after; A > B > C (*p* = 0.002), A > C (*p* < 0.001)  ⑥ 2 weeks after; B > C > A (*p* = 0.002), C > A (*p* = 0.018), B > C (*p* < 0.001)  4 weeks after; B > C > A (*p* = 0.257)  16 weeks after; B > C > A (*p* = 0.004), C > A (*p* = 0.013), B > C (*p* < 0.002)  (5) - | GL and WH | July 2023 |
| Kachingwe  (2008) | A: mobilization of GH joint + Supervised Exercise (n=9)  B: MWM + Supervised Exercise (n=9)  C: Supervised Exercise (n=8)  D: Unsupervised Exercise, advice (n=7) | (1) VAS  (2) AROM  (3) SPADI (point) | **1. Mean difference (Post–Pre)**  Percentage of change from pre- to post-treatment for each variables  (1) A: 44.2, B: 55.2, C: 20.8, D: 14.4  (2) Flexion; A: -15.9, B: 46.7, C: 27.6, D: 42.6  Scaption; A: 2.5, B: 66.5, C: 19.8, D: 29.8  (3) A: 56.7, B: 55.5, C: 61.6, D: 34.2  **2. Comparison between groups**  (1) B > A > C > D (*p* > 0.05)  (2) Flexion; B > D > C > A (*p* > 0.05)  Scaption; B > D > C > A (*p* > 0.05)  (3) C > A > B > D (*p* > 0.05) | GL and WH | July 2023 |
| Kulakli (2020) | A: mobilization after SACS injection (n=42)  B: SACS injection (n=42) | (1) AROM (°)  (2) VAS (10)  (3) DASH | 1. **Mean difference (Post–Pre)**  (1) ① shoulder flexion;  1 week after; A: 16.00, B: 7.50  4 weeks after; A: 40.00, B: 37.00  ② shoulder Abduction  1 week after; A: 28.75, B: 23.25  4 weeks after; A: 53.75, B: 49.00  ③ shoulder Internal Rotation  1 week after; A: 12.25, B: 9.00  4 weeks after; A: 17.00, B: 16.75  ④ shoulder External Rotation;  1 week after; A: 11.25, B: 11.75  4 weeks after; A: 15.75, B: 16.00  (2) ① rest  1 week after; A: -3.25, B: -3.1  4 weeks after; A; -3.8, B: -4.2  ② activity  1 week after; A: -5.75, B: -4.35  4 weeks after; A: -6.70, B: -4.20  (3) 1 week after; A: -3.20, B: -3.35  4 weeks after; A: -21.85, B: -20.90  **2. Comparison between groups**  (1) ① shoulder flexion;  1 week after; A > B (*p* = 0.008)  4 weeks after; A > B (*p* = 0.791)  ② shoulder Abduction  1 week after; A > B (*p* = 0.036)  4 weeks after; A > B (*p* = 0.214)  ③ shoulder Internal Rotation  1 week after; A > B (*p* = 0.363)  4 weeks after; A > B (*p* = 0.424)  ④ shoulder External Rotation;  1 week after; A < B (*p* = 0.455)  4 weeks after; A < B (*p* = 0.396)  (2) ① rest  1 week after; A > B (*p* = 0.331)  4 weeks after; A > B (*p* = 0.824)  ② activity  1 week after; A > B (*p* = 0.028)  4 weeks after; A > B (*p* = 0.086)  (3) 1 week after; A < B (*p* = 0.605)  4 weeks after; A > B (*p* = 0.556) | GL and WH | July 2023 |
| Menek (2019) | A: mulligan mobilization(MWM)  + Exercise + Cold Pack + USTENS + finger staircase (n=15)  B: Exercise + Cold Pack + USTENS + finger staircase (n=15) | (1) VAS (100)  (2) shoulder AROM  (3) DASH  (4) SF-36 | **1. Mean difference (Post–Pre)**  (1) rest; A: -5.20, B: -3.06  activity; A: -6.14, B: -3  (2) flexion; A: 70.00, B: 32.33  extension; A: 23.00, B: 8.66  abduction; A: 74.66, B: 31.33  external rotation; A: 58.00, B: 21.66  internal rotation; A: 40.33, B: 15.33  (3) A: -33.05, B: -19.36  (4) PF; A: 15.34, B: 21.66  RP; A: 43.34, B: 26.67  BP; A: 20.53, B: 25.73  GH; A: 6.20, B: 2.67  VT; A: 9.53, B: 8.60  SF; A: 26.80, B: 5.87  RE; A: 15.27, B: 4.60  MH; A: 3.47, B: 4.73  **2. Comparison between groups**  (1) rest; A > B (*p* = 0.00)  activity; A > B (*p* = 0.00)  (2) flexion; A > B (*p* = 0.00)  extension; A > B (*p* = 0.00)  abduction; A > B (*p* = 0.00)  external rotation; A > B (*p* = 0.00)  internal rotation; A > B (*p* = 0.  (3) A > B (*p* = 0.01)  (4) PF; A > B (*p* = 0.28)  RP; A > B (*p* = 0.14)  BP; A < B (*p* = 0.43)  GH; A > B (*p* = 0.50)  VT; A > B (*p* = 0.86)  SF; A > B (*p* = 0.00)  RE; A > B (*p* = 0.47)  MH; A < B (*p* = 0.78) | GL and WH | July 2023 |
| Neelapala  (2016) | A: MWM + HE  B: HE | (1) VAS (10)  (2) Scapular UR  (3) strength (lbs) | **1. Mean difference (Post–Pre)**  (1) performing overhead shoulder movements; A: -3.38, B: -1.00  (2) Scapular upward rotation; A: 0.9, B: -0.4  (3) External Rotation; A: 9.1, B: 0.25  Internal Rotation; A: 2.37, B: 0.19  **2. Comparison between groups**  (1) A > B (*p* < 0.01)  (2) Scapular upward rotation; A > B (*p* = 0.7)  (3) External Rotation; A > B (*p* = 0.04)  Internal Rotation; A > B (*p* = 0.8) | GL and WH | July 2023 |
| Nigam (2020) | A: Mobilization + thoracic thrust manipulation (n=10)  B: Myofascial relase on Subscapularis, supraspinatus, infraspinatus, pectoralis major (n=10) | (1) VAS (10)  (2) Shoulder AROM | 1. **Mean difference (Post–Pre)**  (1) 1^st^ session after; A: -1.7, B: -1.7  1^st^ week after; A: -2.9, B: -3.8  (2) ① abduction  1^st^ session after; A: 3.8, B: 6  1^st^ week after; A: 20.8, B: 19  ② Flexion  1^st^ session after; A: 6, B: 12.8  1^st^ week after; A: 28, B: 32.5  **2. Comparison between groups ( p-value: not mentioned )**  (1) 1^st^ session after; A = B  1^st^ week after; A < B  (2) ① abduction  1^st^ session after; A < B  1^st^ week after; A > B  ② Flexion  1^st^ session after; A < B  1^st^ week after; A < B | GL and WH | July 2023 |
| Park (2020) | A: mobilization (15 min)) (n=10)  B: exercise (15 min) (n=10)  C: combination (M 7min 30s, E 7min 30s) (n=10) | (1) Thoracic kyphosis angle  (2) Muscle tone (Hz)  (3) muscle stiffness (N/m)  (4) PROM  (5) SPADI (point) | **1. Mean difference (Post–Pre)**  (1) A: -3.5, B: -2.7, C: -5.1  (2) UT; A: -1.16, B: -1.06, C: -1.83  PM; A: -0.78, B: -0.76, C: -0.88  (3) UT; A: -25.40, B: -21.60, C: -34.50  PM; A: -17.2, B: -15.40, C: -17.20  (4) flexion; A: 7.8, B: 7.9, C:12.7  abduction; A: 7.8, B: 7.4, C: 10.6  medial rotation; A: 2.5, B: 2.9, C: 5.2  lateral rotation; A: 2.2, B: 2.5, C: 5.5  (5) pain; A: -13.00, B: -13.8, C: -18.6  disability; A: -11.75, B: -10.37, C: -16.75  total; A: -12.23, B: -11.69, C: -17.46  **2. Comparison between groups**  (1) C > A > B (*p* = 0.011)  (2) UT; C > A > B (*p* = 0.046)  PM; C > A > B (*p* = 0.118)  (3) UT; C > A > B (*p* = 0.702)  PM; C = A > B (*p* = 0.749)  (4) flexion; C > B > A (*p* = 0.025)  abduction; C > A > B (*p* = 0.131)  medial rotation; C > B > A (*p* = 0.026)  lateral rotation; C > B > A (*p* = 0.018)  (5) pain; C > B > A (*p* = 0.001)  disability; C > A > B (*p* = 0.007)  total; C > A > B (*p* = 0.002) | GL and WH | July 2023 |
| Pekgöz (2019) | A: mobilization + NMES  B: exercise + NMES | (1) PROM  (2) VAS (10)  (3) DASH  (4) ASES  (5) SF-36 | **1. Mean difference (Post–Pre)**  (1) flexion; A: 12.8, B: 14.3  extension; A: 4.6, B: 6.3  abduction; A: 26.1, B: 16.7  Internal rotation; A: 13.8, B: 15.2  External rotation; A: 5.2, B: 5.9  (2) motion; A: -3.4, B: -3.4  night; A: -3.1, B: -4.2  rest; A: -2.2, B: -3.1  (3) DASH; A: -16.2, B: -10.3  (4) ASES; A: -8.4, B: -11.1  (5) PF; A: 7.5, B: 12.8  RP; A: 5.0, B: 6.3  BP; A: 4.7, B: 2.4  GH; A: -2.0, B: 1.4  VT; A: -0.3, B: -1.0  SF; A: -11.3, B: -20.0  RE; A: 0.0, B: 3.2  MH; A: -4.8, B: 0.2  **2. Comparison between groups**  (1) flexion; A < B (*p* = 0.09)  extension; A < B (*p* = 0.30)  abduction; A > B (*p* = 0.84)  Internal rotation; A < B (*p* = 0.26)  External rotation; A < B (*p* = 0.38)  (2) motion; A = B (*p* = 0.92)  night; A < B (*p* = 0.18)  rest; A < B (*p* = 0.20)  (3) DASH; A > B (*p* = 0.18)  (4) ASES; A < B (*p* = 0.26)  (5) PF; A < B (*p* = 0.73)  RP; A < B (*p* = 0.68)  BP; A > B (*p* = 0.36)  GH; A < B (*p* = 0.08)  VT; A > B (*p* = 0.20)  SF; A > B (*p* = 0.17)  RE; A < B (*p* = 0.30)  MH; A < B (*p* = 0.90) | GL and WH | July 2023 |
| Satpute (2014) | A: mobilization(MWM) + Hot pack + HE (n=22)  B: GE + Hot pack + HE (n=22) | (1) pain free HBB (°)  (2) VAS (10) in HBB  (3) PROM  (4) SPADI (%) | **1. Mean difference (Post–Pre)**  (1) A: 16.32, B: 6.83  (2) With maximal HBB; A: -5.31, B: -3.39  (3) Internal rotation: A: 16,86, B: 7.38  (4) A: -40.63, B: -19.19  **2. Comparison between groups**  (1) A > B (*p* < 0.125)  (2) With maximal HBB; A > B (*p* < 0.125)  (3) Internal rotation: A > B (*p* < 0.125)  (4) A > B (*p* < 0.125) | GL and WH | July 2023 |
| Srivastava (2018) | A: mobilization(MWM) + Exercise (n=11)  B: cryotherapy + Exercise (n=11) | (1) VAS (10)  (2) AROM  (3) SPADI (%) | **1. Mean difference (Post–Pre)**  (1) 1^st^ session after; A: -0.9, B: -0.456  6^th^ session after; A: -2.36 B: -3.096  (2) abduction in the plane of scapular  1^st^ session after; A: 6.79, B: 3.13  6^th^ session after; A: 23.63, B: 21.82  (3) A: -17.77, B: -27.77  **2. Comparison between groups**  6^th^ session after; (1) A < B (*p* > 0.0z5)  (2) A > B (*p* > 0.05)  (3) A < B (*p* > 0.05) | GL and WH | July 2023 |
| Surenkok (2009) | A: scapular mobilization (n=13)  B; the sham (n=13)  C: the control (nothing) (n=13) | (1) AROM  (2) Scapular UR  (3) VAS (100)  (4) Constant shoulder score | **1. Mean difference (Post–Pre)**  (1) flexion; A: 7.84, B: 0.23, C: 1.69  abduction; A: 5.61, B: 0.54, C: 0.31  (2) A: 4.38, B: 0.23, C: 0.54  (3) activitiy; A: -0.46, B: -0.16, C: -0.92  rest; A: -1.46, B: -1.08, C: -0.43  (4) A: 2.23, B: 0.31, C: -3.77  **2. Comparison between groups**  (1) flexion; A > B (*p* < 0.016), A > C (*p* < 0.016)  abduction; A > B (*p* < 0.016), A > C (*p* < 0.016)  (2) A > B (*p* < 0.016), A > C (*p* < 0.016)  (3) activitiy; C > A > B (*p* = n.s)  rest; A > B > C (*p* = n.s)  (4) A > B > C (*p* < 0,016) | GL and WH | July 2023 |
