# Supplementary material for: Clinical efficacy of joint mobilization for shoulder impingement syndrome: a systematic review and meta-analysis
Source: PLoS One. 2026 Jul 13;21(7):e0352260. doi: 10.1371/journal.pone.0352260 (PMC13362101; doi:10.1371/journal.pone.0352260)
Supplement: Attachment — Submitted filename: Response to Reviewers.docx [file pone.0352260.s003.docx]

'Response to Reviewers: (PONE-D-24-07775)

Point 1: Please provide more detailed information regarding the inclusion and exclusion criteria used for selecting studies in your systematic review. This will enhance the transparency of your methodology.

Response 1: We modified [Inclusion and Exclusion criteria] using PICOs to organize our criteria according to PRISMA guidelines, added inclusion and exclusion, and clarified the primary and secondary outcomes.

[Inclusion and Exclusion criteria]

RCTs on mobilization alone or in combination with other interventions for SIS were selected from the retrieved literature. Studies with the terms “mobilization” and “impingement” in the title and abstract were included in the search results.

~~“As the aim of this review was to determine the effect of mobilization on SIS, we only included studies in which mobilization was the sole intervention in the mobilization group after excluding all other overlapping interventions from both the mobilization and comparison groups. Studies that did not meet these criteria were excluded.”~~

→Articles included in this systematic review were selected based on the participants, interventions, comparisons, outcomes, and study designs framework.

(1) Participants: Patients with SIS. (2) Interventions: The experimental group received the mobilization therapy alone or other interventions in addition to the mobilization. (3) Control: The control group received treatments other than the mobilization. If the experimental group has additional interventions beyond the mobilization, the control group has no interventions other than the same intervention. (4) Outcome: Diagnostic metrics in SIS, including VAS, ROM, DASH, SPADI, constant score, and more. (5) Study design: Only randomized controlled trials were included.

Exclusion criteria were as follows: (1)Non-randomized controlled trials, retrospective analyses, or case reports. (2)Articles with no available data or duplicate date.

In the retrieved articles, no restrictions were placed on subject age, sex, composition of the control group, treatment duration, follow-up period, or indices used in the study.

Point 2: Please ensure that the rationale for your chosen statistical tests is clearly stated. While you have identified several limitations, expanding this section to include potential biases in the included studies would strengthen your manuscript. Please review the formatting of your references to ensure consistency with the journal’s guidelines. A few references need to be double-checked for completeness.

Response 2: To ensure the rationale for the statistical tests we chose, we added supplementary explanations additional in [Data extraction] section

[Data extraction]

The meta-analysis was conducted using Cochrane’s Review Manager (RevMan) version 5.4 (Copenhagen, Denmark). Continuous outcomes were analyzed using the mean difference (MD) and 95% confidence interval (CI). For effect size analysis, we used SMD rather than OR or RR. Heterogeneity was assessed with Higgins’ I2 values, where values below 50% were considered indicative of low heterogeneity, and those above 50% indicated high heterogeneity. In addition to the I2 assessment for heterogeneity, we used a Random-Effects Model, which allows for differences in true effect sizes between studies and is used when there is heterogeneity, and is weighted using the inverse variance method.

Point 3: A numbered table of all studies identified in the literature search, including those that were excluded from the analyses. For every excluded study, the table should list the reason(s) for exclusion.

Response 3: We have added a table as S2 Table, which describes information on all studies excluded from the analysis and the reason for exclusion, data extractor and the date of data extraction.

Point 4: A table of all data extracted from the primary research sources for the systematic review and/or meta-analysis. The table (must include the following information for each study:

Name of data extractors and date of data extraction.

Confirmation that the study was eligible to be included in the review.

Response 4: We have added information about extractors and extraction dates to the S1 table.

In [Literature selection and analysis] section, we mentioned that we confirmed the eligibility of the studies included in this review, in the sentence below:

Initially, the titles and abstracts of the retrieved articles were screened. Subsequently, the study design, target patients, interventions, control groups, indices, and outcomes of the selected articles were analyzed, summarized, and reviewed in detail. Studies that met the inclusion and exclusion criteria were then selected.

Point 5: Please discuss the implications of the high level of heterogeneity between studies, and the high risk of bias.

Response 5: To explain this point, we modified [Limitation] section as below

This systematic review was conducted to determine whether the use of mobilization to improve SIS symptoms can achieve significant clinical benefits. However, this study had several limitations. First, there was high heterogeneity of intervention methods in the studies included in this review. The literature search found only three studies [24, 32, 36] that included mobilization alone as an intervention, which is a small number. Therefore, we widened the scope of the mobilization group and did not limit the types of mobilization. Several differences existed among the studies, such as variations in the frequency of each treatment per week, the total duration of the study in weeks, and the type of physical therapy used, making it difficult to determine the most clinically beneficial technique. Second, the duration of follow-up in each study was inconsistent. While a few studies [9, 21, 27] with longer follow-up periods were available, the number was too small to allow for statistical analysis between groups. Consequently, we were unable to determine the long-term clinical effects. As this may affect the interpretation of the results, we conducted sub-analyses using the same criteria to minimize heterogeneity. We also considered that different outcome index between studies may make it difficult to synthesise results, so we used methods to standardise different outcome index or to use integrated analysis of the results to address this. Third, nine of the 19 studies had 20 or fewer patients in each group. This absolute lack of sufficient sample size ~~may diminish the reliability of the findings~~ result in insufficient statistical power. Small studies can increase random error and affect the reliability of meta-analysis results, thereby complicating the ability to express confidence in their effectiveness in real-world clinical practice. To compensate for this, we set appropriate confidence intervals for our analyses in the meta-analyses. Fourth, the assessment indices used in each study were too diverse to allow for a detailed subgroup analysis. In the case of the AROM, various types of shoulder joint movements were presented in each study. Therefore, although a large number of studies presented the AROM as an index, the number of studies that could be used for each movement-specific analysis was small. Finally, the majority of studies were assessed as having “some concern” and “high risk” of bias due to failures in randomization and reliable blinding and the omission and correction of accurate outcomes, resulting in a high risk of bias.

Point 6: Please explain how missing data were handled.

Response 6: To explain how missing data were handled, we added below in [Data extraction].

One of the studies included in this study [9] had missing data for certain variables. This represents approximately 0.01% of the total data and was considered to be missing at random. Therefore, we used a method that did not include missing data in the analysis, and we also performed a sensitivity analysis to assess the robustness of the results.

Point 7: Please review your reference list to ensure that it is complete and correct.

Response 7: We found that some of the references were not formatted according to the journal's guidelines, so we have modified the reference list as follows

7.	Şenbursa G, Atay ÖA. The effectiveness of manual therapy in Supraspinatus Tendinopathy. Acta Orthop Traumatol Turc 2011;45(3):162-67.

20.	Kim SY, Seo HJ, Shin SS, Lee SJ, Jang BH, Cha YJ, Choi MY, Park JY, Suh YS. NECA's guidance for assessing tools of risk of bias. Seoul: National Evidencebased Healthcare Collaborating Agency; 2021.

31.	Yvr Neelapala YRSRRD. Effect of Mulligan's posterolateral glide on shoulder rotator strength, scapular upward rotation in shoulder pain subjects - A randomized controlled trial., J Musculoskelet Res, 2016, 19(3) | added to CENTRAL: 30 April 2017 | 2017 Issue 4 2016;19(2):-.

Point 8: Please ensure that your manuscript meets PLOS ONE's style requirements, including those for file naming.

Response 8 : We found that no headings were used for each title, so we used headings for each section that met the style required by PLOS ONE.

* We have additional corrections.

[1] We have corrected the notation of some authors' names, as detailed below.

(1) Ga-yeong Lee -> Gayeong Lee

(2) Won-Bae Ha -> Wonbae Ha

In addition, the initials of the authors listed in the text have also been changed.

(1) G.-Y. Lee -> GL

(2) W.-B. Ha -> WH

(3) J.-H. Lee -> JL

[2] One of the authors has changed their name to reflect this.

* Byeong-Hyeon Jeon -> Seojae Jeon

[3] In section [Literature selection], several typographical errors have been corrected.

[Literature selection]

In total, 197 articles were retrieved from the three online databases through April 2023, with 57 duplicates subsequently excluded. Titles and abstracts were then screened to exclude 92 irrelevant articles and 9 with unavailable full text, resulting in 101 exclusions. We also excluded 15 articles that were not RCTs. Furthermore, we excluded five studies with interventions other than mobilization in the mobilization group after excluding other overlapping interventions in both the mobilization and comparison groups, resulting in a final selection of 19 articles (Fig 1).

[4] In Fig. 1, several typographical errors have been corrected.

* Duplicate records removed (n = 56) -> Duplicate records removed (n = 57)

* Records screened (n = 141) -> Records screened (n = 140)

* Unrelated (n = 93) -> Unrelated (n = 92)

* Not Randomised control trial -> Not randomised controlled trial

Again, thank you for giving us the opportunity to strengthen our manuscript with your valuable comments. We have worked hard to incorporate your feedback and hope that these revisions persuade you to accept our submission.

Sincerely,

Gayeong Lee, and Wonbae Ha
