# Supplementary material for: Clinical efficacy of joint mobilization for shoulder impingement syndrome: a systematic review and meta-analysis
Source: PLoS One. 2026 Jul 13;21(7):e0352260. doi: 10.1371/journal.pone.0352260 (PMC13362101; doi:10.1371/journal.pone.0352260)
Supplement: Attachment — Submitted filename: Response_to_Reviewers_auresp_2.docx [file pone.0352260.s004.docx]

Response to Reviewers: (PONE-D-24-07775R1)

Point 1: Statistical Heterogeneity: Many meta-analyses reported high I² values, which compromise interpretability. You are advised to explore sources of heterogeneity through subgroup or sensitivity analyses and discuss their impact in the manuscript.

Response 1: We completely agree with the editor's point regarding high statistical heterogeneity. We already reported a high I² value in the body of the manuscript and performed subgroup analyses to address this, based on pain assessment time points (at rest, during activity, at night, all day, etc.) and specific movements within the joint range of motion (flexion, extension, etc.). In the revised manuscript, we will further strengthen the discussion on this point.

Additionally, in the 'Discussion' section, we will deeply address the potential clinical diversity as a cause of this statistical heterogeneity. This includes differences in manual therapy techniques between studies (e.g., neural mobilization, soft tissue mobilization, MWM), various co-interventions (type and intensity of exercise therapy), differences in the control group, and variations in treatment dosage (frequency, duration). Finally, we will once again emphasize the need for caution when interpreting the pooled results due to this high heterogeneity.

[Discussion] - [Summary of findings]

... ~~No adverse events were reported. The high risk of bias assessment and the high level of heterogeneity in the meta-analysis require caution in interpretation; however, in most indices, mobilization indicates a significant benefit.~~

→ No adverse events were reported in any of the included studies. The results of this meta-analysis should be interpreted with caution due to significant statistical heterogeneity, which primarily stems from the considerable clinical diversity among the included studies. This diversity was evident in several areas: the mobilization techniques used varied significantly, ranging from neuromobilization to MWM; co-interventions like exercise therapy were inconsistent in type and intensity; control groups were not uniform, including physical therapy alone, exercise, or sham therapy; and treatment dosages varied widely in frequency and duration. However, despite these limitations requiring cautious interpretation, mobilization demonstrated a significant benefit across most indices [14].

Point 2: Definition and Categorization of Interventions: The term “mobilization” is used to refer to a broad range of techniques. Clear operational definitions and subgroup analyses based on technique type are recommended.

Response 2: This is a very important point. We were also aware of the broad nature of the term “mobilization” during the research process. In the ‘Discussion’ section of the manuscript, we mentioned that the mobilization techniques included in this study encompass not only joint mobilization but also neural mobilization, soft tissue mobilization, MWM, and DHC.

Following the editor's advice, we will create an ‘Operational Definitions of Intervention’ section to provide clear operational definitions for each of these mobilization techniques in the ‘Methods’ section of the revised manuscript.

However, a subgroup meta-analysis based on the type of technique was not statistically feasible due to the very small number of studies using each specific technique (e.g., only one study for DHC and seven studies for MWM). We will explicitly state this in the ‘Limitations’ section and will strongly propose the need for additional RCTs that directly compare the effects of individual mobilization techniques in the 'Suggestions for further research' section.

[Methods] - [Operational Definitions of Interventions]

In this study, 'mobilization' was used as an umbrella term for various manual therapy techniques applied to the shoulder joint and its surrounding structures. The primary techniques identified in the included studies are defined as follows. Joint mobilization refers to low-velocity, passive movements applied by a therapist to a patient's joint to restore mobility and reduce pain, focusing on physiological and accessory motions. Neuromobilization involves the application of mechanical forces to the nervous system through specific body movements to restore neural function. Soft tissue mobilization is a technique that applies mechanical stimulation to soft tissues like muscles and fascia to break down adhesions and relieve restrictions. More specific methods included Mobilization with Movement (MWM), a technique combining a sustained accessory joint glide by the therapist with the patient's active physiological movement, and Dynamic Humeral Centering (DHC), a manual technique aimed at correcting the position of the humeral head within the glenoid fossa during movement to reduce subacromial impingement.

[Limitations]

This systematic review was conducted to determine whether the use of mobilization to improve SIS symptoms can achieve significant clinical benefits. However, this study had several limitations. ~~First, there was high heterogeneity of intervention methods in the studies included in this review. The literature search found only three studies [24, 32, 36] that included mobilization alone as an intervention, which is a small number. Therefore, we widened the scope of the mobilization group and did not limit the types of mobilization. Several differences existed among the studies, such as variations in the frequency of each treatment per week, the total duration of the study in weeks, and the type of physical therapy used, making it difficult to determine the most clinically beneficial technique.~~

→ First, this review was limited by the high heterogeneity of intervention methods in the studies included in this review. Since the literature search found only three studies [26, 34, 38] that included mobilization alone as an intervention, which is a small number. Therefore, we widened the scope of the mobilization group to include a broad range of mobilization techniques. However, this diversity—which also included variations in treatment frequency, duration, and the type of physical therapy used as co-interventions—made a subgroup meta-analysis based on specific technique types unfeasible. The number of studies for any single technique was too small for a meaningful statistical comparison (e.g., only one study for DHC and seven for MWM, which were often combined with other therapies). This limitation ultimately prevents us from drawing conclusions about the comparative effectiveness of different mobilization approaches and makes it difficult to determine the most clinically beneficial technique.

[Suggestions for further research]

... Finally, although this study successfully synthesized and utilized the broad concept of mobilization, allowing for an assessment of its effectiveness as a category of therapeutic interventions in improving SIS, it did not facilitate detailed comparisons between individual techniques. ~~Consequently, determining the most effective technique in real-world practice remains challenging. Therefore, more RCTs specifying each technique and more systematic reviews are needed.~~

→ Consequently, determining the most effective technique in real-world practice remains challenging. Future research should prioritize high-quality randomized controlled trials (RCTs) that directly compare the effectiveness of individual, well-defined mobilization techniques against each other (e.g., MWM versus glenohumeral joint mobilization) and against credible sham controls. This will help identify the most beneficial approach for patients with SIS.

Point 3: Risk of Bias and Methodological Quality: A significant proportion of the included studies are at high risk of bias, particularly in blinding and outcome assessment. This should be discussed more explicitly as a limitation.

Response 3: We agree with this assessment. Using the RoB 2.0 tool, we evaluated that 12 out of the 19 included studies had a 'high risk of bias'. Specifically, we identified frequent issues arising from the difficulty of blinding participants and personnel due to the nature of the intervention.

In the revised manuscript, we will address this issue more explicitly and in greater depth within the 'Limitations' section. We will specifically discuss the impact that the lack of reliable blinding has on subjective outcome measures such as pain (VAS), and we will clearly state the possibility that the treatment effects may have been overestimated as a result. We will also clearly explain how these methodological flaws in a majority of the included studies weaken the level of evidence of this systematic review and why caution is needed in interpreting the results.

[Limitations]

... Therefore, although a large number of studies presented the AROM as an index, the number of studies that could be used for each movement-specific analysis was small. ~~Finally, the majority of studies were assessed as having “some concern” and “high risk” of bias due to failures in randomization and reliable blinding and the omission and correction of accurate outcomes, resulting in a high risk of bias.~~

→ Finally, the strength of our evidence is limited by significant methodological flaws in the included literature. In our assessment, a substantial number of studies—12 out of 19—were assessed as having a “high risk” of bias, primarily due to failures in randomization and reliable blinding and the omission and correction of accurate outcomes, resulting in a high risk of bias.

This fact could have led to an overestimation of the treatment effect, particularly for subjective outcomes like pain scores (VAS). Therefore, despite the positive findings presented here, the results must be interpreted with significant caution, fully considering this potential for bias.

Point 4: Limitations and Future Directions: The discussion section should better acknowledge the methodological limitations of the included studies and propose directions for future high-quality research.

Response 4: We agree with your point. As you suggested, we will strengthen both of these sections. In the 'Limitations' section, we will add more depth by including a discussion on how each of the currently described limitations—high heterogeneity, risk of bias, varied follow-up periods, and small sample sizes—specifically impacts the interpretation and generalizability of our findings.

In the 'Suggestions for further research' section, we will propose more concrete and actionable directions. For example, instead of simply suggesting 'the introduction of new blinding methods,' we will propose specific strategies already mentioned in the body of the text, such as applying research methods that hide the ultimate purpose of the study from participants or blind them to the intervention being applied to other groups. Furthermore, to directly address the key limitations identified in this study, we will clearly propose the need for large-scale RCTs that compare specific, clearly defined mobilization techniques with a reliable sham intervention, include long-term follow-up to assess the durability of effects, and ensure blinding of outcome assessors.

[Limitations] (The revisions in the Limitations section related to Point 4 have been incorporated in conjunction with our response to Points 2 and 3.)

[Suggestions for further research]

... First, the separation of mobilization from other interventions is necessary to obtain a clearer independent treatment effect of mobilization in RCTs.

... Several studies excluded from the review included various interventions, making it difficult to identify the independent effects of mobilization itself. Therefore, future studies should be designed to either evaluate mobilization as an independent intervention or directly compare specific, well-defined techniques under a standardized protocol.

Next, to reduce the risk of bias in the study, ~~establishing a completely randomized setting and ensuring thorough assessment to verify that the omission or correction of results does not introduce distortion to the conclusions are important. In 10 out of the 19 studies, the failure of reliable blinding due to the characteristics of physical therapy resulted in a high risk of bias. Therefore, we suggest the application of research methods to minimize bias, such as concealing the ultimate purpose of the study from subjects or blinding to interventions applied to other groups.~~

→ future studies must employ rigorous methodological designs. This includes establishing a completely randomized setting and ensuring thorough assessment to verify that the omission or correction of results does not introduce distortion to the conclusions are important. A key challenge, highlighted by the high risk of bias in 10 of the 19 studies we reviewed, is the inherent difficulty of reliable blinding die tp the characteristics of physical therapy. To mitigate this, future research should implement specific strategies, such as concealing the ultimate purpose of the study from subjects or blinding to interventions applied to other groups. Also, to directly address the major limitations identified in this study, large-scale RCTs are required. These trials should compare specific, clearly defined mobilization techniques with a reliable sham intervention and include long-term follow-up to evaluate the durability of the effects. Critically, ensuring the thorough blinding of outcome assessors is also essential to maintain objectivity.

... Consequently, determining the most effective technique in real-world practice remains challenging. ~~Therefore, more RCTs specifying each technique and more systematic reviews are needed.~~

→ Future research should prioritize high-quality randomized controlled trials (RCTs) that directly compare the effectiveness of individual, well-defined mobilization techniques against each other (e.g., MWM versus glenohumeral joint mobilization) and against credible sham controls. This will help identify the most beneficial approach for patients with SIS.

**[Reviewer #1]**

Point 1: The keywords are missing in abstract.

Response 1: Thank you for your suggestion. In accordance with the journal's author guidelines, we did not include a separate keywords section in the 'manuscript' file. The keywords were instead submitted during the paper submission process. The keywords are as follows: joint mobilization; shoulder impingement syndrome; systematic review; Meta-analysis

**[Reviewer #2]**

Point 1: Suggest to also add statements on exclusion and exclusion with relation to year of publication and language . Have you accepted all primary and secondary SIS, patients with previous surgeries, previous fractures etc..

Response 1: Thank you for this valuable point. While the 'Database selection and search' section specifies that our search included studies up to April 2023, the language criteria were ambiguous. We will clarify in this section that only articles published in English were included.

The studies used in this systematic review were selected based on our predefined PICOS framework. The 'P' (Participants) was defined as "Patients with SIS," and we did not exclude participants based on the type of SIS or their previous medical history, such as prior surgeries or fractures. We will explicitly mention this in the final sentence of the 'Inclusion and exclusion criteria' section.

[Database selection and search]

PubMed (www.pubmed.com), Embase (www.embase.com), and the Cochrane Library (www.thecochranelibrary.com) databases were searched for English-language clinical studies applying mobilization to SIS, including all articles published from the inception of the databases through April 2023.

**[Inclusion and exclusion criteria]**

In the retrieved articles, no restrcictions were ~~placed on subject age, sex, composition of the control group, treatment duration, follow-up period, or indices used in the study.~~

→ applied regarding participants (age, sex), interventions (types or techniques of mobilization), comparisons (composition of the control group), outcomes (indices used for assessment), or study characteristics (treatment duration, follow-up period, or study design) in the retrieved articles. Additionally, we did not exclude participants based on the type of SIS (primary or secondary) or their medical history (e.g., previous surgeries or fractures).

**[Reviewer #3]**

**# Abstract- keywords**

Point 1: Include important information about the method that was used in this systematic review and meta-analyses – PRISMA.

Response 1: Thank you for your suggestion. We will revise the 'Methods' section of the abstract to state that this study was conducted in accordance with the PRISMA guidelines. Currently, this is mentioned only on line 81 of the main text.

[Abstract]

Methods: PubMed, Embase and the Cochrane Library were searched for studies published up to April 2023. Randomized controlled trials that evaluated the effectiveness of mobilization for SIS were included. Literature was extracted using Endnote 20, and the risk of bias was assessed using the Cochrane risk-of-bias tool 2.0. A meta-analysis was conducted using RevMan 5.4. This study was conducted in accordance with the PRISMA guidelines.

Point 2: In the conclusion of the abstract, the study's limitations are highlighted: „Owing to the clinical heterogeneity, uncertain risk of bias, and small number of studies, high-quality studies evaluating the effectiveness of mobilization are warranted”. Given the significant limitations mentioned, I suggest reformulating the approach to provide a clearer direction for future research.

Response 2: This is a very important point. We agree that the current conclusion of the abstract is somewhat general. Based on the discussion in the 'Suggestions for further research' section of the main text, we will revise the abstract's conclusion to be more specific. The revised conclusion will clearly state the need for high-quality studies that verify the effect of mobilization in isolation from other interventions, include larger sample sizes and long-term follow-up, and compare the effects of specific mobilization techniques.

[Abstract]

Conclusion: Mobilization may be effective in improving the clinical symptoms of SIS and may be recommended in addition to conventional treatments~~. Owing to the clinical heterogeneity, uncertain risk of bias, and small number of studies, high-quality studies evaluating the effectiveness of mobilization are warranted.~~

→ Future studies should focus on evaluating the effects of mobilization as a stand-alone intervention, incorporate larger sample sizes and long-term follow-up, and conduct high-quality comparative trials to identify the most effective specific mobilization techniques.

**# Introduction**

Point 3: Since the sham mobilization was used as a comparison in row 34, I suggest inserting information about this in the Introduction section. It is a new concept, and adding information in the Introduction section will help emphasize its relevance.

Response 3: Thank you for the suggestion. We will add an explanation of 'sham mobilization' to the Introduction section. We will explain that sham mobilization is an important comparative intervention in manual therapy research used to control for non-specific effects such as therapist contact and attention. We will also emphasize its concept and relevance to aid the reader's understanding.

[Introduction]

... These mobilizations can be applied to patients with SIS to improve shoulder joint pain and restore joint function. However, to determine whether these therapeutic effects are truly attributable to the mobilization itself, the use of sham mobilization as a comparison intervention is particularly important, as it helps control for placebo effects such as therapist attention and physical contact in manual therapy studies. Introducing sham mobilization allows for a more accurate assessment of the true therapeutic effects of mobilization techniques.

**# Methods**

Point 4: Please provide in this section the link for Data Availability
Response 4: In response to your suggestion, we will add a 'Data Availability Statement' section at the end of the Methods section of the manuscript. We will specify that all data used in this study were extracted from the published RCTs included in our analysis and that all relevant data are contained within the main text and supplementary information file.

[Methods] - [Data Availability Statement]

All data for this study were extracted from the published randomized controlled trials (RCTs). The relevant data can be found in both the main body of the paper and the supplementary information file.

Point 5: Please provide a reference for the Cochrane Handbook for Systematic Reviews of Interventions. And also for the PRISMA guideline (rows 80-81).

Response 5: Thank you for this valuable point. While the Cochrane Handbook and PRISMA guidelines are currently mentioned in the main text, they were not formally cited. We will add the official citations for these documents to the Methods section and include them in the reference list. Consequently, the citation numbers within the manuscript have shifted, which we have corrected throughout the text.

[References]

20. Higgins J, Thomas J, Chandler J, Cumpston M, Li T, Page MJ, Welch VA. Cochrane Handbook for Systematic Reviews of Interventions Version 6.4. Cochrane, 2023.

21. Page MJ, McKenzie JE, Bossuyt PM, Boutron I, Hoffmann TC, Mulrow CD, et al. The PRISMA 2020 statement: an updated guideline for reporting systematic reviews. BMJ 2021;372:n71.

**# Database selection and search**

Point 6: For selecting relevant keywords, it was preferred to use an automated tool, such as MeSH or the Word Frequency Analyzer Tool.

Response 6: We did not use automated tools such as MeSH in this study. Instead, we established a comprehensive search strategy through extensive discussions among our experienced research team and by reviewing the keywords used in existing high-quality systematic reviews on this topic. We appreciate the reviewer's suggestion and will actively consider using such tools in future research to enhance both search efficiency and comprehensiveness.

Point 7: Please explain why the searching strategy was used: „mobilization” and „mobilization” – row 91?

Response 7: This is a clear typo. The term "mobilization" was duplicated and should have been written as "mobilization" and "impingement." We will correct this in the revised manuscript.

**# Inclusion and exclusion criteria**

Point 8: Please improve this section to add clarity and flow.

Response 8: We agree. We will improve the readability of this section by using lists or subheadings to clearly distinguish between the inclusion and exclusion criteria, and we will reorganize the content to make it easier for readers to understand.

Point 9: It is essential to include the abbreviations for participants, interventions, comparisons, outcomes, and study designs that refer to PICOS- rows 95-96.

Point 10: Additionally, since PICOS was used, the exclusion criteria need to follow the structure to ensure methodological consistency.

Response 9, 10: This is an invaluable suggestion. To enhance methodological clarity, we will restructure the inclusion and exclusion criteria section according to the PICOS framework. We will explicitly delineate each element—Participants, Intervention, Comparison, Outcomes, and Study Designs. For example, under the 'Study Designs' element, we will specify the exclusion of non-randomized controlled trials (non-RCTs) to ensure methodological consistency.

[Inclusion and exclusion criteria] - [Inclusion criteria]

Articles included in this systematic review were selected based on the participants, interventions, comparisons, outcomes, and study designs framework.

(1) Participants(P): Patients with SIS (2) Interventions(I): The experimental group received the mobilization therapy alone or other interventions in addition to the mobilization. (3) Control(C): The control group received treatments other than the mobilization. If the experimental group has additional interventions beyond the mobilization, the control group has no interventions other than the same intervention. (4) Outcome(O): Diagnostic metrics in SIS, including VAS, ROM, DASH, SPADI, constant score, and more. (5) Study design(S): Only randomized controlled trials were included.

~~Exclusion criteria were as follows: (1) Non-randomized controlled trials, retrospective analyses, or case reports. (2)Articles with no available data or duplicate date.~~

→ [Exclusion criteria]

Studies were excluded if they met any of the following criteria:

(1) Participants(P): The study population did not consist of individuals diagnosed with SIS. (2) Interventions(I): The mobilization group received additional concurrent interventions that could not be isolated from mobilization. (3) Control(C): The comparison group received interventions overlapping with mobilization that could not be separated, or no appropriate control group was provided. (4) Outcome(O): The study did not report relevant clinical outcomes related to SIS, such as pain, range of motion, or functional measures. (5) Study design(S): Non-randomized controlled trials (non-RCTs), observational studies, case reports, and other study designs that did not meet the methodological requirements for RCT classification.

In the retrieved articles, no restrictions were ~~placed on subject age, sex, composition of the control group, treatment duration, follow-up period, or indices used in the study.~~

→ applied regarding participants (age, sex), interventions (types or techniques of mobilization), comparisons (composition of the control group), outcomes (indices used for assessment), or study characteristics (treatment duration, follow-up period, or study design) in the retrieved articles. Additionally, we did not exclude participants based on the type of SIS (primary or secondary) or their medical history (e.g., previous surgeries or fractures).

**# Literature selection and analysis**

Point 11: Please remove the duplicate of the subtitle – rows 106- 107

Response 11: Thank you for your careful observation. We have identified the duplicate subheading on page 5, line 98 of the manuscript. We will delete this section during the revision.

Point 12: Please mention where the selected articles were exported by the two researchers (GL and WH)- 112-113. In the abstract, it is noted that EndNote 20 was used.

Response 12: Thank you for your valuable point. While the use of EndNote 20 was mentioned in the abstract, it was omitted from the methodology section of the main text. We will specify in the 'Literature selection and analysis' section that all retrieved literature was exported to the EndNote 20 program to remove duplicates before screening.

[Literature selection and analysis]

All retrieved references were exported to EndNote 20, where duplicate records were removed before screening. Literature search and selection were independently conducted by two researchers (GL and WH).

Point 13: Before applying Rob 2.0 to identify the risk of bias, a tool was used to screen and select studies? This step is crucial to ensure that only eligible studies are included in the meta-analysis, especially through the peer-blinding process. E.g., Ryyan?

Response 13: We did not use a specialized screening tool like Rayyan for this study. The literature selection process was conducted manually after removing duplicates in EndNote 20, with two researchers independently reviewing titles, abstracts, and full texts. We aimed to ensure rigorous screening through this independent review and consensus process. For future research, as the reviewer suggested, we will consider using tools like Rayyan to enhance the efficiency and blinding of the screening process.

**# Discussion**

Point 14: In rows 370-371, it is mentioned that the meta-analysis was performed on 11 studies out of the 19. Please argue why the meta-analysis was made on 11 studies and why it isn’t mentioned in the Method section.

Response 14: Thank you for your valuable feedback. The meta-analysis was limited to 11 studies because only these 11 studies provided quantitatively z data (mean, standard deviation, and sample size) for similar interventions and control groups. We apologize for our oversight in not explicitly stating this criteria in the 'Methods' section. We will revise the manuscript to include a sub-section such as 'Data consolidation and analysis' within the 'Methods' section to clearly describe the criteria for inclusion in the meta-analysis.

[Methods] - [Data consolidation and analysis]

For a quantitative synthesis of the results, a meta-analysis was conducted. Inclusion in the meta-analysis was limited to studies that provide sufficient statistical data (specifically means, standard deviations, and sample sizes) for clinically comparable intervention and control groups.

Point 15: Regarding some indices, a high level of heterogeneity was noted in the Result section (eg, rows 192, 195, 200, 205, 211), which could affect the ability to draw firm conclusions about the effectiveness of joint mobilization (rows 372, 273).

Response 15: We agree with the reviewer's valuable comment and will strengthen this section. We will expand our discussion of statistical heterogeneity in the Discussion section. Specifically, we will elaborate on how clinical diversity among the included studies may have contributed to the observed statistical heterogeneity. This includes differences in: various mobilization techniques (e.g., neural mobilization, soft tissue mobilization, and MWM), applied concurrent interventions (e.g., different types of exercise therapy), diverse control groups, and variations in treatment dosage (e.g., frequency and duration). By doing so, we will re-emphasize the need for cautious interpretation of the pooled results.

[Summary of findings]

... The type of intervention varied across studies, necessitating a preliminary categorization for comparison. Categorization was carried out using intervention methods, control groups, and indices. The clinical heterogeneity observed across the included studies—such as variations in manual therapy techniques (e.g., neural mobilization, soft tissue mobilization, MWM), types of concomitant interventions such as exercise therapy, characteristics of control groups, and treatment dosage like frequency and duration may have contributed to the statistical heterogeneity. Therefore, the pooled results should be interpreted with caution. A meta-analysis was performed on 11 out of the 19 studies, and comparisons were made based on the aforementioned categorization.

Point 16: Since the aim of the study was to examine the clinical effectiveness of mobilization for SIS, it is highly suggested to focus on this, not on clinical implications- this subsection could be just an introduction for the summary.

Response 16: We completely agree with your comment. We acknowledge that the current 'Clinical Implications' section is more of a summary, as it details the interventions, control groups, and outcome measures of the included studies rather than discussing the true implications of our findings.

As you suggested, we will restructure the Discussion section. We will create a new section, 'Characteristics of Included Studies,' to be placed before the 'Summary of findings.' The content currently in the 'Clinical Implications' section will be moved here. The 'Clinical Implications' section will then be revised to focus specifically on the meaning of our research findings for decision-making in real-world clinical practice.

[Clinical implications]

~~Relocate the existing content to the 'Characteristics of Included Studies' section.~~

→ The findings of this systematic review offer several clinical implications for clinicians managing patients with SIS. First, mobilization is an effective and safe adjunct to standard treatment protocols of SIS. To accelerate or enhance pain relief and functional restoration, clinicians should consider incorporating mobilization alongside conventional exercise therapy. Second, a patient-centered and individualized approach based on the patient's condition is crucial. The finding that mobilization was not superior to exercise therapy suggests that clinicians can select the intervention based on the patient's primary problem. For instance, in patients with acute pain or significant limitations in active movement, mobilization, as a passive approach, may be a more suitable initial intervention. Conversely, a well-structured exercise program may be equally effective for patients whose primary issue is muscle weakness or who are capable of active participation. Third, the current evidence is insufficient to determine the superiority of any specific mobilization technique. The included studies employed a range of techniques, but there is a lack of high-quality research directly comparing their efficacy. Consequently, until more definitive evidence is available, the selection of a specific technique (e.g., MWM, joint mobilization) should be guided by a clinician’s expert and the individual patient's presentation.

**# General recommendation:**

Point 17: While the topic is relevant and valuable for the physical therapy area, several methodological aspects require clarification; please see the aforementioned points.

Response 17: We will fully incorporate all the previously mentioned methodological points into the revised manuscript to improve the clarity and integrity of our research.

Point 18: Strongly recommend keeping “joint mobilization” and not “mobilization”, as this is the term mentioned in the title and abstract as well.

Response 18: Thank you for your valuable point regarding the consistency of our terminology. We would like to explain our intention. While we used the most representative technique, "joint mobilization," in the title, our actual inclusion criteria were for the broader concept of "mobilization," which includes techniques like neural mobilization and soft tissue mobilization. This approach was intended to comprehensively review all manual techniques applied to shoulder impingement syndrome.

In response to your suggestion, we will clearly distinguish the use of the terms "joint mobilization" and "mobilization" throughout the entire manuscript. Specifically, we will use "joint mobilization" to refer to a particular joint technique and "mobilization" when discussing the overarching concept that encompasses various techniques, to prevent any confusion for the reader.

* We have additional corrections.

[1] We have corrected the incorrectly listed names in the 'Author contributions' section, as detailed below.

(1) W Jun -> H Woo

(2) BH Jeon -> SJ Jeon

[2] In addition, we would like to inform you that the corresponding author for this manuscript has been changed from ‘Wonbae Ha & Junghan Lee’ to ‘Wonbae Ha & Seojae Jeon’.

Again, thank you for giving us the opportunity to strengthen our manuscript with your valuable comments. We have worked hard to incorporate your feedback and hope that these revisions persuade you to accept our submission.

Sincerely,

Gayeong Lee, and Wonbae Ha
