# Supplementary material for: Clinical efficacy of joint mobilization for shoulder impingement syndrome: a systematic review and meta-analysis
Source: PLoS One. 2026 Jul 13;21(7):e0352260. doi: 10.1371/journal.pone.0352260 (PMC13362101; doi:10.1371/journal.pone.0352260)
Supplement: Attachment — Submitted filename: Response to Reviewers_R2.docx [file pone.0352260.s005.docx]

Dear Dr. Saumya Srivastava, Academic Editor, PLOS ONE

Thank you for the opportunity to submit a revised version of our manuscript, "Clinical efficacy of joint mobilization for shoulder impingement syndrome: a systematic review and meta-analysis" (PONE-D-24-07775R2). We are grateful to you and the reviewers for the constructive feedback, which has further improved the quality and clarity of our manuscript.

**[Academic editor]**

Point 1: Clarify and emphasize the conceptual and clinical heterogeneity of the various “mobilization” techniques included.

**Response:** Thank you for this valuable feedback. We have revised the "Operational Definitions of Interventions" in the Methods section to more explicitly emphasize the conceptual and clinical heterogeneity of the various mobilization techniques included in our study (e.g., joint mobilization, neuromobilization, soft tissue mobilization, and MWM).

[Method] - [Operational Definitions of Interventions]

… More specific methods included Mobilization with Movement (MWM), a technique combining a sustained accessory joint glide by the therapist with the patient's active physiological movement, and Dynamic Humeral Centering (DHC), a manual technique aimed at correcting the position of the humeral head within the glenoid fossa during movement to reduce subacromial impingement.

→ … More specific methods included Mobilization with Movement (MWM), a technique combining a sustained accessory joint glide by the therapist with the patient's active physiological movement, and Dynamic Humeral Centering (DHC), a manual technique aimed at correcting the position of the humeral head within the glenoid fossa during movement to reduce subacromial impingement. These techniques, while sharing the overarching goal of restoring mobility, operate through distinct biomechanical and neurophysiological mechanisms, contributing to conceptual heterogeneity.

Point 2: More explicitly discuss the implications of high heterogeneity observed in several meta-analyses.

**Response:** We agree with your assessment. In the Discussion section, we have explicitly expanded upon the implications of the high heterogeneity (e.g., I² > 90%) observed in several of our meta-analyses. We added language cautioning readers that these pooled estimates should be interpreted carefully, as the high statistical heterogeneity is likely a direct result of the diverse clinical protocols, techniques, and co-interventions used across the included trials.

[Discussion] – [Summary of findings]

… The results of this meta-analysis should be interpreted with caution due to significant statistical heterogeneity, which primarily stems from the considerable clinical diversity among the included studies. This diversity was evident in several areas …

→ … The results of this meta-analysis should be interpreted with caution due to significant statistical heterogeneity, which primarily stems from the considerable clinical diversity among the included studies. Specifically, the high I² values (frequently >90%) observed in several meta-analyses compromise the precision of the pooled estimates. This indicates that the observed effects are highly variable and context-dependent, directly reflecting the diverse nature of 'mobilization' in clinical practice. This diversity was evident in several areas …

Point 3: Temper the conclusions to reflect the adjunctive and primarily short-term nature of the observed effects.

Response: We have appropriately tempered the conclusions in both the Abstract and the main Conclusion sections. We have revised the text to clearly state that joint mobilization should be considered an adjunctive treatment to conventional physical therapy, and we emphasized that the observed benefits are primarily short-term, reflecting the limitations of the current evidence base.

[Abstract]

Conclusion: Mobilization may be effective in improving the clinical symptoms of SIS and may be recommended in addition to conventional treatments

→ Conclusion: Mobilization may offer adjunctive, short-term benefits in improving the clinical symptoms of SIS when added to conventional treatments.

[Conclusion]

Therefore, clinically, mobilization may be more advisable when patients have weak muscle strength or limited AROM or when an acute injury prevents active movement. However, the quality of the evidence was not high, owing to the high heterogeneity among the included studies and the inclusion of a large number of studies with a high risk of bias assessment.

→ Therefore, clinically, mobilization may be more advisable when patients have weak muscle strength or limited AROM or when an acute injury prevents active movement. These benefits appear to be primarily short-term and adjunctive in nature. However, the quality of the evidence was not high, owing to the high heterogeneity among the included studies and the inclusion of a large number of studies with a high risk of bias assessment.

**[Reviewer #4]**

Point 1: In the Results section of the abstract, the term physiotherapy is used to refer to physical therapy. Please use consistent terminology throughout the abstract and manuscript.

Response: We apologize for the inconsistency. We have changed "physiotherapy" to "physical therapy" in the Results section of the Abstract, ensuring uniform terminology throughout the entire manuscript.

[Abstract]

Results: Nineteen randomized controlled trials (956 participants) were selected based on the inclusion criteria. Adding mobilization to traditional physiotherapy reduced pain ...

→ Results: Nineteen randomized controlled trials (956 participants) were selected based on the inclusion criteria. Adding mobilization to traditional physical therapy reduced pain …

Point 2: (Lines 70–74) This paragraph introduces the importance of sham mobilization before sham mobilization is clearly defined earlier in the manuscript. Please revise this paragraph for clarity by first defining sham mobilization in clear terms

Response: Following your suggestion, we have inserted a clear operational definition of sham mobilization in the Introduction prior to discussing its importance, improving the clarity of this paragraph.

[Introduction]

These mobilizations can be applied to patients with SIS to improve shoulder joint pain and restore joint function. However, to determine …

→ These mobilizations can be applied to patients with SIS to improve shoulder joint pain and restore joint function. In research settings, sham mobilization is utilized as a placebo control, involving manual contact by the therapist without delivering the actual therapeutic force or specific joint movement. However, to determine …

Point 3: (Line 79) The statement that “no studies have reported the effectiveness of mobilization for SIS” is unclear. If the intention is to state that no prior systematic reviews have evaluated this question, please revise and re-write.

Response: Thank you for pointing this out. We agree that the final sentence was unclear and potentially misleading. Since we already explicitly stated earlier in the same paragraph that "no systematic review has examined the effects of mobilization as a whole on SIS," the final sentence was redundant. To avoid any confusion and improve the flow of the text, we have completely deleted the problematic sentence ("However, no studies have reported the effectiveness of mobilization for SIS.") from the revised manuscript.

[Introduction]

Furthermore, studies have reported the effectiveness of mobilization for various conditions, including ankle sprains [13], ankle joint instability [14], knee osteoarthritis [15], carpal tunnel syndrome [16], and frozen shoulder [17, 18]. However, no studies have reported the effectiveness of mobilization for SIS.

→ Furthermore, studies have reported the effectiveness of mobilization for various conditions, including ankle sprains [13], ankle joint instability [14], knee osteoarthritis [15], carpal tunnel syndrome [16], and frozen shoulder [17, 18]. ~~However, no studies have reported the effectiveness of mobilization for SIS.~~

Point 4: (Line 79) Classification of Physical Therapy: Please provide a brief rationale for grouping different physical therapy modalities under the single category of physical therapy in the analysis. You need to show that it can be combined!

Response: We have added a brief rationale in the Methods section explaining our classification. We justified grouping these modalities under a single "physical therapy" category because they share common physiological goals (e.g., pain relief, reducing inflammation) and represent standard conventional care that is routinely combined with manual therapy in real-world clinical settings.

[Method] – [Operational Definitions of Interventions]

… a manual technique aimed at correcting the position of the humeral head within the glenoid fossa during movement to reduce subacromial impingement. These techniques, while sharing the overarching goal of restoring mobility, operate through distinct biomechanical and neurophysiological mechanisms, contributing to conceptual heterogeneity.

→ … a manual technique aimed at correcting the position of the humeral head within the glenoid fossa during movement to reduce subacromial impingement. These techniques, while sharing the overarching goal of restoring mobility, operate through distinct biomechanical and neurophysiological mechanisms, contributing to conceptual heterogeneity. Various modalities such as TENS, ultrasound, and heat therapy were grouped under a single 'physical therapy' category for analysis, as they share the common physiological goals of standard conservative care (e.g., pain relief, tissue healing) and are routinely administered together in clinical settings.

Point 5: (Line 89) The inclusion of database URLs in parentheses is unnecessary and can be removed.

Response: The database URLs in parentheses have been removed.

[Method] – [Database selection and search]

PubMed (www.pubmed.com), Embase (www.embase.com), and the Cochrane Library (www.thecochranelibrary.com) databases were searched

→ PubMed ~~(www.pubmed.com)~~, Embase ~~(www.embase.com)~~, and the Cochrane Library ~~(www.thecochranelibrary.com)~~ databases were searched

Point 6: (Line 93) The term “mobilization” appears to be repeated; please correct for clarity.

Response: We have corrected the repetition of the term "mobilization".

[Method] – [Database selection and search]

These English-language online databases were searched using a combination of the following search terms: “shoulder impingement syndrome,” “rotator cuff,” “impingement,” “subacromial impingement,” “mobilization,” and “mobilization,” with search formulas modified to each database.

→ These English-language online databases were searched using a combination of the following search terms: “shoulder impingement syndrome,” “rotator cuff,” “impingement,” “subacromial impingement,” ~~“mobilization,”~~ and “mobilization,” with search formulas modified to each database.

Point 7: (Line 109) The phrase “received interventions overlapping with mobilization that could not be separated” is unclear, particularly since studies including mobilization plus other interventions were allowed under the inclusion criteria. Please clarify what is meant by “overlapping” and how “could not be separated” was operationally defined.

Response: We have clarified this phrase. We operationally defined "overlapping and could not be separated" to mean instances where the comparison group received a complex manual therapy package that included elements of joint manipulation, making it impossible to isolate the specific effect of the experimental mobilization.

[Method] – [Exclusion criteria]

(3) Control (C): The comparison group received interventions overlapping with mobilization that could not be separated, or no appropriate control group was provided.

→ (3) Control (C): The comparison group received interventions that overlapped with mobilization that could not be separated (e.g., complex manual therapy packages where the specific effects of mobilization could not be isolated), or no appropriate control group was provided.

Point 8: (Line 116) The phrase “or study design” may be misleading, as only randomized controlled trials were included. Please revise to reflect that study design was, in fact, restricted to RCTs.

Response: We have revised this sentence to remove the misleading phrase "or study design." The text now explicitly states that the study design was strictly restricted to RCTs.

[Method] – [Exclusion criteria]

In the retrieved articles, no restrictions were applied regarding participants (age, sex), interventions (types or techniques of mobilization), comparisons (composition of the control group), outcomes (indices used for assessment), or study characteristics (treatment duration, follow-up period, or study design) in the retrieved articles.

→ In the retrieved articles, no restrictions were applied regarding participants (age, sex), interventions (types or techniques of mobilization), comparisons (composition of the control group), outcomes (indices used for assessment), or study characteristics (treatment duration, follow-up period~~, or study design~~) in the retrieved articles.

Point 9: The Results section provides detailed descriptions of individual trials prior to presenting pooled estimates. Consider condensing trial-level narrative and placing greater emphasis on meta-analytic results, as study-level details are already summarized in tables.

Response: We appreciate this constructive feedback. We have condensed the trial-level descriptive narratives in the Results section, as these details are already provided in the tables, and placed greater emphasis on reporting the meta-analytic results.

[Result] – [Treatment effects]

A between-group analysis was performed in eight studies comparing mobilization plus specific physical therapy with specific physical therapy alone [9, 10, 19, 28-30, 32, 33]. For the sake of simplicity, we will refer to the group that added physical therapy to mobilization as the add-on group and the group that performed physical therapy alone as the PT group in this section. Akhtar et al. [10], who analyzed the VAS and UCLA rating score, and Menek et al. [32], who analyzed the VAS, AROM, DASH, and SF-36, both reported that the add-on group was more effective than the PT group. Conroy and Hayes [19] and Neelapala et al. [33] reported that the add-on group was more effective in the VAS, but no statistically significant difference was found between the two groups in the AROM. Neelapala et al. [33] also found a significant effect of the add-on group on muscle strength. Eliason et al. [9] showed that pain improvement in flexion was greater in the add-on group in the VAS. However, for abduction, external rotation, and internal rotation, the improvement was greater in the PT group, and in the Constant-Murley score, no statistically significant difference was found between the two groups. İğrek and Çolak [29] reported that the add-on group was more effective in some indicators of VAS, DASH, Constant-Murley score, AROM, and muscle strength. Gutiérrez-Espinoza et al. [28], who analyzed the DASH, Constant-Murley score, VAS, and scapular UR, and Kachingwe et al. [30], who analyzed the VAS, AROM, and SPADI, found no statistically significant differences between the two groups.

→ A between-group analysis was performed in eight studies comparing mobilization plus specific physical therapy with specific physical therapy alone [9, 10, 19, 28-30, 32, 33]. ~~For the sake of simplicity, we will refer to the group that added physical therapy to mobilization as the add-on group and the group that performed physical therapy alone as the PT group in this section.~~ For simplicity, in this section we refer to the group that received mobilization in addition to physical therapy as the add-on group, and the group that received physical therapy alone as the PT group. Across these trials, several studies reported greater improvements in pain, function, and, in some cases, muscle strength in the add-on group, whereas others found no statistically significant differences between the add-on and PT groups on key outcomes such as pain, ROM, and shoulder-specific disability measures. ~~Akhtar et al. [10], who analyzed the VAS and UCLA rating score, and Menek et al. [32], who analyzed the VAS, AROM, DASH, and SF-36, both reported that the add-on group was more effective than the PT group. Conroy and Hayes [19] and Neelapala et al. [33] reported that the add-on group was more effective in the VAS, but no statistically significant difference was found between the two groups in the AROM. Neelapala et al. [33] also found a significant effect of the add-on group on muscle strength. Eliason et al. [9] showed that pain improvement in flexion was greater in the add-on group in the VAS. However, for abduction, external rotation, and internal rotation, the improvement was greater in the PT group, and in the Constant-Murley score, no statistically significant difference was found between the two groups. İğrek and Çolak [29] reported that the add-on group was more effective in some indicators of VAS, DASH, Constant-Murley score, AROM, and muscle strength. Gutiérrez-Espinoza et al. [28], who analyzed the DASH, Constant-Murley score, VAS, and scapular UR, and Kachingwe et al. [30], who analyzed the VAS, AROM, and SPADI, found no statistically significant differences between the two groups.~~

Point 10: (Figure 8) One study is labeled as “not estimable.” Please explicitly state the reason (e.g., insufficient variance information or inability to calculate a standard error for the inverse-variance model) in the Results.

Response: We have explicitly stated in the Results section that the Surenkok et al. study was labeled as "not estimable" because it did not provide sufficient variance information to calculate a standard error for the inverse-variance model.

[Result] – [Comparisons of mobilization and sham mobilization]

A low level of heterogeneity was observed between the studies (Fig 8).

→ A low level of heterogeneity was observed between the studies. One study [38] was labeled as 'not estimable' in the analysis due to insufficient variance information to calculate a standard error for the inverse-variance model (Fig 8).

**[Reviewer #5]**

Point 1: (Abstract) Temper conclusion with cautionary language

Response: We have appropriately tempered the conclusions in both the Abstract and the main Conclusion sections. We have revised the text to clearly state that joint mobilization should be considered an adjunctive treatment to conventional physical therapy, and we emphasized that the observed benefits are primarily short-term, reflecting the limitations of the current evidence base.

[Abstract]

Conclusion: Mobilization may be effective in improving the clinical symptoms of SIS and may be recommended in addition to conventional treatments

→ Conclusion: Mobilization may offer adjunctive, short-term benefits in improving the clinical symptoms of SIS when added to conventional treatments.

[Conclusion]

Therefore, clinically, mobilization may be more advisable when patients have weak muscle strength or limited AROM or when an acute injury prevents active movement. However, the quality of the evidence was not high, owing to the high heterogeneity among the included studies and the inclusion of a large number of studies with a high risk of bias assessment.

→ Therefore, clinically, mobilization may be more advisable when patients have weak muscle strength or limited AROM or when an acute injury prevents active movement. These benefits appear to be primarily short-term and adjunctive in nature. However, the quality of the evidence was not high, owing to the high heterogeneity among the included studies and the inclusion of a large number of studies with a high risk of bias assessment.

Point 2: (Methods) Justify model choice (please justify your choice of the random-effects model (e.g., “due to anticipated clinical and methodological heterogeneity”)), add publication bias assessment (describe whether funnel plots or Egger’s tests were planned/performed, especially for meta-analyses with ≥10 studies. If not performed, please explain why) and clarify missing data handling (the statement “we used a method that did not include missing data” is vague). Please specify the approach (e.g., complete-case analysis) and whether sensitivity analyses were conducted to assess robustness).

Response:

- Model Choice: We have added a sentence explicitly justifying our choice of the random-effects model, stating that it was selected "due to the anticipated clinical and methodological heterogeneity among the included studies."

- Missing Data: We clarified our handling of missing data by specifying that a "complete-case analysis" approach was used, and we explicitly described the sensitivity analyses conducted to assess robustness.

- Publication Bias: We added a statement explaining that while publication bias assessment (e.g., funnel plots, Egger’s tests) was planned, it was not performed because none of our specific meta-analyses included 10 or more studies, rendering such tests statistically underpowered according to Cochrane guidelines.

[Method] - [Data extraction]

In addition to the I^2^ assessment for heterogeneity, we used a Random-Effects Model, which allows for differences in true effect sizes between studies and is used when there is heterogeneity, and is weighted using the inverse variance method. … Therefore, we used a method that did not include missing data in our analyses, and we also performed sensitivity analyses to assess the robustness of our results.

→ In addition to the I^2^ assessment for heterogeneity, we used a Random-Effects Model due to anticipated clinical and methodological heterogeneity, which allows for differences in true effect sizes between studies and is used when there is heterogeneity, and is weighted using the inverse variance method. … Therefore, we used a complete-case analysis approach that excluded missing data~~method that did not include missing data in our analyses, and we also performed sensitivity analyses to assess the robustness of our results~~. Publication bias assessments (e.g., funnel plots) were not performed as no meta-analysis included 10 or more studies.

Point 3: (Results) Comment on high heterogeneity (when reporting I² values >90%, please add a brief comment on the implications for interpretability (e.g., “Results should be interpreted with caution due to substantial heterogeneity.”), and add sensitivity analysis for high RoB studies.

Response: For all meta-analyses reporting I² values >90%, we have inserted a brief cautionary comment in the Results section (e.g., "These results should be interpreted with caution due to the high level of heterogeneity observed among the studies"). And we originally planned and attempted to perform a sensitivity analysis by excluding studies with a high risk of bias, as you recommended. However, because a significant proportion of the included studies (12 out of 19) were classified as having a "high risk of bias," excluding them left an insufficient number of studies (often only 0 or 1 study) in most of our specific meta-analytic subgroups (such as VAS or AROM comparisons). Consequently, meaningful statistical pooling was not possible for the sensitivity analysis. We have transparently acknowledged this inability to perform the sensitivity analysis as a limitation in the Discussion section, highlighting the urgent need for higher-quality RCTs in this field.

Point 4: (Discussion) Expand limitations (publication bias, multiple testing, subgroup caution)

Response: We have expanded the Limitations subsection in the Discussion to include the potential impact of undetected publication bias, the risks associated with multiple testing across our numerous subgroup analyses, and the need for extreme caution when interpreting the results of subgroups with very small numbers of studies.

[Result]

A high level of heterogeneity was found among the studies.

→ These results should be interpreted with caution due to the high level of heterogeneity observed among the studies.

→ Substantial heterogeneity was observed, indicating that these results should be interpreted with caution.

→ Given the high level of heterogeneity, this finding requires careful interpretation.

→ with a high level of heterogeneity among the studies, which warrants cautious interpretation of the pooled estimate.

[Limitation]

Fourth, the assessment indices used in each study were too diverse to allow for a detailed subgroup analysis. In the case of the AROM, various types of shoulder joint movements were presented in each study. Therefore, although a large number of studies presented the AROM as an index, the number of studies that could be used for each movement-specific analysis was small. Finally, the strength of our evidence is limited by significant methodological flaws in the included literature. In our assessment, a substantial number of studies—12 out of 19—were assessed as having a “high risk” of bias, primarily due to failures in randomization and reliable blinding and the omission and correction of accurate outcomes, resulting in a high risk of bias.

→ Fourth, the assessment indices used in each study were too diverse to allow for a detailed subgroup analysis. For example, although many studies used AROM as an index, various types of shoulder joint movements were measured, leaving a small number of studies for each movement-specific analysis. Consequently, the numerous subgroup analyses introduce the possibility of multiple testing errors, meaning these results must be interpreted cautiously. Furthermore, the risk of undetected publication bias remains a concern. Finally, the overall strength of our evidence is limited by significant methodological flaws in the included literature. A substantial proportion of the studies (12 out of 19) were assessed as having a "high risk" of bias, primarily due to failures in randomization, reliable blinding, and accurate outcome reporting. Because of this high proportion of flawed studies, we were unable to conduct our planned sensitivity analysis; excluding these 12 studies would have left too few trials in each subgroup to perform meaningful meta-analyses. This limitation strongly underscores the critical need for future RCTs with rigorous methodological designs to confirm these findings.

Point 5: (Editorial) Remove duplicate abstract, complete tables, and clarify search date

Response: We have removed the duplicate text in the abstract, verified that all tables are complete, and clarified the exact dates of our database searches in the Methods section.

[Abstract]

Methods: PubMed, Embase and the Cochrane Library were searched for studies published up to April 2023.

→ Methods: PubMed, Embase and the Cochrane Library were searched for studies published up to April 30, 2023.

[Method] – [Database selection and search]

databases were searched for English-language clinical studies applying mobilization to SIS, including all articles published from the inception of the databases through April 2023.

→ databases were searched for English-language clinical studies applying mobilization to SIS, including all articles published from the inception of the databases through April 30, 2023

Again, thank you for giving us the opportunity to strengthen our manuscript with your valuable comments. We have worked hard to incorporate your feedback and hope that these revisions persuade you to accept our submission.

Sincerely,

Gayeong Lee, and Wonbae Ha
