# Supplementary material for: Clinical efficacy of joint mobilization for shoulder impingement syndrome: a systematic review and meta-analysis
Source: PLoS One. 2026 Jul 13;21(7):e0352260. doi: 10.1371/journal.pone.0352260 (PMC13362101; doi:10.1371/journal.pone.0352260)
Supplement: Attachment — Submitted filename: Response to Reviewers_R3.docx [file pone.0352260.s006.docx]

Dear Dr. Saumya Srivastava, Academic Editor, PLOS ONE,

Thank you for the opportunity to submit a further revised version of our manuscript entitled “Clinical efficacy of joint mobilization for shoulder impingement syndrome: a systematic review and meta-analysis” (Manuscript ID: PONE-D-24-07775R3).

We sincerely appreciate your positive evaluation of our manuscript and the helpful comments provided by you and the reviewers. We have carefully revised the manuscript to address all remaining points. Below, we provide a point-by-point response.

Academic Editor

Comment 1. Please briefly comment on how the high heterogeneity affects clinical applicability of the findings.

Response:

Thank you for this important comment. We have revised the Discussion section to more explicitly explain how the substantial heterogeneity affects clinical applicability. We clarified that the high I² values observed in several analyses indicate considerable variability in treatment effects, suggesting that the benefits of mobilization may not be consistent across all clinical contexts. We also noted that this variability likely reflects differences in mobilization techniques, treatment dosage, co-interventions, control conditions, and patient characteristics. Therefore, we emphasized that the findings should not be considered universally generalizable to all patients with shoulder impingement syndrome and that clinical application should be individualized.

Revised text:

“No adverse events were reported in the included studies. However, the results of this meta-analysis should be interpreted with caution due to substantial statistical heterogeneity, largely attributable to clinical diversity among studies. High I² values (often exceeding 90%) indicate considerable variability in treatment effects, suggesting that the benefits of mobilization are not consistent across all clinical contexts. This variability likely arises from differences in mobilization techniques, treatment dosage, co-interventions, control conditions, and patient characteristics. Consequently, the findings are not universally generalizable to all patients with shoulder impingement syndrome, and clinical application should be individualized. The heterogeneity also limits the precision of pooled estimates, reflecting the context-dependent nature of mobilization in practice.”

Comment 2. Consider adding a short paragraph outlining practical clinical implications.

Response:

Thank you for this helpful suggestion. We added a new paragraph under the “Clinical implications” section. In this paragraph, we clarified that joint mobilization may be considered as an adjunctive intervention, particularly for patients with limited range of motion, pain-related movement restriction, or difficulty engaging in active exercise-based rehabilitation. We also emphasized that treatment should be individualized according to clinical presentation and treatment context.

Revised text:

“From a clinical perspective, joint mobilization can be considered an adjunctive intervention for patients with shoulder impingement syndrome (SIS), particularly for those presenting with limited range of motion, pain-related movement restriction, or difficulty engaging in active exercise-based rehabilitation. It may facilitate early pain reduction and improve joint mobility, thereby promoting participation in subsequent active therapy. However, given the heterogeneity in intervention protocols and patient characteristics across studies, its application should be individualized according to the patient’s clinical presentation and treatment context.”

Comment 3. Where possible, briefly comment on whether observed effects reach clinically meaningful thresholds, such as MCID.

Response:

We appreciate this important comment. We added a brief discussion of clinical meaningfulness in addition to statistical significance. Specifically, we noted that the magnitude of pain reduction observed in this review may approach commonly cited MCID thresholds for shoulder pain. However, we interpreted this cautiously because outcome scales and study contexts varied across trials, and high heterogeneity limits definitive conclusions regarding clinical relevance.

Revised text:

“Beyond statistical significance, the clinical meaningfulness of the observed effects should be considered. For example, the magnitude of pain reduction observed in this study may approach commonly cited minimal clinically important difference (MCID) thresholds for shoulder pain. However, interpretation should remain cautious, as outcome measures (e.g., VAS 0–10 vs. 0–100) and study contexts varied across trials. Furthermore, the high heterogeneity limits the ability to draw definitive conclusions regarding the clinical relevance of these pooled estimates.”

Comment 4. Further streamline the Results section by minimizing repetition of individual study findings and emphasizing pooled outcomes.

Response:

Thank you for this constructive comment. We carefully revised the Results section to reduce repeated descriptions of individual trial findings and to place greater emphasis on pooled outcomes. In the previous version, several subsections included repeated study-level descriptions before or alongside the pooled estimates. In the revised manuscript, we retained essential information on the included comparisons while condensing repetitive narratives and presenting the main findings in a more concise, synthesis-oriented manner.

Specifically, we made the following changes:

1. In the comparison between mobilization plus physical therapy and physical therapy alone, we condensed the repeated study-level descriptions into an overall summary of the direction of findings. We then presented the pooled VAS, AROM, DASH, and Constant-Murley results more directly.

2. For VAS subgroup analyses, we combined repeated descriptions of VAS at rest, during activity, and across all days into a single concise paragraph while retaining the key MD, 95% CI, p value, and I² values.

3. For AROM subgroup analyses, we integrated the movement-specific results into one paragraph, reporting which movements showed significant improvement and which did not, while preserving the relevant pooled estimates and heterogeneity values.

4. In the comparison between mobilization plus physical therapy and exercise plus physical therapy, we reduced repeated individual-study narrative and summarized the overall pattern of findings before presenting the pooled VAS and ROM outcomes.

5. In the comparison between mobilization and sham mobilization, we retained the essential study-level context but condensed repeated subgroup descriptions. We also preserved the key pooled effects for VAS and AROM and retained the explanation for the “not estimable” study.

6. In the remaining comparisons, including comparisons between different mobilization approaches, mobilization versus other treatments, timing of intervention, and mobilization after SACS injection, we shortened repetitive wording while maintaining the original meaning and key findings.

Revised examples:

“A meta-analysis of eight studies using VAS immediately after treatment showed a statistically significant reduction in pain in the add-on group compared with the PT group (MD=1.72 [95% CI 0.91, 2.53], p<0.00001, I²=97%). Given the high heterogeneity, these results should be interpreted with caution. Subgroup analyses of VAS at different time points showed no statistically significant difference at rest (MD=0.58 [95% CI -0.16, 1.32], p=0.12, I²=78%), whereas significant improvements were observed during activity (MD=1.67 [95% CI 0.21, 3.14], p=0.03, I²=98%) and across all days (MD=3.00 [95% CI 2.78, 3.22], p<0.00001, I²=0%).”

“A meta-analysis of four studies using AROM immediately after treatment demonstrated a statistically significant improvement in the add-on group (MD=14.49 [95% CI 8.46, 20.53], p<0.00001, I²=95%). Subgroup analyses by movement direction showed significant improvements in flexion, internal rotation, and abduction, whereas no statistically significant differences were observed in extension and external rotation. Most subgroup analyses showed substantial heterogeneity, warranting cautious interpretation.”

“A meta-analysis of two studies using the VAS immediately after treatment demonstrated a statistically significant reduction in pain in the mobilization group (MD=0.67 [95% CI 0.15, 1.19], p=0.01, I²=0%). Subgroup analyses by time point showed no statistically significant differences at rest or during activity, with generally low heterogeneity. One study was labeled as ‘not estimable’ in part of the analysis due to insufficient variance information.”

We believe these revisions improve readability while preserving the essential quantitative findings and interpretive cautions.

Comment 5. Please review the manuscript for minor grammatical issues.

Response:

We have carefully reviewed the manuscript and corrected minor grammatical and typographical issues throughout the text.

Reviewer #5

Comment 1. Suggestion Title: Consider whether “joint mobilization” accurately reflects the umbrella definition you later use, including neuromobilization and soft tissue mobilization. The title may slightly underrepresent intervention heterogeneity.

Response:

Thank you for this thoughtful suggestion. We carefully considered whether the title should be changed. Because the term “joint mobilization” is widely used in the clinical and rehabilitation literature and is consistent with the main focus of most included studies, we retained the current title to preserve clarity and continuity. However, to address the concern that the term may underrepresent intervention heterogeneity, we clarified in the Introduction and Methods that mobilization was treated as a broader clinical category that included joint mobilization, neuromobilization, soft tissue mobilization, mobilization with movement, and dynamic humeral centering.

Comment 2. Minor suggestion in Abstract: Replace “clinical evidence” with “clinical effectiveness” for consistency.

Response:

Thank you for this suggestion. We revised the Abstract Background accordingly.

Revised text:

“We aimed to evaluate clinical effectiveness for the use of mobilization in SIS.”

Comment 3. Important issue in Abstract: You report mean differences (MD) but do not indicate scale units. Clinical interpretability is limited. No heterogeneity (I²) values were reported in the abstract despite very high I² in the results.

Response:

Thank you for pointing this out. To improve interpretability while maintaining the conciseness required for the Abstract, we added I² values for the two primary outcomes with very high heterogeneity: pain and active range of motion. We retained the key effect estimates and confidence intervals and avoided overloading the Abstract with too many heterogeneity values.

Revised text:

“Adding mobilization to traditional physical therapy reduced pain (mean difference [MD]=1.72, 95% confidence interval [CI]: 0.91 to 2.53, I²=97%, p<0.00001), increased active range of motion (MD=14.49, 95% CI: 8.46 to 20.53, I²=95%, p<0.00001)…”

Comment 4. Suggestion in Introduction: Add 1–2 sentences explaining why pooling heterogeneous mobilization types is clinically meaningful.

Response:

Thank you for this helpful suggestion. We added two sentences at the end of the Introduction to explain why evaluating heterogeneous mobilization techniques as a broader clinical category is clinically meaningful.

Revised text:

“Although mobilization techniques differ in their specific biomechanical and neurophysiological mechanisms, they share a common therapeutic goal of improving pain and movement dysfunction. Therefore, evaluating mobilization as a broader clinical category may provide useful insight into its overall role as an adjunctive treatment in patients with shoulder impingement syndrome.”

Comment 5. Minor suggestion in Methods: When grouping TENS, ultrasound, and heat under “physical therapy,” add one citation supporting this classification as standard conservative care.

Response:

Thank you for this suggestion. We carefully reviewed this point. As this was a minor suggestion rather than a required editorial change, and because the grouping was used as an operational classification for the meta-analysis, we retained the current wording without adding a new citation. We clarified in the Methods section that TENS, ultrasound, and heat therapy were grouped under “physical therapy” because they share common physiological goals of standard conservative care and are routinely administered together in clinical practice.

Relevant text:

“Various modalities such as TENS, ultrasound, and heat therapy were grouped under a single ‘physical therapy’ category for analysis, as they share the common physiological goals of standard conservative care (e.g., pain relief, tissue healing) and are routinely administered together in clinical settings.”

Comment 6. Results General comments: Major concern: Very high heterogeneity (I² > 90%). Consider adding exploration of heterogeneity, subgroup by type of mobilization, dosage, acute vs chronic, or meta-regression if feasible.

Response:

We agree that very high heterogeneity is an important methodological issue in this review. In the revised manuscript, we further clarified the clinical implications of high heterogeneity and emphasized that the pooled estimates should be interpreted cautiously. We explained that the variability likely arises from differences in mobilization techniques, dosage, co-interventions, control conditions, and patient characteristics.

We also considered additional subgroup analyses and meta-regression. However, these analyses were not statistically feasible because the number of studies in each specific comparison or technique category was small, and many interventions were combined with other physical therapy components. Therefore, we addressed this issue in the Discussion and Limitations sections rather than performing potentially underpowered analyses.

Revised text:

“High I² values (often exceeding 90%) indicate considerable variability in treatment effects, suggesting that the benefits of mobilization are not consistent across all clinical contexts. This variability likely arises from differences in mobilization techniques, treatment dosage, co-interventions, control conditions, and patient characteristics. Consequently, the findings are not universally generalizable to all patients with shoulder impingement syndrome, and clinical application should be individualized.”

Comment 7. Discussion: Discuss clinical significance, not just statistical significance. Is MD=1.72 on VAS clinically meaningful? MCID discussion recommended.

Response:

Thank you for this important recommendation. We added a paragraph discussing clinical meaningfulness in addition to statistical significance. We noted that the observed magnitude of pain reduction may approach commonly cited MCID thresholds for shoulder pain, but we interpreted this cautiously because outcome measures and study contexts varied across trials and heterogeneity was high.

Revised text:

“Beyond statistical significance, the clinical meaningfulness of the observed effects should be considered. For example, the magnitude of pain reduction observed in this study may approach commonly cited minimal clinically important difference (MCID) thresholds for shoulder pain. However, interpretation should remain cautious, as outcome measures (e.g., VAS 0–10 vs. 0–100) and study contexts varied across trials. Furthermore, the high heterogeneity limits the ability to draw definitive conclusions regarding the clinical relevance of these pooled estimates.”

Comment 8. Discussion: Clarify duration of effect.

Response:

Thank you for this comment. We maintained and reinforced the interpretation that the observed benefits should be understood primarily as short-term and adjunctive. This point is stated in both the Abstract Conclusion and the main Conclusion.

Relevant text:

“Mobilization may offer adjunctive, short-term benefits in improving the clinical symptoms of SIS when added to conventional treatments.”

“These benefits appear to be primarily short-term and adjunctive in nature.”

Comment 9. Discussion: Explicitly state that evidence certainty is low. If GRADE was not performed, acknowledge that certainty grading was not conducted.

Response:

Thank you for this important point. We added a statement in the Limitations section acknowledging that a formal GRADE assessment was not performed and that the certainty of evidence was therefore not formally evaluated.

Revised text:

“In addition, we did not perform a formal GRADE assessment; therefore, the certainty of the evidence was not formally evaluated. This further limits the strength of the conclusions drawn from this study.”

We believe that these revisions address all remaining concerns and further strengthen the manuscript’s clarity, clinical applicability, and balance of interpretation.

Sincerely,

Wonbae Ha

On behalf of all authors
