# Supplementary material for: Clinical efficacy of joint mobilization for shoulder impingement syndrome: a systematic review and meta-analysis
Source: PLoS One. 2026 Jul 13;21(7):e0352260. doi: 10.1371/journal.pone.0352260 (PMC13362101; doi:10.1371/journal.pone.0352260)
Supplement: Peer review history

# Peer review: Decision Letter 0

**DOI:** 10.1371/journal.pone.0352260.r001

**Authors:** Sahreen Anwar

2 Oct 2024

PONE-D-24-07775

Clinical efficacy of joint mobilization for shoulder impingement syndrome: a systematic review and meta-analysis

PLOS ONE

Dear Dr. Ha,

Thank you for submitting your manuscript to PLOS ONE. After careful consideration, we feel that it has merit but does not fully meet PLOS ONE’s publication criteria as it currently stands. Therefore, we invite you to submit a revised version of the manuscript that addresses the points raised during the review process.

==============================

**ACADEMIC EDITOR:**   Please provide more detailed information regarding the inclusion and exclusion criteria used for selecting studies in your systematic review. This will enhance the transparency of your methodology.

Please ensure that the rationale for your chosen statistical tests is clearly stated. While you have identified several limitations, expanding this section to include potential biases in the included studies would strengthen your manuscript. Please review the formatting of your references to ensure consistency with the journal’s guidelines. A few references need to be double-checked for completeness.

I appreciate the effort you have put into this important research, and I believe that addressing these comments will significantly enhance the quality of your manuscript. I look forward to receiving your revised submission.

-->==============================

Please submit your revised manuscript by Nov 15 2024 11:59PM. If you will need more time than this to complete your revisions, please reply to this message or contact the journal office at plosone@plos.org. When you’re ready to submit your revision, log on to https://www.editorialmanager.com/pone/ and select the 'Submissions Needing Revision' folder to locate your manuscript file.

Please include the following items when submitting your revised manuscript:

- A rebuttal letter that responds to each point raised by the academic editor and reviewer(s). You should upload this letter as a separate file labeled 'Response to Reviewers'.
- A marked-up copy of your manuscript that highlights changes made to the original version. You should upload this as a separate file labeled 'Revised Manuscript with Track Changes'.
- An unmarked version of your revised paper without tracked changes. You should upload this as a separate file labeled 'Manuscript'.

If you would like to make changes to your financial disclosure, please include your updated statement in your cover letter. Guidelines for resubmitting your figure files are available below the reviewer comments at the end of this letter.

If applicable, we recommend that you deposit your laboratory protocols in protocols.io to enhance the reproducibility of your results. Protocols.io assigns your protocol its own identifier (DOI) so that it can be cited independently in the future. For instructions see: https://journals.plos.org/plosone/s/submission-guidelines#loc-laboratory-protocols. Additionally, PLOS ONE offers an option for publishing peer-reviewed Lab Protocol articles, which describe protocols hosted on protocols.io. Read more information on sharing protocols at https://plos.org/protocols?utm_medium=editorial-email&utm_source=authorletters&utm_campaign=protocols.

We look forward to receiving your revised manuscript.

Kind regards,

Sahreen Anwar, PhD Physical Therapy

Academic Editor

PLOS ONE

Journal Requirements:

When submitting your revision, we need you to address these additional requirements.

1. Please ensure that your manuscript meets PLOS ONE's style requirements, including those for file naming. The PLOS ONE style templates can be found at

https://journals.plos.org/plosone/s/file?id=wjVg/PLOSOne_formatting_sample_main_body.pdf and

https://journals.plos.org/plosone/s/file?id=ba62/PLOSOne_formatting_sample_title_authors_affiliations.pdf

2. We note that the grant information you provided in the ‘Funding Information’ and ‘Financial Disclosure’ sections do not match.

When you resubmit, please ensure that you provide the correct grant numbers for the awards you received for your study in the ‘Funding Information’ section.

3. Please review your reference list to ensure that it is complete and correct. If you have cited papers that have been retracted, please include the rationale for doing so in the manuscript text, or remove these references and replace them with relevant current references. Any changes to the reference list should be mentioned in the rebuttal letter that accompanies your revised manuscript. If you need to cite a retracted article, indicate the article’s retracted status in the References list and also include a citation and full reference for the retraction notice.

4. As required by our policy on Data Availability, please ensure your manuscript or supplementary information includes the following:

A numbered table of all studies identified in the literature search, including those that were excluded from the analyses.

For every excluded study, the table should list the reason(s) for exclusion.

If any of the included studies are unpublished, include a link (URL) to the primary source or detailed information about how the content can be accessed.

A table of all data extracted from the primary research sources for the systematic review and/or meta-analysis. The table must include the following information for each study:

Name of data extractors and date of data extraction

Confirmation that the study was eligible to be included in the review.

All data extracted from each study for the reported systematic review and/or meta-analysis that would be needed to replicate your analyses.

If data or supporting information were obtained from another source (e.g. correspondence with the author of the original research article), please provide the source of data and dates on which the data/information were obtained by your research group.

If applicable for your analysis, a table showing the completed risk of bias and quality/certainty assessments for each study or outcome.  Please ensure this is provided for each domain or parameter assessed. For example, if you used the Cochrane risk-of-bias tool for randomized trials, provide answers to each of the signalling questions for each study. If you used GRADE to assess certainty of evidence, provide judgements about each of the quality of evidence factor. This should be provided for each outcome.

An explanation of how missing data were handled.

This information can be included in the main text, supplementary information, or relevant data repository. Please note that providing these underlying data is a requirement for publication in this journal, and if these data are not provided your manuscript might be rejected.

**Additional Editor Comments:**

Comments from the staff editor team (plosone@plos.org): Please discuss the implications of the high level of heterogeneity between studies, and the high risk of bias.

Reviewers' comments:

Reviewer's Responses to Questions

**Comments to the Author**

1. Is the manuscript technically sound, and do the data support the conclusions?

The manuscript must describe a technically sound piece of scientific research with data that supports the conclusions. Experiments must have been conducted rigorously, with appropriate controls, replication, and sample sizes. The conclusions must be drawn appropriately based on the data presented.

Reviewer #1: Yes

**********

2. Has the statistical analysis been performed appropriately and rigorously?

Reviewer #1: Yes

**********

3. Have the authors made all data underlying the findings in their manuscript fully available?

The PLOS Data policy requires authors to make all data underlying the findings described in their manuscript fully available without restriction, with rare exception (please refer to the Data Availability Statement in the manuscript PDF file). The data should be provided as part of the manuscript or its supporting information, or deposited to a public repository. For example, in addition to summary statistics, the data points behind means, medians and variance measures should be available. If there are restrictions on publicly sharing data—e.g. participant privacy or use of data from a third party—those must be specified.

Reviewer #1: Yes

**********

4. Is the manuscript presented in an intelligible fashion and written in standard English?

PLOS ONE does not copyedit accepted manuscripts, so the language in submitted articles must be clear, correct, and unambiguous. Any typographical or grammatical errors should be corrected at revision, so please note any specific errors here.

Reviewer #1: Yes

**********

5. Review Comments to the Author

Please use the space provided to explain your answers to the questions above. You may also include additional comments for the author, including concerns about dual publication, research ethics, or publication ethics. (Please upload your review as an attachment if it exceeds 20,000 characters)

Reviewer #1: shoulder impingement is a common condition encountered by many clinicians in their clinical practice, this systematic review with meta analysis gives a summarized information from various parts of the world on the subject title.

**********

6. PLOS authors have the option to publish the peer review history of their article (what does this mean?). If published, this will include your full peer review and any attached files.

If you choose “no”, your identity will remain anonymous but your review may still be made public.

**Do you want your identity to be public for this peer review?**  For information about this choice, including consent withdrawal, please see our Privacy Policy.

Reviewer #1: **Yes:**  Wajida Perveen

**********

[NOTE: If reviewer comments were submitted as an attachment file, they will be attached to this email and accessible via the submission site. Please log into your account, locate the manuscript record, and check for the action link "View Attachments". If this link does not appear, there are no attachment files.]

While revising your submission, please upload your figure files to the Preflight Analysis and Conversion Engine (PACE) digital diagnostic tool, https://pacev2.apexcovantage.com/. PACE helps ensure that figures meet PLOS requirements. To use PACE, you must first register as a user. Registration is free. Then, login and navigate to the UPLOAD tab, where you will find detailed instructions on how to use the tool. If you encounter any issues or have any questions when using PACE, please email PLOS at figures@plos.org. Please note that Supporting Information files do not need this step.

# Author comment: Author response to Decision Letter 1

**DOI:** 10.1371/journal.pone.0352260.r002

11 Nov 2024

We have revised the manuscript and supplement information files based on the comments recieved. Details of the revisions are explained in the attached file(Response to Reviewers) for your review. Thank you sincerely for your valuable feedback.

Attachment Submitted filename: Response to Reviewers.docx

# Peer review: Decision Letter 1

**DOI:** 10.1371/journal.pone.0352260.r003

**Authors:** Sahreen Anwar, Saumya Srivastava

29 Jul 2025

PONE-D-24-07775R1

Clinical efficacy of joint mobilization for shoulder impingement syndrome: a systematic review and meta-analysis

PLOS ONE

Dear Dr. Ha,

Thank you for submitting your manuscript to PLOS ONE. After careful consideration, we feel that it has merit but does not fully meet PLOS ONE’s publication criteria as it currently stands. Therefore, we invite you to submit a revised version of the manuscript that addresses the points raised during the review process.

==============================

The key areas that require your attention are as follows:

**Statistical Heterogeneity** : Many meta-analyses reported high I² values, which compromise interpretability. You are advised to explore sources of heterogeneity through subgroup or sensitivity analyses and discuss their impact in the manuscript.

**Definition and Categorization of Interventions** : The term “mobilization” is used to refer to a broad range of techniques. Clear operational definitions and subgroup analyses based on technique type are recommended.

**Risk of Bias and Methodological Quality** : A significant proportion of the included studies are at high risk of bias, particularly in blinding and outcome assessment. This should be discussed more explicitly as a limitation.

**Limitations and Future Directions** : The discussion section should better acknowledge the methodological limitations of the included studies and propose directions for future high-quality research.

==============================

Please submit your revised manuscript by Sep 12 2025 11:59PM. If you will need more time than this to complete your revisions, please reply to this message or contact the journal office at plosone@plos.org. When you're ready to submit your revision, log on to https://www.editorialmanager.com/pone/ and select the 'Submissions Needing Revision' folder to locate your manuscript file.

Please include the following items when submitting your revised manuscript:

- A rebuttal letter that responds to each point raised by the academic editor and reviewer(s). You should upload this letter as a separate file labeled 'Response to Reviewers'.
- A marked-up copy of your manuscript that highlights changes made to the original version. You should upload this as a separate file labeled 'Revised Manuscript with Track Changes'.
- An unmarked version of your revised paper without tracked changes. You should upload this as a separate file labeled 'Manuscript'.

If you would like to make changes to your financial disclosure, please include your updated statement in your cover letter. Guidelines for resubmitting your figure files are available below the reviewer comments at the end of this letter.

If applicable, we recommend that you deposit your laboratory protocols in protocols.io to enhance the reproducibility of your results. Protocols.io assigns your protocol its own identifier (DOI) so that it can be cited independently in the future. For instructions see: https://journals.plos.org/plosone/s/submission-guidelines#loc-laboratory-protocols. Additionally, PLOS ONE offers an option for publishing peer-reviewed Lab Protocol articles, which describe protocols hosted on protocols.io. Read more information on sharing protocols at https://plos.org/protocols?utm_medium=editorial-email&utm_source=authorletters&utm_campaign=protocols.

We look forward to receiving your revised manuscript.

Kind regards,

Saumya Srivastava, Ph.D.

Academic Editor

PLOS ONE

Journal Requirements:

If the reviewer comments include a recommendation to cite specific previously published works, please review and evaluate these publications to determine whether they are relevant and should be cited. There is no requirement to cite these works unless the editor has indicated otherwise.

**Additional Editor Comments:**

Dear Dr. Won-Bae Ha and Co-authors,

Thank you for submitting your manuscript titled "Clinical efficacy of joint mobilization for shoulder impingement syndrome: a systematic review and meta-analysis" (Manuscript ID: PONE-D-24-07775R1) to PLOS ONE. The manuscript addresses a clinically relevant and timely topic, and we appreciate the comprehensive approach taken to explore the effects of joint mobilization for shoulder impingement syndrome. However, after careful evaluation, we believe that major revisions are required before the manuscript can be considered further for publication. Please find the reviewers’ reports attached/at the end of this email.

We encourage you to respond to each comment systematically in a point-by-point response letter and revise your manuscript accordingly.

Best regards,

Dr Saumya Srivastava,

Academic Editor, PLOS ONE

[Note: HTML markup is below. Please do not edit.]

Reviewers' comments:

Reviewer's Responses to Questions

**Comments to the Author**

1. If the authors have adequately addressed your comments raised in a previous round of review and you feel that this manuscript is now acceptable for publication, you may indicate that here to bypass the “Comments to the Author” section, enter your conflict of interest statement in the “Confidential to Editor” section, and submit your "Accept" recommendation.

Reviewer #1: All comments have been addressed

Reviewer #2: All comments have been addressed

Reviewer #3: (No Response)

**********

2. Is the manuscript technically sound, and do the data support the conclusions?

The manuscript must describe a technically sound piece of scientific research with data that supports the conclusions. Experiments must have been conducted rigorously, with appropriate controls, replication, and sample sizes. The conclusions must be drawn appropriately based on the data presented.

Reviewer #1: Yes

Reviewer #2: Yes

Reviewer #3: Partly

**********

3. Has the statistical analysis been performed appropriately and rigorously?

Reviewer #1: Yes

Reviewer #2: Yes

Reviewer #3: No

**********

4. Have the authors made all data underlying the findings in their manuscript fully available?

The PLOS Data policy requires authors to make all data underlying the findings described in their manuscript fully available without restriction, with rare exception (please refer to the Data Availability Statement in the manuscript PDF file). The data should be provided as part of the manuscript or its supporting information, or deposited to a public repository. For example, in addition to summary statistics, the data points behind means, medians and variance measures should be available. If there are restrictions on publicly sharing data—e.g. participant privacy or use of data from a third party—those must be specified.

Reviewer #1: Yes

Reviewer #2: Yes

Reviewer #3: Yes

**********

5. Is the manuscript presented in an intelligible fashion and written in standard English?

PLOS ONE does not copyedit accepted manuscripts, so the language in submitted articles must be clear, correct, and unambiguous. Any typographical or grammatical errors should be corrected at revision, so please note any specific errors here.

Reviewer #1: Yes

Reviewer #2: Yes

Reviewer #3: Yes

**********

6. Review Comments to the Author

Please use the space provided to explain your answers to the questions above. You may also include additional comments for the author, including concerns about dual publication, research ethics, or publication ethics. (Please upload your review as an attachment if it exceeds 20,000 characters)

Reviewer #1: the keywords are missing in abstract.

I believe that the Language is appropriate and manuscript may be proceeded for publication.

Reviewer #2: Suggest to also add statements on exclusion and exclusion with relation to year of publication and language . Have you accepted all primary and secondary SAS, patients with previous surgeries, previous fractures etc..

Reviewer #3: Dear authors,

Congratulations on this strong research idea. The article is well structured and accompanied by relevant information. However, there are a few aspects that should be improved.

Abstract- keywords

a. Include important information about the method that was used in this systematic review and meta-analyses – PRISMA.

b. In the conclusion of the abstract, the study's limitations are highlighted: “Owing to the clinical heterogeneity, uncertain risk of bias, and small number of studies, high-quality studies evaluating the effectiveness of mobilization are warranted”.

Given the significant limitations mentioned, I suggest reformulating the approach to provide a clearer direction for future research.

Introduction

a. Since the sham mobilization was used as a comparison in row 34, I suggest inserting information about this in the Introduction section. It is a new concept, and adding information in the Introduction section will help emphasize its relevance.

Methods

a. Please provide in this section the link for Data Availability.

b. Please provide a reference for the Cochrane Handbook for Systematic Reviews of Interventions. And also for the PRISMA guideline (rows 80-81).

Database selection and search

a. For selecting relevant keywords, it was preferred to use an automated tool, such as MeSH or the Word Frequency Analyzer Tool.

b. Please explain why the searching strategy was used: “mobilization” and “mobilization” – row 91?

Inclusion and exclusion criteria

a. Please improve this section to add clarity and flow.

b. It is essential to include the abbreviations for participants, interventions, comparisons, outcomes, and study designs that refer to PICOS- rows 95-96.

b. Additionally, since PICOS was used, the exclusion criteria need to follow the structure to ensure methodological consistency.

Literature selection and analysis

a. Please remove the duplicate of the subtitle – rows 106- 107

b. Please mention where the selected articles were exported by the two researchers (GL and WH)- 112-113. In the abstract, it is noted that EndNote 20 was used.

c. Before applying Rob 2.0 to identify the risk of bias, a tool was used to screen and select studies? This step is crucial to ensure that only eligible studies are included in the meta-analysis, especially through the peer-blinding process. E.g., Ryyan?

3. Discussion

a. In rows 370-371, it is mentioned that the meta-analysis was performed on 11 studies out of the 19. Please argue why the meta-analysis was made on 11 studies and why it isn’t mentioned in the Method section.

b. Regarding some indices, a high level of heterogeneity was noted in the Result section (eg, rows 192, 195, 200, 205, 211), which could affect the ability to draw firm conclusions about the effectiveness of joint mobilization (rows 372, 273).

c. Since the aim of the study was to examine the clinical effectiveness of mobilization for SIS, it is highly suggested to focus on this, not on clinical implications- this subsection could be just an introduction for the summary.

General recommendation:

a. While the topic is relevant and valuable for the physical therapy area, several methodological aspects require clarification; please see the aforementioned points.

b. Strongly recommend keeping “joint mobilization” and not “mobilization”, as this is the term mentioned in the title and abstract as well.

**********

7. PLOS authors have the option to publish the peer review history of their article (what does this mean?). If published, this will include your full peer review and any attached files.

If you choose “no”, your identity will remain anonymous but your review may still be made public.

**Do you want your identity to be public for this peer review?**  For information about this choice, including consent withdrawal, please see our Privacy Policy.

Reviewer #1: **Yes:**  Wajida Perveen

Reviewer #2: **Yes:**  Ahmed Ibrahim Al Kharusi

Reviewer #3: **Yes:**  Dan Iulian Alexe

**********

[NOTE: If reviewer comments were submitted as an attachment file, they will be attached to this email and accessible via the submission site. Please log into your account, locate the manuscript record, and check for the action link "View Attachments". If this link does not appear, there are no attachment files.]

While revising your submission, please upload your figure files to the Preflight Analysis and Conversion Engine (PACE) digital diagnostic tool, https://pacev2.apexcovantage.com/. PACE helps ensure that figures meet PLOS requirements. To use PACE, you must first register as a user. Registration is free. Then, login and navigate to the UPLOAD tab, where you will find detailed instructions on how to use the tool. If you encounter any issues or have any questions when using PACE, please email PLOS at figures@plos.org. Please note that Supporting Information files do not need this step.

# Author comment: Author response to Decision Letter 2

**DOI:** 10.1371/journal.pone.0352260.r004

21 Nov 2025

Thank you for reading and evaluating our manuscript. We have revised and are resubmitting the paper in accordance with the valuable suggestions from the editor and reviewers.

Detailed responses to all of the comments and a list of revisions have been provided in the separate "Response to Reviewers" document.

We hope that this revision has successfully improved the quality of our manuscript and addressed the concerns that were raised.

Attachment Submitted filename: Response_to_Reviewers_auresp_2.docx

# Peer review: Decision Letter 2

**DOI:** 10.1371/journal.pone.0352260.r005

**Authors:** Sahreen Anwar, Saumya Srivastava, Saumya Srivastava

9 Feb 2026

PONE-D-24-07775R2

Clinical efficacy of joint mobilization for shoulder impingement syndrome: a systematic review and meta-analysis

PLOS One

Dear Dr. Ha,

Thank you for submitting your manuscript to PLOS ONE. After careful consideration, we feel that it has merit but does not fully meet PLOS ONE’s publication criteria as it currently stands. Therefore, we invite you to submit a revised version of the manuscript that addresses the points raised during the review process.

==============================

**ACADEMIC EDITOR: Please insert comments here and delete this placeholder text when finished.**  Be sure to:

1. Clarify and emphasize the conceptual and clinical heterogeneity of the various “mobilization” techniques included.
2. More explicitly discuss the implications of high heterogeneity observed in several meta-analyses.
3. Temper the conclusions to reflect the adjunctive and primarily short-term nature of the observed effects.

-->Please ensure that your decision is justified on PLOS ONE’s publication criteria and not, for example, on novelty or perceived impact.

For Lab, Study and Registered Report Protocols: These article types are not expected to include results but may include pilot data.

==============================

Please submit your revised manuscript by Mar 26 2026 11:59PM. If you will need more time than this to complete your revisions, please reply to this message or contact the journal office at plosone@plos.org. When you're ready to submit your revision, log on to https://www.editorialmanager.com/pone/ and select the 'Submissions Needing Revision' folder to locate your manuscript file.

Please include the following items when submitting your revised manuscript:

- A letter that responds to each point raised by the academic editor and reviewer(s). You should upload this letter as a separate file labeled 'Response to Reviewers'.
- A marked-up copy of your manuscript that highlights changes made to the original version. You should upload this as a separate file labeled 'Revised Manuscript with Track Changes'.
- An unmarked version of your revised paper without tracked changes. You should upload this as a separate file labeled 'Manuscript'.

-->If you would like to make changes to your financial disclosure, please include your updated statement in your cover letter. Guidelines for resubmitting your figure files are available below the reviewer comments at the end of this letter.

If applicable, we recommend that you deposit your laboratory protocols in protocols.io to enhance the reproducibility of your results. Protocols.io assigns your protocol its own identifier (DOI) so that it can be cited independently in the future. For instructions see: https://journals.plos.org/plosone/s/submission-guidelines#loc-laboratory-protocols. Additionally, PLOS ONE offers an option for publishing peer-reviewed Lab Protocol articles, which describe protocols hosted on protocols.io. Read more information on sharing protocols at https://plos.org/protocols?utm_medium=editorial-email&utm_source=authorletters&utm_campaign=protocols.

We look forward to receiving your revised manuscript.

Kind regards,

Saumya Srivastava, Ph.D.

Academic Editor

PLOS One

Journal Requirements:

1. If the reviewer comments include a recommendation to cite specific previously published works, please review and evaluate these publications to determine whether they are relevant and should be cited. There is no requirement to cite these works unless the editor has indicated otherwise.

2. Please review your reference list to ensure that it is complete and correct. If you have cited papers that have been retracted, please include the rationale for doing so in the manuscript text, or remove these references and replace them with relevant current references. Any changes to the reference list should be mentioned in the rebuttal letter that accompanies your revised manuscript. If you need to cite a retracted article, indicate the article’s retracted status in the References list and also include a citation and full reference for the retraction notice.

Additional Editor Comments:

Dear Dr. Ha,

Thank you for submitting the revised version of your manuscript entitled “Clinical efficacy of joint mobilization for shoulder impingement syndrome: a systematic review and meta-analysis” (Manuscript ID: PONE-D-24-07775R2) to PLOS ONE.

I have now completed the assessment of the second revised manuscript. I would like to commend the authors for the substantial improvements made in response to the previous editorial and reviewer comments. The manuscript has improved notably in terms of methodological transparency, reporting clarity, and overall structure. The study addresses a clinically relevant question and is generally consistent with PLOS ONE’s publication criteria regarding technical soundness and reproducibility.

However, after careful consideration, I find that a small number of issues still require attention before the manuscript can be accepted for publication. These issues primarily relate to clarification of interpretation and appropriate framing of conclusions.

These revisions are considered minor but essential to ensure that the manuscript’s conclusions are appropriately balanced and accurately reflect the underlying evidence base.

Regards,

Dr Saumya Srivastava,

Academic Editor,

PLOS One

[Note: HTML markup is below. Please do not edit.]

Reviewers' comments:

Reviewer's Responses to Questions

**Comments to the Author**

1. If the authors have adequately addressed your comments raised in a previous round of review and you feel that this manuscript is now acceptable for publication, you may indicate that here to bypass the “Comments to the Author” section, enter your conflict of interest statement in the “Confidential to Editor” section, and submit your "Accept" recommendation.

Reviewer #3: All comments have been addressed

Reviewer #4: All comments have been addressed

Reviewer #5: (No Response)

**********

2. Is the manuscript technically sound, and do the data support the conclusions?

The manuscript must describe a technically sound piece of scientific research with data that supports the conclusions. Experiments must have been conducted rigorously, with appropriate controls, replication, and sample sizes. The conclusions must be drawn appropriately based on the data presented.

Reviewer #3: Yes

Reviewer #4: Yes

Reviewer #5: Yes

**********

3. Has the statistical analysis been performed appropriately and rigorously?

Reviewer #3: Yes

Reviewer #4: Yes

Reviewer #5: Yes

**********

4. Have the authors made all data underlying the findings in their manuscript fully available?

The PLOS Data policy requires authors to make all data underlying the findings described in their manuscript fully available without restriction, with rare exception (please refer to the Data Availability Statement in the manuscript PDF file). The data should be provided as part of the manuscript or its supporting information, or deposited to a public repository. For example, in addition to summary statistics, the data points behind means, medians and variance measures should be available. If there are restrictions on publicly sharing data—e.g. participant privacy or use of data from a third party—those must be specified.

Reviewer #3: Yes

Reviewer #4: Yes

Reviewer #5: Yes

**********

5. Is the manuscript presented in an intelligible fashion and written in standard English?

PLOS ONE does not copyedit accepted manuscripts, so the language in submitted articles must be clear, correct, and unambiguous. Any typographical or grammatical errors should be corrected at revision, so please note any specific errors here.

Reviewer #3: Yes

Reviewer #4: Yes

Reviewer #5: Yes

**********

6. Review Comments to the Author

Please use the space provided to explain your answers to the questions above. You may also include additional comments for the author, including concerns about dual publication, research ethics, or publication ethics. (Please upload your review as an attachment if it exceeds 20,000 characters)

Reviewer #3: The authors have responded to the comments and made the necessary adjustments.

I have no further comments to make.

Reviewer #4: The manuscript has been substantially improved in this revised version, only minor revisions are recommended:

Abstract:

In the Results section of the abstract, the term physiotherapy is used to refer to physical therapy. Please use consistent terminology throughout the abstract and manuscript.

Introduction:

Lines 70–74: This paragraph introduces the importance of sham mobilization before sham mobilization is clearly defined earlier in the manuscript. Please revise this paragraph for clarity by first defining sham mobilization in clear terms

Line 79: The statement that “no studies have reported the effectiveness of mobilization for SIS” is unclear. If the intention is to state that no prior systematic reviews have evaluated this question, please revise and re-write.

Methods:

Classification of Physical Therapy:

Please provide a brief rationale for grouping different physical therapy modalities under the single category of physical therapy in the analysis. You need to show that it can be combined!

Line 89: The inclusion of database URLs in parentheses is unnecessary and can be removed.

Line 93: The term “mobilization” appears to be repeated; please correct for clarity.

Line 109: The phrase “received interventions overlapping with mobilization that could not be separated” is unclear, particularly since studies including mobilization plus other interventions were allowed under the inclusion criteria. Please clarify what is meant by “overlapping” and how “could not be separated” was operationally defined.

Line 116: The phrase “or study design” may be misleading, as only randomized controlled trials were included. Please revise to reflect that study design was, in fact, restricted to RCTs.

Results:

The Results section provides detailed descriptions of individual trials prior to presenting pooled estimates. Consider condensing trial-level narrative and placing greater emphasis on meta-analytic results, as study-level details are already summarized in tables.

Figures:

Figure 8: One study is labeled as “not estimable.” Please explicitly state the reason (e.g., insufficient variance information or inability to calculate a standard error for the inverse-variance model) in the Results.

Reviewer #5: Abstract: Temper conclusion with cautionary language

Methods: Justify model choice (please justify your choice of the random-effects model (e.g., “due to anticipated clinical and methodological heterogeneity”)), add publication bias assessment (describe whether funnel plots or Egger’s tests were planned/performed, especially for meta-analyses with ≥10 studies. If not performed, please explain why) and clarify missing data handling (the statement “we used a method that did not include missing data” is vague). Please specify the approach (e.g., complete-case analysis) and whether sensitivity analyses were conducted to assess robustness).

Results: Comment on high heterogeneity (when reporting I² values >90%, please add a brief comment on the implications for interpretability (e.g., “Results should be interpreted with caution due to substantial heterogeneity.”), and add sensitivity analysis for high RoB studies.

Discussion: Expand limitations (publication bias, multiple testing, subgroup caution)

Editorial: Remove duplicate abstract, complete tables, and clarify search date

**********

7. PLOS authors have the option to publish the peer review history of their article (what does this mean?). If published, this will include your full peer review and any attached files.

If you choose “no”, your identity will remain anonymous but your review may still be made public.

**Do you want your identity to be public for this peer review?**  For information about this choice, including consent withdrawal, please see our Privacy Policy.

Reviewer #3: **Yes:**  Dan Iulian Alexe

Reviewer #4: No

Reviewer #5: **Yes:**  Shumi Negawo, Madda Walabu University, Ethiopia

**********

[NOTE: If reviewer comments were submitted as an attachment file, they will be attached to this email and accessible via the submission site. Please log into your account, locate the manuscript record, and check for the action link "View Attachments". If this link does not appear, there are no attachment files.]

To ensure your figures meet our technical requirements, please review our figure guidelines: https://journals.plos.org/plosone/s/figures

You may also use PLOS’s free figure tool, NAAS, to help you prepare publication quality figures: https://journals.plos.org/plosone/s/figures#loc-tools-for-figure-preparation.

NAAS will assess whether your figures meet our technical requirements by comparing each figure against our figure specifications.

# Author comment: Author response to Decision Letter 3

**DOI:** 10.1371/journal.pone.0352260.r006

15 Feb 2026

Response to Reviewers: (PONE-D-24-07775R2, EMID:874ef5f01d7721ab)

Dear Dr. Saumya Srivastava, Academic Editor, PLOS ONE

Thank you for the opportunity to submit a revised version of our manuscript, "Clinical efficacy of joint mobilization for shoulder impingement syndrome: a systematic review and meta-analysis" (PONE-D-24-07775R2). We are grateful to you and the reviewers for the constructive feedback, which has further improved the quality and clarity of our manuscript.

[Academic editor]

Point 1: Clarify and emphasize the conceptual and clinical heterogeneity of the various “mobilization” techniques included.

Response: Thank you for this valuable feedback. We have revised the "Operational Definitions of Interventions" in the Methods section to more explicitly emphasize the conceptual and clinical heterogeneity of the various mobilization techniques included in our study (e.g., joint mobilization, neuromobilization, soft tissue mobilization, and MWM).

[Method] - [Operational Definitions of Interventions]

… More specific methods included Mobilization with Movement (MWM), a technique combining a sustained accessory joint glide by the therapist with the patient's active physiological movement, and Dynamic Humeral Centering (DHC), a manual technique aimed at correcting the position of the humeral head within the glenoid fossa during movement to reduce subacromial impingement.

→ … More specific methods included Mobilization with Movement (MWM), a technique combining a sustained accessory joint glide by the therapist with the patient's active physiological movement, and Dynamic Humeral Centering (DHC), a manual technique aimed at correcting the position of the humeral head within the glenoid fossa during movement to reduce subacromial impingement. These techniques, while sharing the overarching goal of restoring mobility, operate through distinct biomechanical and neurophysiological mechanisms, contributing to conceptual heterogeneity.

Point 2: More explicitly discuss the implications of high heterogeneity observed in several meta-analyses.

Response: We agree with your assessment. In the Discussion section, we have explicitly expanded upon the implications of the high heterogeneity (e.g., I² > 90%) observed in several of our meta-analyses. We added language cautioning readers that these pooled estimates should be interpreted carefully, as the high statistical heterogeneity is likely a direct result of the diverse clinical protocols, techniques, and co-interventions used across the included trials.

[Discussion] – [Summary of findings]

… The results of this meta-analysis should be interpreted with caution due to significant statistical heterogeneity, which primarily stems from the considerable clinical diversity among the included studies. This diversity was evident in several areas …

→ … The results of this meta-analysis should be interpreted with caution due to significant statistical heterogeneity, which primarily stems from the considerable clinical diversity among the included studies. Specifically, the high I² values (frequently >90%) observed in several meta-analyses compromise the precision of the pooled estimates. This indicates that the observed effects are highly variable and context-dependent, directly reflecting the diverse nature of 'mobilization' in clinical practice. This diversity was evident in several areas …

Point 3: Temper the conclusions to reflect the adjunctive and primarily short-term nature of the observed effects.

Response: We have appropriately tempered the conclusions in both the Abstract and the main Conclusion sections. We have revised the text to clearly state that joint mobilization should be considered an adjunctive treatment to conventional physical therapy, and we emphasized that the observed benefits are primarily short-term, reflecting the limitations of the current evidence base.

[Abstract]

Conclusion: Mobilization may be effective in improving the clinical symptoms of SIS and may be recommended in addition to conventional treatments

→ Conclusion: Mobilization may offer adjunctive, short-term benefits in improving the clinical symptoms of SIS when added to conventional treatments.

[Conclusion]

Therefore, clinically, mobilization may be more advisable when patients have weak muscle strength or limited AROM or when an acute injury prevents active movement. However, the quality of the evidence was not high, owing to the high heterogeneity among the included studies and the inclusion of a large number of studies with a high risk of bias assessment.

→ Therefore, clinically, mobilization may be more advisable when patients have weak muscle strength or limited AROM or when an acute injury prevents active movement. These benefits appear to be primarily short-term and adjunctive in nature. However, the quality of the evidence was not high, owing to the high heterogeneity among the included studies and the inclusion of a large number of studies with a high risk of bias assessment.

[Reviewer #4]

Point 1: In the Results section of the abstract, the term physiotherapy is used to refer to physical therapy. Please use consistent terminology throughout the abstract and manuscript.

Response: We apologize for the inconsistency. We have changed "physiotherapy" to "physical therapy" in the Results section of the Abstract, ensuring uniform terminology throughout the entire manuscript.

[Abstract]

Results: Nineteen randomized controlled trials (956 participants) were selected based on the inclusion criteria. Adding mobilization to traditional physiotherapy reduced pain ...

→ Results: Nineteen randomized controlled trials (956 participants) were selected based on the inclusion criteria. Adding mobilization to traditional physical therapy reduced pain …

Point 2: (Lines 70–74) This paragraph introduces the importance of sham mobilization before sham mobilization is clearly defined earlier in the manuscript. Please revise this paragraph for clarity by first defining sham mobilization in clear terms

Response: Following your suggestion, we have inserted a clear operational definition of sham mobilization in the Introduction prior to discussing its importance, improving the clarity of this paragraph.

[Introduction]

These mobilizations can be applied to patients with SIS to improve shoulder joint pain and restore joint function. However, to determine …

→ These mobilizations can be applied to patients with SIS to improve shoulder joint pain and restore joint function. In research settings, sham mobilization is utilized as a placebo control, involving manual contact by the therapist without delivering the actual therapeutic force or specific joint movement. However, to determine …

Point 3: (Line 79) The statement that “no studies have reported the effectiveness of mobilization for SIS” is unclear. If the intention is to state that no prior systematic reviews have evaluated this question, please revise and re-write.

Response: Thank you for pointing this out. We agree that the final sentence was unclear and potentially misleading. Since we already explicitly stated earlier in the same paragraph that "no systematic review has examined the effects of mobilization as a whole on SIS," the final sentence was redundant. To avoid any confusion and improve the flow of the text, we have completely deleted the problematic sentence ("However, no studies have reported the effectiveness of mobilization for SIS.") from the revised manuscript.

[Introduction]

Furthermore, studies have reported the effectiveness of mobilization for various conditions, including ankle sprains [13], ankle joint instability [14], knee osteoarthritis [15], carpal tunnel syndrome [16], and frozen shoulder [17, 18]. However, no studies have reported the effectiveness of mobilization for SIS.

→ Furthermore, studies have reported the effectiveness of mobilization for various conditions, including ankle sprains [13], ankle joint instability [14], knee osteoarthritis [15], carpal tunnel syndrome [16], and frozen shoulder [17, 18]. However, no studies have reported the effectiveness of mobilization for SIS.

Point 4: (Line 79) Classification of Physical Therapy: Please provide a brief rationale for grouping different physical therapy modalities under the single category of physical therapy in the analysis. You need to show that it can be combined!

Response: We have added a brief rationale in the Methods section explaining our classification. We justified grouping these modalities under a single "physical therapy" category because they share common physiological goals (e.g., pain relief, reducing inflammation) and represent standard conventional care that is routinely combined with manual therapy in real-world clinical settings.

[Method] – [Operational Definitions of Interventions]

… a manual technique aimed at correcting the position of the humeral head within the glenoid fossa during movement to reduce subacromial impingement. These techniques, while sharing the overarching goal of restoring mobility, operate through distinct biomechanical and neurophysiological mechanisms, contributing to conceptual heterogeneity.

→ … a manual technique aimed at correcting the position of the humeral head within the glenoid fossa during movement to reduce subacromial impingement. These techniques, while sharing the overarching goal of restoring mobility, operate through distinct biomechanical and neurophysiological mechanisms, contributing to conceptual heterogeneity. Various modalities such as TENS, ultrasound, and heat therapy were grouped under a single 'physical therapy' category for analysis, as they share the common physiological goals of standard conservative care (e.g., pain relief, tissue healing) and are routinely administered together in clinical settings.

Point 5: (Line 89) The inclusion of database URLs in parentheses is unnecessary and can be removed.

Response: The database URLs in parentheses have been removed.

[Method] – [Database selection and search]

PubMed (www.pubmed.com), Embase (www.embase.com), and the Cochrane Library (www.thecochranelibrary.com) databases were searched

→ PubMed (www.pubmed.com), Embase (www.embase.com), and the Cochrane Library (www.thecochranelibrary.com) databases were searched

Point 6: (Line 93) The term “mobilization” appears to be repeated; please correct for clarity.

Response: We have corrected the repetition of the term "mobilization".

[Method] – [Database selection and search]

These English-language online databases were searched using a combination of the following search terms: “shoulder impingement syndrome,” “rotator cuff,” “impingement,” “subacromial impingement,” “mobilization,” and “mobilization,” with search formulas modified to each database.

→ These English-language online databases were searched using a combination of the following search terms: “shoulder impingement syndrome,” “rotator cuff,” “impingement,” “subacromial impingement,” “mobilization,” and “mobilization,” with search formulas modified to each database.

Point 7: (Line 109) The phrase “received interventions overlapping with mobilization that could not be separated” is unclear, particularly since studies including mobilization plus other interventions were allowed under the inclusion criteria. Please clarify what is meant by “overlapping” and how “could not be separated” was operationally defined.

Response: We have clarified this phrase. We operationally defined "overlapping and could not be separated" to mean instances where the comparison group received a complex manual therapy package that included elements of joint manipulation, making it impossible to isolate the specific effect of the experimental mobilization.

[Method] – [Exclusion criteria]

(3) Control (C): The comparison group received interventions overlapping with mobilization that could not be separated, or no appropriate control group was provided.

→ (3) Control (C): The comparison group received interventions that overlapped with mobilization that could not be separated (e.g., complex manual therapy packages where the specific effects of mobilization could not be isolated), or no appropriate control group was provided.

Point 8: (Line 116) The phrase “or study design” may be misleading, as only randomized controlled trials were included. Please revise to reflect that study design was, in fact, restricted to RCTs.

Response: We have revised this sentence to remove the misleading phrase "or study design." The text now explicitly states that the study design was strictly restricted to RCTs.

[Method] – [Exclusion criteria]

In the retrieved articles, no restrictions were applied regarding participants (age, sex), interventions (types or techniques of mobilization), comparisons (composition of the control group), outcomes (indices used for assessment), or study characteristics (treatment duration, follow-up period, or study design) in the retrieved articles.

→ In the retrieved articles, no restrictions were applied regarding participants (age, sex), interventions (types or techniques of mobilization), comparisons (composition of the control group), outcomes (indices used for assessment), or study characteristics (treatment duration, follow-up period, or study design) in the retrieved articles.

Point 9: The Results section provides detailed descriptions of individual trials prior to presenting pooled estimates. Consider condensing trial-level narrative and placing greater emphasis on meta-analytic results, as study-level details are already summarized in tables.

Response: We appreciate this constructive feedback. We have condensed the trial-level descriptive narratives in the Results section, as these details are already provided in the tables, and placed greater emphasis on reporting the meta-analytic results.

[Result] – [Treatment effects]

A between-group analysis was performed in eight studies comparing mobilization plus specific physical therapy with specific physical therapy alone [9, 10, 19, 28-30, 32, 33]. For the sake of simplicity, we will refer to the group that added physical therapy to mobilization as the add-on group and the group that performed physical therapy alone as the PT group in this section. Akhtar et al. [10], who analyzed the VAS and UCLA rating score, and Menek et al. [32], who analyzed the VAS, AROM, DASH, and SF-36, both reported that the add-on group was more effective than the PT group. Conroy and Hayes [19] and Neelapala et al. [33] reported that the add-on group was more effective in the VAS, but no statistically significant difference was found between the two groups in the AROM. Neelapala et al. [33] also found a significant effect of the add-on group on muscle strength. Eliason et al. [9] showed that pain improvement in flexion was greater in the add-on group in the VAS. However, for abduction, external rotation, and internal rotation, the improvement was greater in the PT group, and in the Constant-Murley score, no statistically significant difference was found between the two groups. İğrek and Çolak [29] reported that the add-on group was more effective in some indicators of VAS, DASH, Constant-Murley score, AROM, and muscle strength. Gutiérrez-Espinoza et al. [28], who analyzed the DASH, Constant-Murley score, VAS, and scapular UR, and Kachingwe et al. [30], who analyzed the VAS, AROM, and SPADI, found no statistically significant differences between the two groups.

→ A between-group analysis was performed in eight studies comparing mobilization plus specific physical therapy with specific physical therapy alone [9, 10, 19, 28-30, 32, 33]. For the sake of simplicity, we will refer to the group that added physical therapy to mobilization as the add-on group and the group that performed physical therapy alone as the PT group in this section. For simplicity, in this section we refer to the group that received mobilization in addition to physical therapy as the add-on group, and the group that received physical therapy alone as the PT group. Across these trials, several studies reported greater improvements in pain, function, and, in some cases, muscle strength in the add-on group, whereas others found no statistically significant differences between the add-on and PT groups on key outcomes such as pain, ROM, a

Attachment Submitted filename: Response to Reviewers_R2.docx

# Peer review: Decision Letter 3

**DOI:** 10.1371/journal.pone.0352260.r007

**Authors:** Sahreen Anwar, Saumya Srivastava, Saumya Srivastava, Saumya Srivastava

2 Apr 2026

PONE-D-24-07775R3

Clinical efficacy of joint mobilization for shoulder impingement syndrome: a systematic review and meta-analysis

PLOS One

Dear Dr. Ha,

Thank you for submitting your manuscript to PLOS ONE. After careful consideration, we feel that it has merit but does not fully meet PLOS ONE’s publication criteria as it currently stands. Therefore, we invite you to submit a revised version of the manuscript that addresses the points raised during the review process.

- Please briefly comment on how the high heterogeneity affects clinical applicability of the findings.
- Consider adding a short paragraph outlining practical clinical implications (e.g., patient subgroups or scenarios where mobilization may be most beneficial).
- Where possible, briefly comment on whether observed effects reach clinically meaningful thresholds (e.g., MCID).
- Further streamline the Results section by minimizing repetition of individual study findings and emphasizing pooled outcomes.

Please submit your revised manuscript by May 16 2026 11:59PM. If you will need more time than this to complete your revisions, please reply to this message or contact the journal office at plosone@plos.org. When you're ready to submit your revision, log on to https://www.editorialmanager.com/pone/ and select the 'Submissions Needing Revision' folder to locate your manuscript file.

Please include the following items when submitting your revised manuscript:

- A letter that responds to each point raised by the academic editor and reviewer(s). You should upload this letter as a separate file labeled 'Response to Reviewers'.
- A marked-up copy of your manuscript that highlights changes made to the original version. You should upload this as a separate file labeled 'Revised Manuscript with Track Changes'.
- An unmarked version of your revised paper without tracked changes. You should upload this as a separate file labeled 'Manuscript'.

If you would like to make changes to your financial disclosure, please include your updated statement in your cover letter. Guidelines for resubmitting your figure files are available below the reviewer comments at the end of this letter.

If applicable, we recommend that you deposit your laboratory protocols in protocols.io to enhance the reproducibility of your results. Protocols.io assigns your protocol its own identifier (DOI) so that it can be cited independently in the future. For instructions see: https://journals.plos.org/plosone/s/submission-guidelines#loc-laboratory-protocols. Additionally, PLOS ONE offers an option for publishing peer-reviewed Lab Protocol articles, which describe protocols hosted on protocols.io. Read more information on sharing protocols at https://plos.org/protocols?utm_medium=editorial-email&utm_source=authorletters&utm_campaign=protocols.

As the corresponding author, your ORCID iD is verified in the submission system and will appear in the published article. PLOS supports the use of ORCID, and we encourage all coauthors to register for an ORCID iD and use it as well. Please encourage your coauthors to verify their ORCID iD within the submission system before final acceptance, as unverified ORCID iDs will not appear in the published article. *Only*  the individual author can complete the verification step; PLOS staff *cannot*  verify ORCID iDs on behalf of authors.

We look forward to receiving your revised manuscript.

Kind regards,

Dr. Saumya Srivastava, Ph.D.

Academic Editor

PLOS One

Journal Requirements:

Please ensure all remaining Reviewer comments are fully addressed in your revised manuscript and point-by-point response document.

If the reviewer comments include a recommendation to cite specific previously published works, please review and evaluate these publications to determine whether they are relevant and should be cited. There is no requirement to cite these works unless the editor has indicated otherwise.

Please review your reference list to ensure that it is complete and correct. If you have cited papers that have been retracted, please include the rationale for doing so in the manuscript text, or remove these references and replace them with relevant current references. Any changes to the reference list should be mentioned in the rebuttal letter that accompanies your revised manuscript. If you need to cite a retracted article, indicate the article’s retracted status in the References list and also include a citation and full reference for the retraction notice.

Additional Editor Comments:

Dear Dr. Ha

Thank you for submitting the revised version of your manuscript entitled “Clinical efficacy of joint mobilization for shoulder impingement syndrome: a systematic review and meta-analysis” (Manuscript ID: PONE-D-24-07775).

I appreciate the thorough and thoughtful revisions made in response to the reviewers’ and editorial comments. The manuscript has improved substantially in terms of methodological clarity, transparency, and balance of interpretation. In particular, the clarification of heterogeneity, justification of statistical methods, and appropriate tempering of conclusions are commendable.

Based on the current revision, I am pleased to inform you that your manuscript is acceptable for publication in PLOS ONE. Before final acceptance, I request that you address the following minor points to further strengthen the manuscript:such as-

Add a short paragraph outlining practical clinical implication, please briefly comment on how the high heterogeneity affects clinical applicability of the findings. Please review the manuscript for minor grammatical issues.

Once these points are addressed, your manuscript will be ready for final acceptance and production.

Thank you for your valuable contribution to the field.

Sincerely,

Dr Saumya Srivastava,

Academic Editor

PLOS ONE

[Note: HTML markup is below. Please do not edit.]

Reviewers' comments:

Reviewer's Responses to Questions

**Comments to the Author**

1. If the authors have adequately addressed your comments raised in a previous round of review and you feel that this manuscript is now acceptable for publication, you may indicate that here to bypass the “Comments to the Author” section, enter your conflict of interest statement in the “Confidential to Editor” section, and submit your "Accept" recommendation.

Reviewer #4: All comments have been addressed

Reviewer #5: (No Response)

**********

2. Is the manuscript technically sound, and do the data support the conclusions?

The manuscript must describe a technically sound piece of scientific research with data that supports the conclusions. Experiments must have been conducted rigorously, with appropriate controls, replication, and sample sizes. The conclusions must be drawn appropriately based on the data presented.

Reviewer #4: Yes

Reviewer #5: Yes

**********

3. Has the statistical analysis been performed appropriately and rigorously?

Reviewer #4: Yes

Reviewer #5: Yes

**********

4. Have the authors made all data underlying the findings in their manuscript fully available?

The PLOS Data policy requires authors to make all data underlying the findings described in their manuscript fully available without restriction, with rare exception (please refer to the Data Availability Statement in the manuscript PDF file). The data should be provided as part of the manuscript or its supporting information, or deposited to a public repository. For example, in addition to summary statistics, the data points behind means, medians and variance measures should be available. If there are restrictions on publicly sharing data—e.g. participant privacy or use of data from a third party—those must be specified.

Reviewer #4: Yes

Reviewer #5: Yes

**********

5. Is the manuscript presented in an intelligible fashion and written in standard English?

PLOS ONE does not copyedit accepted manuscripts, so the language in submitted articles must be clear, correct, and unambiguous. Any typographical or grammatical errors should be corrected at revision, so please note any specific errors here.

Reviewer #4: Yes

Reviewer #5: Yes

**********

6. Review Comments to the Author

Please use the space provided to explain your answers to the questions above. You may also include additional comments for the author, including concerns about dual publication, research ethics, or publication ethics. (Please upload your review as an attachment if it exceeds 20,000 characters)

Reviewer #4: (No Response)

Reviewer #5: 1. Suggestion Title: Consider whether “joint mobilization” accurately reflects the umbrella definition you later use (including neuromobilization and soft tissue mobilization). The title may slightly underrepresent intervention heterogeneity.

2. Minor suggestion in Abstract (background): Replace “clinical evidence” with “clinical effectiveness” for consistency.

3. Important issue in Abstract (Methods and Result): You report mean differences (MD) but do not indicate scale units (e.g., VAS 0–10? 0–100?). Clinical interpretability is limited. No heterogeneity (I²) values were reported in the abstract despite very high I² in the results.

4. Suggestion in Introduction (Rationale): Add 1–2 sentences explaining why pooling heterogeneous mobilization types is clinically meaningful (you later discuss heterogeneity, but early framing helps).

5. Minor suggestion in Methods (operational definition): When grouping TENS, ultrasound, and heat under “physical therapy,” add 1 citation supporting this classification as standard conservative care.

6. Results General comments:

Major Concern: Very High Heterogeneity (I² > 90%)

This is the most critical methodological issue.

When I² > 90%:

Pooling may not be clinically meaningful.

Summary estimate precision is compromised.

You now appropriately caution good interpretation.

However, consider adding:

Exploration of heterogeneity (subgroup by type of mobilization? dosage? acute vs chronic?)

Meta-regression (if feasible).

7. Discussion part:

i) Discuss clinical significance (not just statistical significance).

Is MD=1.72 on VAS clinically meaningful?

Minimal clinically important difference (MCID) discussion recommended.

ii) Clarify duration of effect (short-term only?).

ii) Explicitly state that evidence certainty is low (if GRADE was not performed, acknowledge that certainty grading was not conducted).

**********

7. PLOS authors have the option to publish the peer review history of their article (what does this mean?). If published, this will include your full peer review and any attached files.

If you choose “no”, your identity will remain anonymous but your review may still be made public.

**Do you want your identity to be public for this peer review?**  For information about this choice, including consent withdrawal, please see our Privacy Policy.

Reviewer #4: No

Reviewer #5: No

**********

[NOTE: If reviewer comments were submitted as an attachment file, they will be attached to this email and accessible via the submission site. Please log into your account, locate the manuscript record, and check for the action link "View Attachments". If this link does not appear, there are no attachment files.]

To ensure your figures meet our technical requirements, please review our figure guidelines: https://journals.plos.org/plosone/s/figures

You may also use PLOS’s free figure tool, NAAS, to help you prepare publication quality figures: https://journals.plos.org/plosone/s/figures#loc-tools-for-figure-preparation.

NAAS will assess whether your figures meet our technical requirements by comparing each figure against our figure specifications.

# Author comment: Author response to Decision Letter 4

**DOI:** 10.1371/journal.pone.0352260.r008

23 Apr 2026

Dear Dr. Saumya Srivastava, Academic Editor, PLOS ONE,

Thank you for the opportunity to submit a further revised version of our manuscript entitled “Clinical efficacy of joint mobilization for shoulder impingement syndrome: a systematic review and meta-analysis” (Manuscript ID: PONE-D-24-07775R3).

We sincerely appreciate your positive evaluation of our manuscript and the helpful comments provided by you and the reviewers. We have carefully revised the manuscript to address all remaining points. Below, we provide a point-by-point response.

Academic Editor

Comment 1. Please briefly comment on how the high heterogeneity affects clinical applicability of the findings.

Response:

Thank you for this important comment. We have revised the Discussion section to more explicitly explain how the substantial heterogeneity affects clinical applicability. We clarified that the high I² values observed in several analyses indicate considerable variability in treatment effects, suggesting that the benefits of mobilization may not be consistent across all clinical contexts. We also noted that this variability likely reflects differences in mobilization techniques, treatment dosage, co-interventions, control conditions, and patient characteristics. Therefore, we emphasized that the findings should not be considered universally generalizable to all patients with shoulder impingement syndrome and that clinical application should be individualized.

Revised text:

“No adverse events were reported in the included studies. However, the results of this meta-analysis should be interpreted with caution due to substantial statistical heterogeneity, largely attributable to clinical diversity among studies. High I² values (often exceeding 90%) indicate considerable variability in treatment effects, suggesting that the benefits of mobilization are not consistent across all clinical contexts. This variability likely arises from differences in mobilization techniques, treatment dosage, co-interventions, control conditions, and patient characteristics. Consequently, the findings are not universally generalizable to all patients with shoulder impingement syndrome, and clinical application should be individualized. The heterogeneity also limits the precision of pooled estimates, reflecting the context-dependent nature of mobilization in practice.”

Comment 2. Consider adding a short paragraph outlining practical clinical implications.

Response:

Thank you for this helpful suggestion. We added a new paragraph under the “Clinical implications” section. In this paragraph, we clarified that joint mobilization may be considered as an adjunctive intervention, particularly for patients with limited range of motion, pain-related movement restriction, or difficulty engaging in active exercise-based rehabilitation. We also emphasized that treatment should be individualized according to clinical presentation and treatment context.

Revised text:

“From a clinical perspective, joint mobilization can be considered an adjunctive intervention for patients with shoulder impingement syndrome (SIS), particularly for those presenting with limited range of motion, pain-related movement restriction, or difficulty engaging in active exercise-based rehabilitation. It may facilitate early pain reduction and improve joint mobility, thereby promoting participation in subsequent active therapy. However, given the heterogeneity in intervention protocols and patient characteristics across studies, its application should be individualized according to the patient’s clinical presentation and treatment context.”

Comment 3. Where possible, briefly comment on whether observed effects reach clinically meaningful thresholds, such as MCID.

Response:

We appreciate this important comment. We added a brief discussion of clinical meaningfulness in addition to statistical significance. Specifically, we noted that the magnitude of pain reduction observed in this review may approach commonly cited MCID thresholds for shoulder pain. However, we interpreted this cautiously because outcome scales and study contexts varied across trials, and high heterogeneity limits definitive conclusions regarding clinical relevance.

Revised text:

“Beyond statistical significance, the clinical meaningfulness of the observed effects should be considered. For example, the magnitude of pain reduction observed in this study may approach commonly cited minimal clinically important difference (MCID) thresholds for shoulder pain. However, interpretation should remain cautious, as outcome measures (e.g., VAS 0–10 vs. 0–100) and study contexts varied across trials. Furthermore, the high heterogeneity limits the ability to draw definitive conclusions regarding the clinical relevance of these pooled estimates.”

Comment 4. Further streamline the Results section by minimizing repetition of individual study findings and emphasizing pooled outcomes.

Response:

Thank you for this constructive comment. We carefully revised the Results section to reduce repeated descriptions of individual trial findings and to place greater emphasis on pooled outcomes. In the previous version, several subsections included repeated study-level descriptions before or alongside the pooled estimates. In the revised manuscript, we retained essential information on the included comparisons while condensing repetitive narratives and presenting the main findings in a more concise, synthesis-oriented manner.

Specifically, we made the following changes:

1. In the comparison between mobilization plus physical therapy and physical therapy alone, we condensed the repeated study-level descriptions into an overall summary of the direction of findings. We then presented the pooled VAS, AROM, DASH, and Constant-Murley results more directly.

2. For VAS subgroup analyses, we combined repeated descriptions of VAS at rest, during activity, and across all days into a single concise paragraph while retaining the key MD, 95% CI, p value, and I² values.

3. For AROM subgroup analyses, we integrated the movement-specific results into one paragraph, reporting which movements showed significant improvement and which did not, while preserving the relevant pooled estimates and heterogeneity values.

4. In the comparison between mobilization plus physical therapy and exercise plus physical therapy, we reduced repeated individual-study narrative and summarized the overall pattern of findings before presenting the pooled VAS and ROM outcomes.

5. In the comparison between mobilization and sham mobilization, we retained the essential study-level context but condensed repeated subgroup descriptions. We also preserved the key pooled effects for VAS and AROM and retained the explanation for the “not estimable” study.

6. In the remaining comparisons, including comparisons between different mobilization approaches, mobilization versus other treatments, timing of intervention, and mobilization after SACS injection, we shortened repetitive wording while maintaining the original meaning and key findings.

Revised examples:

“A meta-analysis of eight studies using VAS immediately after treatment showed a statistically significant reduction in pain in the add-on group compared with the PT group (MD=1.72 [95% CI 0.91, 2.53], p<0.00001, I²=97%). Given the high heterogeneity, these results should be interpreted with caution. Subgroup analyses of VAS at different time points showed no statistically significant difference at rest (MD=0.58 [95% CI -0.16, 1.32], p=0.12, I²=78%), whereas significant improvements were observed during activity (MD=1.67 [95% CI 0.21, 3.14], p=0.03, I²=98%) and across all days (MD=3.00 [95% CI 2.78, 3.22], p<0.00001, I²=0%).”

“A meta-analysis of four studies using AROM immediately after treatment demonstrated a statistically significant improvement in the add-on group (MD=14.49 [95% CI 8.46, 20.53], p<0.00001, I²=95%). Subgroup analyses by movement direction showed significant improvements in flexion, internal rotation, and abduction, whereas no statistically significant differences were observed in extension and external rotation. Most subgroup analyses showed substantial heterogeneity, warranting cautious interpretation.”

“A meta-analysis of two studies using the VAS immediately after treatment demonstrated a statistically significant reduction in pain in the mobilization group (MD=0.67 [95% CI 0.15, 1.19], p=0.01, I²=0%). Subgroup analyses by time point showed no statistically significant differences at rest or during activity, with generally low heterogeneity. One study was labeled as ‘not estimable’ in part of the analysis due to insufficient variance information.”

We believe these revisions improve readability while preserving the essential quantitative findings and interpretive cautions.

Comment 5. Please review the manuscript for minor grammatical issues.

Response:

We have carefully reviewed the manuscript and corrected minor grammatical and typographical issues throughout the text.

Reviewer #5

Comment 1. Suggestion Title: Consider whether “joint mobilization” accurately reflects the umbrella definition you later use, including neuromobilization and soft tissue mobilization. The title may slightly underrepresent intervention heterogeneity.

Response:

Thank you for this thoughtful suggestion. We carefully considered whether the title should be changed. Because the term “joint mobilization” is widely used in the clinical and rehabilitation literature and is consistent with the main focus of most included studies, we retained the current title to preserve clarity and continuity. However, to address the concern that the term may underrepresent intervention heterogeneity, we clarified in the Introduction and Methods that mobilization was treated as a broader clinical category that included joint mobilization, neuromobilization, soft tissue mobilization, mobilization with movement, and dynamic humeral centering.

Comment 2. Minor suggestion in Abstract: Replace “clinical evidence” with “clinical effectiveness” for consistency.

Response:

Thank you for this suggestion. We revised the Abstract Background accordingly.

Revised text:

“We aimed to evaluate clinical effectiveness for the use of mobilization in SIS.”

Comment 3. Important issue in Abstract: You report mean differences (MD) but do not indicate scale units. Clinical interpretability is limited. No heterogeneity (I²) values were reported in the abstract despite very high I² in the results.

Response:

Thank you for pointing this out. To improve interpretability while maintaining the conciseness required for the Abstract, we added I² values for the two primary outcomes with very high heterogeneity: pain and active range of motion. We retained the key effect estimates and confidence intervals and avoided overloading the Abstract with too many heterogeneity values.

Revised text:

“Adding mobilization to traditional physical therapy reduced pain (mean difference [MD]=1.72, 95% confidence interval [CI]: 0.91 to 2.53, I²=97%, p<0.00001), increased active range of motion (MD=14.49, 95% CI: 8.46 to 20.53, I²=95%, p<0.00001)…”

Comment 4. Suggestion in Introduction: Add 1–2 sentences explaining why pooling heterogeneous mobilization types is clinically meaningful.

Response:

Thank you for this helpful suggestion. We added two sentences at the end of the Introduction to explain why evaluating heterogeneous mobilization techniques as a broader clinical category is clinically meaningful.

Revised text:

“Although mobilization techniques differ in their specific biomechanical and neurophysiological mechanisms, they share a common therapeutic goal of improving pain and movement dysfunction. Therefore, evaluating mobilization as a broader clinical category may provide useful insight into its overall role as an adjunctive treatment in patients with shoulder impingement syndrome.”

Comment 5. Minor suggestion in Methods: When grouping TENS, ultrasound, and heat under “physical therapy,” add one citation supporting this classification as standard conservative care.

Response:

Thank you for this suggestion. We carefully reviewed this point. As this was a minor suggestion rather than a required editorial change, and because the grouping was used as an operational classification for the meta-analysis, we retained the current wording without adding a new citation. We clarified in the Methods section that TENS, ultrasound, and heat therapy were grouped under “physical therapy” because they share common physiological goals of standard conservative care and are routinely administered together in clinical practice.

Relevant text:

“Various modalities such as TENS, ultrasound, and heat therapy were grouped under a single ‘physical therapy’ category for analysis, as they share the common physiological goals of standard conservative care (e.g., pain relief, tissue healing) and are routinely administered together in clinical settings.”

Comment 6. Results General comments: Major concern: Very high heterogeneity (I² > 90%). Consider adding exploration of heterogeneity, subgroup by type of mobilization, dosage, acute vs chronic, or meta-regression if feasible.

Response:

We agree that very high heterogeneity is an important methodological issue in this review. In the revised manuscript, we further clarified the clinical implications of high heterogeneity and emphasized that the pooled estimates should be interpreted cautiously. We explained that the variability likely arises from differences in mobilization techniques, dosage, co-interventions, control conditions, and patient characteristics.

We also considered additional subgroup analyses and meta-regression. However, these analyses were not statistically feasible because the number of studies in each specific comparison or technique category was small, and many interventions were combined with other physical therapy components. Therefore, we addressed this issue in the Discussion and Limitations sections rather than performing potentially underpowered analyses.

Revised text:

“High I² values (often exceeding 90%) indicate considerable variability in treatment effects, suggesting that the benefits of mobilization are not consistent across all clinical contexts. This variability likely arises from differences in mobilization techniques, treatment dosage, co-interventions, control conditions, and patient characteristics. Consequently, the findings are not universally generalizable to all patients with shoulder impingement syndrome, and clinical application should be individualized.”

Comment 7. Discussion: Discuss clinical significance, not just statistical significance. Is MD=1.72 on VAS clinically meaningful? MCID discussion recommended.

Response:

Thank you for this important recommendation. We added a paragraph discussing clinical meaningfulness in addition to statistical significance. We noted that the observed magnitude of pain reduction may approach commonly cited MCID thresholds for shoulder pain, but we interpreted this cautiously because outcome measures and study contexts varied across trials and heterogeneity was high.

Revised text:

“Beyond statistical significance, the clinical meaningfulness of the observed effects should be considered. For example, the magnitude of pain reduction observed in this study may approach commonly cited minimal clinically important difference (MCID) thresholds for shoulder pain. However, interpretation should remain cautious, as outcome measures (e.g., VAS 0–10 vs. 0–100) and study contexts varied across trials. Furthermore, the high heterogeneity limits the ability to draw definitive conclusions regarding the clinical relevance of these pooled estimates.”

Comment 8. Discussion: Clarify duration of effect.

Response:

Thank you for this comment. We maintained and reinforced the interpretation that the observed benefits should be understood primarily as short-term and adjunctive. This point is stated in both the Abstract Conclusion and the main Conclusion.

Relevant text:

“Mobilization may offer adjunctive, short-term benefits in improving the clinical symptoms of SIS when added to conventional treatments.”

“These benefits appear to be primarily short-term and

Attachment Submitted filename: Response to Reviewers_R3.docx

# Editor report: Decision Letter 4

**DOI:** 10.1371/journal.pone.0352260.r009

**Authors:** Sahreen Anwar, Saumya Srivastava, Saumya Srivastava, Saumya Srivastava, Saumya Srivastava

8 Jun 2026

Clinical efficacy of joint mobilization for shoulder impingement syndrome: a systematic review and meta-analysis

PONE-D-24-07775R4

Dear Dr. Ha,

We’re pleased to inform you that your manuscript has been judged scientifically suitable for publication and will be formally accepted for publication once it meets all outstanding technical requirements.

Within one week, you’ll receive an e-mail detailing the required amendments. When these have been addressed, you’ll receive a formal acceptance letter and your manuscript will be scheduled for publication.

An invoice will be generated when your article is formally accepted. Please note, if your institution has a publishing partnership with PLOS and your article meets the relevant criteria, all or part of your publication costs will be covered. Please make sure your user information is up-to-date by logging into Editorial Manager at Editorial Manager® and clicking the ‘Update My Information' link at the top of the page. For questions related to billing, please contact billing support.

If your institution or institutions have a press office, please notify them about your upcoming paper to help maximize its impact. If they’ll be preparing press materials, please inform our press team as soon as possible -- no later than 48 hours after receiving the formal acceptance. Your manuscript will remain under strict press embargo until 2 pm Eastern Time on the date of publication. For more information, please contact onepress@plos.org.

Kind regards,

Saumya Srivastava, Ph.D.

Academic Editor

PLOS One

Additional Editor Comments (optional):

Dear Dr. Ha,

Thank you for submitting the revised version of your manuscript entitled "Clinical efficacy of joint mobilization for shoulder impingement syndrome: a systematic review and meta-analysis."

The revised manuscript has been carefully evaluated together with your detailed responses to the comments raised during the previous review rounds. The revisions have substantially improved the manuscript, particularly with respect to the discussion of heterogeneity, clinical applicability, clinical significance of findings, reporting transparency, and overall readability.

I appreciate the thorough manner in which you have addressed the concerns raised by the reviewers and the Academic Editor.

I am pleased to inform you that the manuscript is considered suitable for publication in PLOS ONE and is recommended for acceptance.

Thank you for your careful revisions and for choosing PLOS ONE for the dissemination of your work.

Kind regards,

Dr. Saumya Srivastava

Academic Editor

PLOS ONE

Reviewers' comments:

# Editor report: Acceptance letter

**DOI:** 10.1371/journal.pone.0352260.r010

**Authors:** Sahreen Anwar, Saumya Srivastava, Saumya Srivastava, Saumya Srivastava, Saumya Srivastava

PONE-D-24-07775R4

PLOS One

Dear Dr. Ha,

I'm pleased to inform you that your manuscript has been deemed suitable for publication in PLOS One. Congratulations! Your manuscript is now being handed over to our production team.

At this stage, our production department will prepare your paper for publication. This includes ensuring the following:

* All references, tables, and figures are properly cited

* All relevant supporting information is included in the manuscript submission,

* There are no issues that prevent the paper from being properly typeset

You will receive further instructions from the production team, including instructions on how to review your proof when it is ready. Please keep in mind that we are working through a large volume of accepted articles, so please give us a few days to review your paper and let you know the next and final steps.

Lastly, if your institution or institutions have a press office, please let them know about your upcoming paper now to help maximize its impact. If they'll be preparing press materials, please inform our press team within the next 48 hours. Your manuscript will remain under strict press embargo until 2 pm Eastern Time on the date of publication. For more information, please contact onepress@plos.org.

You will receive an invoice from PLOS for your publication fee after your manuscript has reached the completed accept phase. If you receive an email requesting payment before acceptance or for any other service, this may be a phishing scheme. Learn how to identify phishing emails and protect your accounts at https://explore.plos.org/phishing.

If we can help with anything else, please email us at customercare@plos.org.

Thank you for submitting your work to PLOS ONE and supporting open access.

Kind regards,

PLOS ONE Editorial Office Staff

on behalf of

Dr Saumya Srivastava

Academic Editor

PLOS One
